# Study into the reversal of septic shock with landiolol (beta blockade): STRESS-L Study protocol for a randomised trial

**DOI:** 10.1136/bmjopen-2020-043194

**Published:** 2021-02-16

**Authors:** Ranjit Lall, Dipesh Mistry, Emma Skilton, Nafisa Boota, Scott Regan, Julian Bion, Simon Gates, Anthony C Gordon, Janet Lord, Daniel Francis McAuley, Gavin Perkins, Mervyn Singer, Duncan Young, Tony Whitehouse

**Affiliations:** 1 Warwick Clinical Trials Unit, University of Warwick, Coventry, UK; 2 Leicester Clinical Trials Unit, Leicester, UK; 3 Intensive Care Medicine, University of Birmingham, Birmingham, UK; 4 Cancer Research UK Clinical Trials Unit, University of Birmingham, Birmingham, UK; 5 Division of Anaesthetics, Pain Medicine and Intensive Care, Imperial College London, London, UK; 6 NIHR Surgical Reconstruction and Microbiology Research Centre, University of Birmingham, Birmingham, UK; 7 Centre for Experimental Medicine, Queen's University Belfast, Belfast, UK; 8 University Hospitals Birmingham NHS Foundation Trust, Birmingham, UK; 9 Bloomsbury Institute of Intensive Care Medicine, University College London, London, UK; 10 Kadoorie Centre for Critical Care Research, Nuffield Division of Anaesthesia, University of Oxford, Oxford, UK; 11 Department of Critical Care and Anaesthesia, University Hospitals Birmingham NHS Foundation Trust, Birmingham, UK; 12 Institute of Inflammation and Aging, University of Birmingham, Birmingham, UK

**Keywords:** adult intensive & critical care, microbiology, statistics & research methods, clinical trials

## Abstract

**Introduction:**

In 2013, a single-centre study reported the safe use of esmolol in patients with septic shock and tachycardia who required vasopressor therapy for more than 24 hours. Although not powered to detect a change in mortality, marked improvements were seen in survival (adjusted HR, 0.39; 95% CI, 0.26 to 0.59; p<0.001). Beta blockers are one of the most studied groups of drugs but their effect in septic shock is poorly understood; proposed mechanisms include not only the modulation of cardiac function but also immunomodulation.

**Methods and analysis:**

STRESS-L is a randomised, open-label, non-blinded clinical trial which is enrolling a total of 340 patients with septic shock as defined by Sepsis-3 consensus definition and a tachycardia (heart rate ≥95 beats per minute (bpm)) after vasopressor treatment of at least 24 hours. Standard randomisation (1:1 ratio) allocates patients to receive usual care (according to international standards) versus usual care and a continuous landiolol infusion to reduce the heart rate between 80 and 94 bpm. The primary endpoint is the mean Sequential Organ Failure Assessment score over 14 days from entry into the trial and while in intensive care unit. Results will inform current clinical practice guidelines.

**Ethics and dissemination:**

This trial has clinical trial authorisation from the UK competent authority, the Medicines and Healthcare products Regulatory Agency, and has been approved by the East of England-Essex Research Ethics Committee (reference: 17/EE/0368).

The results of the trial will be reported first to trial collaborators. The main report will be drafted by the trial coordinating team, and the final version will be agreed by the Trial Steering Committee before submission for publication, on behalf of the collaboration.

**Registration:**

The trial is funded by the National Institute for Health Research Efficacy and Mechanism Evaluation (EME) (Project Number: EME-14/150/85) and registered ISRCTN12600919 and EudraCT: 2017-001785-14.

**Strengths and limitations of this study**

- STRESS-L is a randomised study comparing the use of the ultra-short acting beta blocking agent, landiolol, with standard care in patients with septic shock, persistent tachycardia treated with high-dose norepinephrine.
- STRESS-L will study 340 patients, which we believe will be the largest study of its type.
- There is little known about the effects of beta blockade and the mechanisms induced by beta-1 receptor antagonist in this population.
- Its limitation is that the study is not double blind since the dosing of the investigational medicinal product (landiolol) is variable according to a physiological endpoint (target heart rate of 80–94 beats per minute), a placebo would be ineffective and the intervention impossible to blind.

## Introduction

A randomised single-centre study of 154 patients from Rome^1^ reported the use of beta-adrenergic blockade using the short-acting agent esmolol in patients with septic shock and tachycardia who required vasopressor therapy for more than 24 hours. The dosing endpoint was a reduction in heart rate between 80 and 94 beats per minute (bpm). Though this study was not powered to detect a change in mortality, marked improvements were seen in survival (adjusted HR, 0.39; 95% CI, 0.26 to 0.59; p<0.001). Improvements were also seen in the beta blocker-treated group in the duration of vasopressor treatment, and in measures of renal and cardiac function.

Autonomic dysfunction and tachycardia are associated with a poor outcome in septic shock^2^ with mortalities in excess of 70%.^3^ Bradycardia is relatively protective.^4^ Currently, beta agonists are recommended as part of resuscitation and restoration of cardiac output and blood pressure in septic shock^5^ despite an early study examining the use of dobutamine to increase cardiac index and systemic oxygen delivery being stopped prematurely because of an increase in the intervention cohort.^6^ Beta blockade represents a paradigm shift in management of patients with septic shock and arises from observations of harm induced by excessive sympathetic activation and benefit from beta-adrenergic blockade. Limited data from animal models^7–12^ and patients^1 13 14^ suggest improved cardiovascular and immune function by their use. Excess beta-adrenergic activation may be both endogenous (related to the severity and duration of the underlying infection) and exogenous due to the catecholamine therapy which has until now been the mainstay of management of hypotension caused by septic shock.^5^ A combination of beta-2 agonist with beta-1 blockade improves sepsis-induced immune, cardiovascular and coagulation dysfunction in established sepsis (reviewed in^15^).

Landiolol (Rapibloc, AOP Orphan Pharmaceuticals, Vienna, Austria) is an ultra-short-acting beta blocker that has a half-life of 2.3–4 min^16^ and is approximately eight times more selective for the beta-1 receptor than esmolol.^17^ Landiolol is metabolised by plasma pseudocholinesterase and liver carboxylesterse to inactive metabolites and its metabolism is unaffected by liver impairment.^18^ A few preclinical studies have examined landiolol in sepsis. It decreased circulating levels of the cytokines, tumour necrosis factor (TNF)-alpha, interleukin (IL)-6, and high mobility group box-1 and reduced histological lung damage in a rat endotoxin model.^11^ Landiolol was also cardioprotective in rats with septic shock by normalising the expression of cardiac vasoactive peptide endothelin-1.^19^ Landiolol has been used safely and successfully in a patient with septic shock with atrial fibrillation^20^ and in sepsis-induced tachycardia.^21^ A recent trial of 151 patients from 54 centres in Japan^22^ demonstrated that landiolol could be safely administered to patients with septic shock.

Landiolol rather than esmolol was selected for this clinical trial because of its superior beta-1 specificity and the expectation that stopping the landiolol infusion would allow a rapid return to the baseline haemodynamic state if investigators deemed the intervention harmful. We will also report data of safety of landiolol infusion as part of the study.

## Study objectives

### Primary objective

The primary objective of this trial is to assess the efficacy, safety and mechanisms of landiolol (beta blockade) in patients with septic shock and tachycardia requiring prolonged (>24-hour continuous treatment) support with high-dose vasopressor agents.

### Secondary objectives

The secondary objectives of the trial are:

- To determine whether infusion of the rapid-acting, ultra-short-lived and highly specific beta-adrenergic antagonist landiolol improves mortality and length of hospital stay compared with current best clinical practice, in patients who have septic shock.
- To investigate the pathways that are altered by beta blockade in septic shock by examining the effects of landiolol on blood markers of inflammation, metabolism and cardiomyocyte damage.

## Methods and analysis

This manuscript has been written in concordance with the Standard Protocol Items: Recommendations for Interventional Trials guidelines.^23^

### Trial design

This is a multicentre, randomised, controlled open label phase IIb trial comparing usual treatment with usual treatment plus landiolol infusion in a total of 340 patients. The trial is conducted and managed by Warwick Clinical Trials Unit and sponsored by the University Hospitals of Birmingham (UHB) National Health Service Foundation Trust. It is coordinated by a Trial Management Group and independent oversight is provided by the Trial Steering Committee and a Data Monitoring Committee.

The trial has been designed and will be reported in line with the CONSORT (Consolidated Standards of Reporting Trials) statement^24^ and the conduct has been planned in full conformance with the principles of the Declaration of Helsinki and Good Clinical Practice.

### Study setting

The study recruitment commenced in May 2018. The main trial is taking place in over 35 UK adult intensive care units (ICUs). Participating ICUs will have a typical case mix for UK critical care, a track record of recruitment to clinical trials, suitable support for screening and data collection, and the means to store blood samples for transfer to the central analysis unit. They should also be willing to manage atrial fibrillation with correction of potassium, magnesium and amiodarone in the usual care group as the use of beta blockers in these patients would risk making the trial results uninterpretable.

### Inclusion criteria

Patients are eligible to be included in the trial if they meet the following criteria:

- Aged 18 years or above.
- Being treated in a critical care unit.
- Septic shock according to internationally accepted definitions.*
- Heart rate ≥95 bpm (at the time of randomisation).
- Receiving vasopressor support to maintain a target blood pressure for ≥24 hours.
- Are being treated with norepinephrine at a rate ≥0.1 µg/kg/min.

*Sepsis-3 definitions^25^:

- Confirmed or suspected infection requiring antibiotic therapy.
- New organ dysfunction, as evidenced by an increase in Sequential Organ Failure Assessment (SOFA) score ≥2.
- A blood lactate >2 mmol/L at any point during shock resuscitation.
- Vasopressor therapy to maintain mean arterial pressure (MAP) ≥65 mm Hg.

In particular, the presence of a blood lactate >2 mmol/L is only necessary for the diagnosis of septic shock and is NOT necessary for randomisation 24 hours later.

### Exclusion criteria

The participant may not enter the trial if any of the following applies:

- Tachycardia as a result of pain, discomfort from medical devices (including endotracheal tubes), during interventions or other patient distress.
- Any form of vasodilatory shock that is not caused by sepsis.
- Norepinephrine infusion <0.1 µg/kg/min.
- >72 hours after start of vasopressor therapy.
- <12 hours since norepinephrine to treat a medical condition other than septic shock stopped.
- Having pre-existing severe cardiac dysfunction (New York Heart Association grade 4 or more).
- Having pre-existing severe pulmonary hypertension (mean pulmonary artery pressures >55 mm Hg).
- Acute severe bronchospasm (due to asthma or chronic obstructive pulmonary disease).
- Untreated second or third degree heart block.
- Untreated phaeochromocytoma.
- Prinzmetal’s angina.
- A history of ischaemic stroke or transient ischaemic attack or untreated severe carotid stenosis.
- Advanced liver disease with Child-Pugh score of ≥B.
- Known sensitivity to beta blockers.
- Patient/legal representative unwilling to provide written informed consent.
- Known to be pregnant.
- Terminal illness other than septic shock with a life expectancy <28 days.
- Participants who have been administered an investigational medicinal product (IMP) for another research trial in the past 30 days.
- Patients in whom the clinical team feel are about to finish their norepinephrine therapy.
- Decision of withdrawal of care is in place or imminently anticipated.
- Receiving extracorporeal membrane oxygenation treatment.

Co-enrolment of study participants onto other interventional studies will be considered where there is no possible conflict with the STRESS-L trial objectives.

### Consent

Eligible patients who are deemed competent will be provided with a copy of the patient information sheet before informed consent is sought. However, due to the nature of the underlying condition and its treatment, many patients will be unable to give informed consent. The vulnerability of this patient group is fully appreciated and every effort must be undertaken to protect their safety and well-being. To ensure this, consenting will be obtained in accordance with the Medicines for Human Use Regulations and the Health Research Authority ethics guide on medical research involving adults who cannot consent (http://www.hra.nhs.uk/resources/before-you-apply/consent-and-participation/adults-unable-to-consent-for-themselves/).

### Intervention

Participants will be randomised to either usual care (control group) or usual care and landiolol (intervention group).

#### Usual care

Participants randomised to this arm will receive usual care for septic shock, namely:

- Timely treatment of the source of sepsis (eg, drainage of infected fluid collections).
- Prompt and appropriate empirical antibiotic treatment, and modification, if needed, on the basis of culture results.
- Appropriate fluid resuscitation to correct hypovolaemia.
- The use of vasopressors to achieve a target MAP (suggested target 65–70 mm Hg).

Adequate fluid resuscitation will be achieved using repeated fluid challenges to a guided target; the use of a cardiac output monitor is not mandated. The usual care group will not receive any beta blockade for the duration of their ICU stay—if the treating clinician deems beta blockade necessary, this will be captured on the Case Report Form (CRF) and reported as a protocol deviation. All other general ICU management will be based on the latest guidance from the surviving sepsis campaign^5^ and the UK national critical care guidelines (eg, ventilator and central line care bundles).

#### Usual care and landiolol

An intravenous infusion of landiolol will be started at 1.0 µg/kg/min and will progressively increase every 15 min at increments of 1.0 µg/kg/min, to reach the target heart rate of 80–94 bpm usually within 6 hours. Landiolol will be administered peripherally or centrally but must be on a dedicated line. Landiolol has an elimination half-life of 2.3–4 min^17^ and so a loading dose is unnecessary. The landiolol infusion will be continued until the pulse rate is persistently below 95 bpm.

**Figure 1:**
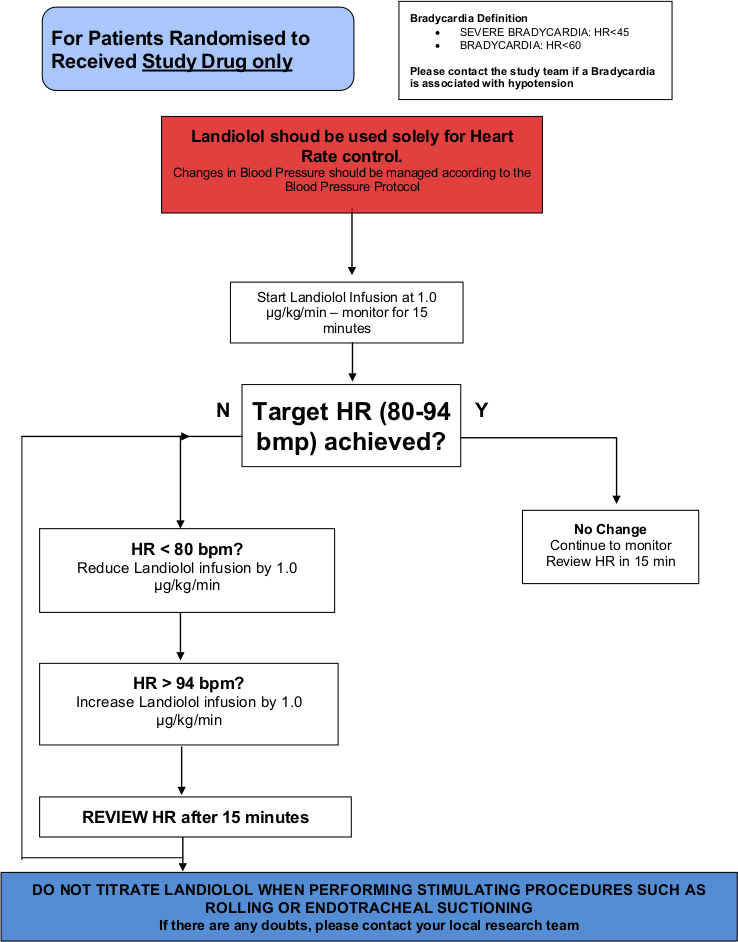
STRESS-L Study drug infusion protocol. bpm, beats per minute; HR, heart rate.

**Figure 2:**
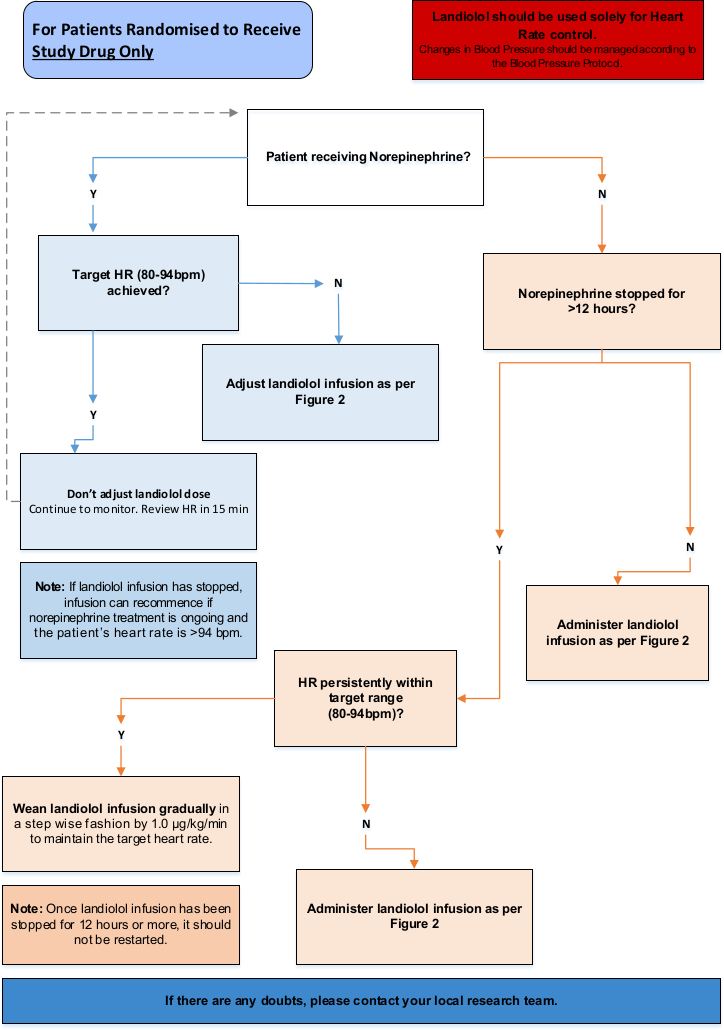
STRESS-L protocol for weaning and stopping of study drug. bpm, beats per minute; HR, heart rate.

While a patient is receiving vasopressor agents (norepinephrine, vasopressin), the landiolol infusion will be adjusted accordingly to maintain the target heart rate of 80–94 bpm as per the landiolol infusion protocol (figure 1: STRESS-L Study drug infusion protocol). Once the patient is consistently within the target heart rate of 80–94 bpm, the landiolol infusion will be continued and not be adjusted. The landiolol should be weaned and, if necessary, stopped while the heart rate is below 80 bpm. Once all vasopressor agents have been discontinued:

- If vasopressors have been discontinued for less than 12 hours, the landiolol infusion will continue according to figure 1.
- If vasopressors have been discontinued for more than 12 hours, the landiolol should be actively weaned (this is the end of norepinephrine treatment (EONT) timepoint) and according to figure 2: Timing and weaning of the study drug.

This trial allows for up to 14 days of landiolol treatment per participant.

## Outcome measures

### Primary outcome

The primary outcome will be the mean SOFA score over the first 14 days from entry into the trial and while in ICU. Measurement of the SOFA score will cease if the patient withdraws from the trial, dies or is discharged from the ICU. It is assumed that delayed discharges will be evenly distributed between each group.

### Secondary outcomes

- Mortality at day 28 and day 90.
- Length of ICU and hospital stay.
- Reduction in dose and duration of vasopressor treatment (total daily administered doses).

### Mechanistic outcomes

Serial blood samples will be collected from patients and assays will include markers of myocardial dysfunction and inflammation.

- Measurement of total catecholamine. It is unknown whether beta blockade acts through altering the effects of extraneous catecholamines administered as treatment of septic shock or by modulating those produced by the patient. Serum catecholamines will be analysed in the context of the dose of inotropes being administered.
- Markers of myocardial dysfunction. Serum B-type natriuretic peptide has been demonstrated to be a reliable biomarker of myocardial injury, ischaemia and dysfunction in patients with septic shock and also as a prognostic marker for a poor outcome.^26 27^ Serial measurements of troponin-T will be made.
- Measurement of serum free fatty acids and markers of fatty acid metabolism.
- Biomarkers of systemic inflammation. This will be measured using a multiplex inflammatory biomarker assay depending on the available technology at the time of analysis. A selection of cytokines will be analysed, all or some of which will include IL-1 beta, IL-2, IL-4, IL-5, IL-6, IL-8, IL-10, IL-12, TNF-alpha, TNF-beta and interferon-gamma. This focused array will allow an assessment of the pro/anti-inflammatory balance over time in patients with septic shock and allow more detailed study of the other potential mechanisms of action of landiolol. Cortisol assays will measure the influence of beta blockade on the adrenal cortex.
- In addition, samples will be stored for subsequent analysis (eg, genetics/proteomics/metabolomics) in order to investigate early cellular responses during the resolution of sepsis.

### Safety outcomes

- The episodes of bradycardia (heart rate <50 bpm), bradycardia with haemodynamic compromise requiring intervention, significant hypotension requiring intervention (not including temporarily stopping the infusion), heart block, arrhythmia and arrhythmia with haemodynamic compromise requiring intervention will be reported.

### Sample size

**Figure 3:**
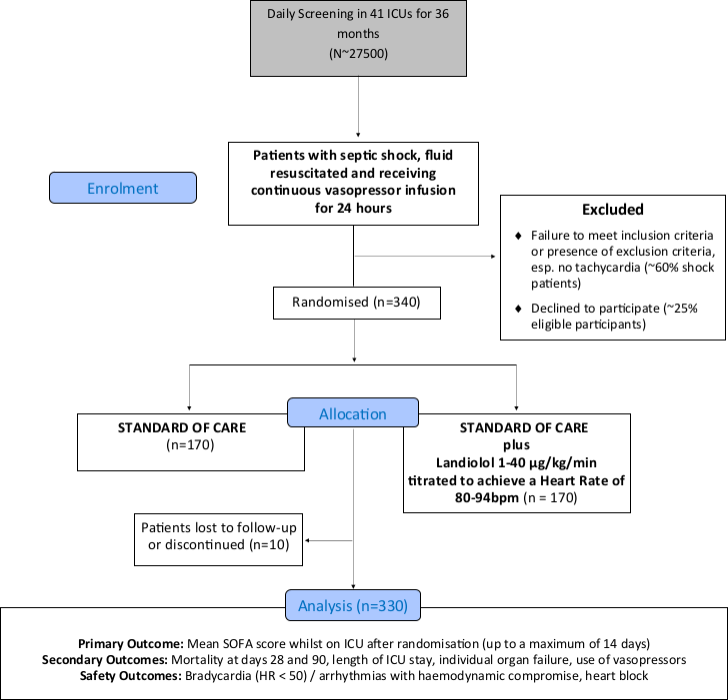
STRESS-L CONSORT flow diagram. bpm, beats per minute; CONSORT, Consolidated Standards of Reporting Trials; HR, heart rate; ICU, intensive care unit; SOFA, Sequential Organ Failure Assessment.

The primary outcome is the mean SOFA score over the first 14 days in ICU. Using preliminary collected data from 324 patients from UHB satisfying the trial eligibility criteria, the mean SOFA score over the first 14 days in ICU was 6.3, with SD 2.4. Assuming (conservatively) an SD of 2.8, and a difference of 1 point between the beta blocker and usual care groups, obtaining a p value less than 0.05 (two-sided) with 90% power would require outcome data on 330 patients. To allow for 3% withdrawals and losses, the proposed sample size is 340 (figure 3: STRESS-L CONSORT flow diagram).

### Assignment of interventions

#### Randomisation

We will use a computerised minimisation randomisation system, created by Warwick Clinical Trials Unit in accordance with their standard operating procedure and held on a secure server, with the allocation generated per individual (participant). Participants will be randomised strictly sequentially as they become eligible for randomisation using a 1:1 ratio. The randomisation will be stratified by recruiting site and norepinephrine dose where the dose reflects the participant’s severity. The dose will be dichotomised using a value of 0.3 µg/kg/min (ie, ≥0.1–0.3 µg/kg/min and >0.3 µg/kg/min) taken from the LeoPARDS trial as an indicator of low and high severity to ensure participant severity is balanced across both arms.^28^

Once written informed consent has been obtained, a member of the local research team will use an interactive voice response application to randomise. Eligibility will be confirmed by an investigator prior to randomisation. Sites will only be given access to the application once they have been given the ‘green light’ to begin recruitment and all required approvals are in place.

#### Blinding

This an open label trial with no blinding of the treatment allocation. Blinding is not possible due to the requirement for the landiolol dose to be titrated to achieve a target heart rate.

## Data collection, management and analysis

### Data collection

#### Screening

All patients with septic shock started on norepinephrine in ICU will be screened for trial eligibility. Once eligibility criteria are met there is a 48-hour window for randomisation. Due to this short window, informed consent may be sought during the first 24 hours of norepinephrine therapy. This will allow more time if legal representative consent is required. Randomisation will not occur until vasopressor therapy has been running for ≥24 hours, the patient is been treated with norepinephrine at rate >0.1 µg/kg/min and has a tachycardia with a heart rate ≥95. It will be made clear to the patient or their legal representative that if they no longer meet the eligibility criteria after the 48-hour mark (ie, 72 hours after the start of vasopressor therapy), the participant will not be randomised.

#### Baseline visit 24 hours prior and up to randomisation

Table 1 summarises the schedule of events for the trial. The patient’s medical history, physical examination, basic demographic data and steroid use will be recorded. The participant will be randomised, and if allocated to the landiolol arm, IMP will be dispensed and the infusion started. As there is no checking and preparation of drug in the usual care arm, the landiolol infusion should be started within 1 hour of randomisation. The elements of the SOFA score will be recorded along with routine clinical data (cardiovascular, respiratory and renal physiological variables as well as haematological, biochemical and microbiological sample test results). The rates of norepinephrine infusion will be recorded hourly. The site and types of pathogens isolated following admission should be recorded on the electronic CRF (eCRF).

**Table 1:** STRESS-L schedule of interventions

Procedure (time (T) in days/hours)	Screening (T0–12)	Baseline (day 0–24 hours—T0)	Day 1 (T0+24)	Day 2	Day 3	Day 4	Day 5	Day 6	Day 7	Day 8	Day 9	Day 10	Day 11	Day 12	Day 13	Day 14	EONT visit	FU visit day 28	Final visit day 90
Eligibility assessment	●																		
Informed consent		●																	
Randomisation		●																	
Demographics		●																	
Medical history		●																	
ECG		●	According to clinical need or if adverse event (AE)/serious AE (SAE)																
Pregnancy test		●																	
IMP		Dispense														End			
Blood sample		●	●	●		●		●									●		
Biobank blood sample (optional)		●	●														●		
Transport of stored serum																	Batch		
Local laboratory tests (normal clinical care):		●	●	●	●	●	●	●	●	●	●	●	●	●	●	●	●		
C reactive protein		●	●	●	●	●	●	●	●	●	●	●	●	●	●	●	●		
Glucose		●	●	●		●		●									●		
Lactate		●	●	●		●		●									●		
Worst PaO_2_/FiO_2_		●	●	●	●	●	●	●	●	●	●	●	●	●	●	●	●		
Platelets		●	●	●	●	●	●	●	●	●	●	●	●	●	●	●	●		
Creatinine		●	●	●	●	●	●	●	●	●	●	●	●	●	●	●	●		
Bilirubin		●	●	●	●	●	●	●	●	●	●	●	●	●	●	●	●		
White cell count		●	●	●	●	●	●	●	●	●	●	●	●	●	●	●	●		
Liver function tests (ALT or AST)		●	●	●		●		●									●		
Central venous blood gas (BG)/ arterial BG		●	●	●		●		●									●		
Microbiology results from local lab		●	●	●	●	●	●	●	●	●	●	●	●	●	●	●	●		
Heart rate (hourly: T0+7 days)		●	●	●	●	●	●	●	●	●	●	●	●	●	●	●	●		
Atrial fibrillation (hourly: T0+7 days)		●	●	●	●	●	●												
Blood pressure (hourly: T0+7 days)		●	●	●	●	●	●												
Rate of vasopressor/inotropes		●	●	●	●	●	●	●	●	●	●	●	●	●	●	●	●		
In/out fluids		●	●	●	●	●	●	●	●	●	●	●	●	●	●	●	●		
SOFA score		●	●	●	●	●	●	●	●	●	●	●	●	●	●	●	●		
AEs (SAEs up to day 90)		●	●	●	●	●	●	●	●	●	●	●	●	●	●	●	●	●	●
Steroid use		●	●	●		●		●									●		
Compliance		●	●	●	●	●	●	●	●	●	●	●	●	●	●	●	●		
Mortality status																		●	●
End of trial																			●

ALT, alanine aminotransferase; AST, aspartate aminotransferase; EONT, end of norepinephrine treatment; FiO_2_, fractional inspired oxygen; FU, follow-up; IMP, investigational medicinal product; PAO_2_, arterial oxygen tension; SOFA, Sequential Organ Failure Assessment.

#### Day 1 (time of randomisation to post-24 hours) up to day 14

The elements of the SOFA score will be recorded in the eCRF daily while the patient remains in the ICU from randomisation. This may be performed retrospectively but participating centres should ensure that blood has been sent to the local hospital laboratories for the domains that depend on laboratory tests (liver, renal and coagulation). If possible, blood should be taken for C-reactive protein. The rates of norepinephrine infusion and landiolol infusion (if randomised to this group) will be recorded hourly until day 2 to allow comparison of norepinephrine dosing and then 6 hourly thereafter. Heart rate data will be collected to allow assessment of landiolol infusion compliance and separation between groups.

Other clinical data collected will be haemodynamics, presence of atrial fibrillation, respiratory variables (blood gases with the worst P/F ratio, type of ventilator support and previous 24 hours’ intravenous fluid intake, urine output and need for renal replacement therapy (as given in table 1).

#### Follow-up visits (days 28 and 90)

Participants will also be followed up to ascertain survival status at 28 days and at 90 days post-randomisation. The participant’s community physician will be contacted in the first instance to ascertain if the patient is alive; the participant may then be contacted by telephone. Serious adverse events (SAEs) will be reported up to day 90 following randomisation.

#### Blood samples

Research blood samples will be collected on days 0, 1, 2, 4 and 6 and the EONT visit (if this does not fall on a blood sampling day). Day 0 blood samples must be taken prior to the start of landiolol infusion. Days 1, 2, 4, 6 and EONT blood samples can be taken when is convenient within the 24-hour time period. The plasma will be removed and stored as per detailed instructions provided in the trial laboratory manual. These research blood samples are mandatory as the results are required to answer the secondary outcomes of the trial.

### Statistical analysis

The primary analysis will be conducted according to intention to treat, comparing all those allocated to beta blocker (landiolol) with all of those allocated to usual care, regardless of treatment received. Regression models will be used to estimate the treatment effects (with 95% CIs), and the models will be adjusted for clinically important covariates. Subgroup effects for baseline severity measured using the dose of norepinephrine (norepinephrine ≥0.1–0.3 µg/kg/min vs >0.3 µg/kg/min) and use of beta blockers at baseline (Yes/No) will be assessed using formal statistical tests for interaction for the primary outcome and mortality. If there is substantial non-compliance, we will conduct (complier average causal effect) analyses to estimate the treatment effect among those who received the treatment as allocated. The DMC will meet every 6 months to closely monitor the accumulating data, focusing on safety. A detailed statistical analysis plan (SAP) will be written by the study statistical team, and then finalised and approved by the DMC before any analysis is undertaken.

### Missing data

Every effort will be made to minimise missing outcome data in this trial. Further exploratory analyses to assess the impact of any missing outcome data on the SOFA score will be examined using multiple imputation techniques and other methods specified in the SAP.

### Data monitoring

The Warwick Clinical Trials Unit will be responsible for trial monitoring and visits will be conducted in accordance with the monitoring plan. On-site monitoring visits during the trial will check the accuracy of data entered into the clinical trial database against the source documents, adherence to the protocol, procedures and Good Clinical Practice, and the progress of patient recruitment and follow-up.

## Safety, ethics and dissemination

### Adverse outcomes


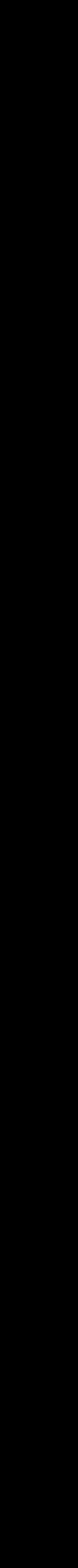
All SAEs and suspected unexpected SAEs occurring from the time of randomisation to the final follow-up visit at day 90 will be recorded on the CRF and faxed or emailed to the coordinating centre within 24 hours of the research staff becoming aware of the event. In particular, bradycardia (heart rate <50 bpm) with haemodynamic compromise requiring intervention, heart block, significant hypotension requiring intervention and arrhythmia with haemodynamic compromise requiring intervention should be recorded. AE/reactions will be assessed for seriousness and reported in accordance with Medicines and Healthcare products Regulatory Agency (MHRA) guidelines.

### Regulatory and ethics approvals

The East of England-Essex Research Ethics Committee (REC: 19/EE/0368—flagged for trials involving clinical trials in patients without capacity) and MHRA have approved the study protocol. The study will comply with the principles for sharing clinical trial data from publicly funded clinical trials.

### Dissemination

The study will be reported in accordance with the CONSORT guidelines.^24^ The study findings will be presented at national and international meetings with abstracts online. Presentation at these meetings will ensure that results and any implications quickly reach all of the UK and international intensive care communities. In accordance with the open access policies proposed by the National Institute for Health Research, we aim to publish the clinical findings of the trial in high-quality peer-reviewed open access (via PubMed) journals. Finally, an ongoing update of the trial will also be provided on the Warwick Clinical Trials Unit website and social media platforms, for example, Twitter (@STRESSL_trial).

### Confidentiality

In order to maintain confidentiality, all CRFs, questionnaires, study reports and communication regarding the study will identify the patients by the assigned unique trial identifier and initials only. Patient confidentiality will be maintained at every stage and will not be made publicly available to the extent permitted by the applicable laws and regulations.

### Patient and public involvement

This application is informed through a series of meetings with survivors and carers of patients who have had septic shock. Patient and public contributed to the trial proposal, protocol development and design of patient-facing materials. During the conduct of the trial, a patient and public involvement (PPI) member has been part of the Trial Management Group and has reviewed patient-facing documentation prior to the ethics and regulatory submissions and their comments have been incorporated. Two PPI representatives will sit on the Trial Steering Committee and will provide input from a patient perspective at trial meetings. Both representatives will review and provide feedback on all relevant project documents.

## Discussion

Many large multicentre trials of sepsis and septic shock have not derived statistical differences between study groups following single-centre pilot studies suggesting large mortality improvements.^29–31^ This is possibly because of the wide variation in severity of illness included in those studies. STRESS-L includes patients who are predicted to be at risk of a high mortality because they remain tachycardic (heart rate ≥95 bpm) after 24 hours of norepinephrine treatment. The persistence of tachycardia may phenotypically define a genetically different cohort as suggested in the population with acute respiratory distress syndrome.^32^ The trial uses landiolol because of its exceptional short half-life and highly specific beta-1 receptor blockade.

The primary outcome measure (mean SOFA score), which correlates with survival, allows measurement of the effect of landiolol on organ function compared with usual care. Analysis of each domain in the SOFA score will also begin to allow us to distinguish whether alteration in cardiac efficiency is the sole mechanism for differences between groups. The collection and analysis of blood samples will also characterise further the role of the adrenergic system in septic shock and propose genetic predisposition to this specific group of patients with septic shock.

STRESS-L recruited its first patient in April 2018 but a series of protocol amendments (owing to a complex intervention) and the COVID-19 pandemic have delayed the submission of this protocol paper. As of submission, there have been 109 patients recruited, approximately 32% of its target; no patients with COVID-19 have been recruited. Our SAP will address how patients with COVID-19 (if any) will be analysed. Unpublished data suggest that the inclusion criteria for STRESS-L are rarely achieved in primary COVID-19 illness; the presence of shock (defined by consensus^25^) coincidental with high-dose norepinephrine and a tachycardia, all within the time constraints, is seen in fewer than 1% of patients. Furthermore, our sites have had most of their research staff withdrawn to clinical duties so that screening and recruitment for the study has been suspended during period of exceptionally high admissions. We expect that the large majority of patients recruited to STRESS-L will be treated for non-COVID septic shock.

## Supplementary Material

Reviewer comments

Author's manuscript

## References

[1] Morelli A, Ertmer C, Westphal M, et al Effect of heart rate control with esmolol on hemodynamic and clinical outcomes in patients with septic shock: a randomized clinical trial. JAMA 2013;310:1683–91. 10.1001/jama.2013.278477 24108526

[2] Schwartz A, Brotfain E, Koyfman L, et al Cardiac arrhythmias in a septic ICU population: a review. J Crit Care Med 2015;1:140–6. 10.1515/jccm-2015-0027 PMC5953296 29967822

[3] Schmidt H, Müller-Werdan U, Hoffmann T, et al Autonomic dysfunction predicts mortality in patients with multiple organ dysfunction syndrome of different age groups. Crit Care Med 2005;33:1994–2002. 10.1097/01.CCM.0000178181.91250.99 16148471

[4] Beesley SJ, Wilson EL, Lanspa MJ, et al Relative bradycardia in patients with septic shock requiring vasopressor therapy. Crit Care Med 2017;45:225–33. 10.1097/CCM.0000000000002065 27618277 PMC5512273

[5] Dellinger RP, Levy MM, Rhodes A, et al Surviving sepsis campaign: international guidelines for management of severe sepsis and septic shock, 2012. Intensive Care Med 2013;39:165–228. 10.1007/s00134-012-2769-8 23361625 PMC7095153

[6] Hayes MA, Timmins AC, Yau EH, et al Elevation of systemic oxygen delivery in the treatment of critically ill patients. N Engl J Med 1994;330:1717–22. 10.1056/NEJM199406163302404 7993413

[7] Berk JL, Hagen JF, Beyer WH, et al The treatment of endotoxin shock by beta adrenergic blockade. Ann Surg 1969;169:74–81. 10.1097/00000658-196901000-00007 4883485 PMC1387267

[8] Suzuki T, Morisaki H, Serita R, et al Infusion of the beta-adrenergic blocker esmolol attenuates myocardial dysfunction in septic rats. Crit Care Med 2005;33:2294–301. 10.1097/01.CCM.0000182796.11329.3B 16215384

[9] Aboab J, Sebille V, Jourdain M, et al Effects of esmolol on systemic and pulmonary hemodynamics and on oxygenation in pigs with hypodynamic endotoxin shock. Intensive Care Med 2011;37:1344–51. 10.1007/s00134-011-2236-y 21567116

[10] Ackland GL, Yao ST, Rudiger A, et al Cardioprotection, attenuated systemic inflammation, and survival benefit of beta1-adrenoceptor blockade in severe sepsis in rats. Crit Care Med 2010;38:388–94. 10.1097/CCM.0b013e3181c03dfa 19829100

[11] Hagiwara S, Iwasaka H, Maeda H, et al Landiolol, an ultrashort-acting beta1-adrenoceptor antagonist, has protective effects in an LPS-induced systemic inflammation model. Shock 2009;31:515–20. 10.1097/SHK.0b013e3181863689 18791493

[12] Stolk RF, van der Pasch E, Naumann F, et al Norepinephrine dysregulates the immune response and compromises host defense during sepsis. Am J Respir Crit Care Med 2020;202:830–42. 10.1164/rccm.202002-0339OC 32520577

[13] Balik M, Rulisek J, Leden P, et al Concomitant use of beta-1 adrenoreceptor blocker and norepinephrine in patients with septic shock. Wien Klin Wochenschr 2012;124:552–6. 10.1007/s00508-012-0209-y 22815003

[14] Sanfilippo F, Santonocito C, Morelli A, et al Beta-blocker use in severe sepsis and septic shock: a systematic review. Curr Med Res Opin 2015;31:1817–25. 10.1185/03007995.2015.1062357 26121122

[15] de Montmollin E, Aboab J, Mansart A, et al Bench-to-bedside review: beta-adrenergic modulation in sepsis. Crit Care 2009;13:230. 10.1186/cc8026 19863760 PMC2784350

[16] Atarashi H, Kuruma A, Yashima M, et al Pharmacokinetics of landiolol hydrochloride, a new ultra-short-acting beta-blocker, in patients with cardiac arrhythmias. Clin Pharmacol Ther 2000;68:143–50. 10.1067/mcp.2000.108733 10976545

[17] Iguchi S, Iwamura H, Nishizaki M, et al Development of a highly cardioselective ultra short-acting beta-blocker, ONO-1101. Chem Pharm Bull 1992;40:1462–9. 10.1248/cpb.40.1462 1356643

[18] Takahata T, Yasui-Furukori N, Sakamoto J, et al Influence of hepatic impairment on the pharmacokinetics and pharmacodynamics of landiolol hydrochloride, an ultra-short-acting beta1-blocker. Drugs R D 2005;6:385–94. 10.2165/00126839-200506060-00006 16274261

[19] Seki Y, Jesmin S, Shimojo N Significant reversal of cardiac upregulated endothelin-1 system in a rat model of sepsis by landiolol hydrochloride. Life Sci 2014. 10.1016/j.lfs.2014.04.005 24735957

[20] Yoshida Y, Hongo T, Sakamoto A, et al Successful management of tachycardiac atrial fibrillation in a septic patient with landiolol. Anesth Analg 2005;100:294. 10.1213/01.ANE.0000140814.28118.6F 15616100

[21] Arita Y, Segawa T, Yamamoto S, et al Landiolol is effective for the treatment of tachycardia-induced cardiogenic shock in patients during septic shock therapy. BMJ Case Rep 2017;2017:bcr-2017-222268. 10.1136/bcr-2017-222268 PMC5695360 29092973

[22] Kakihana Y, Nishida O, Taniguchi T, et al Efficacy and safety of landiolol, an ultra-short-acting β1-selective antagonist, for treatment of sepsis-related tachyarrhythmia (J-Land 3S): a multicentre, open-label, randomised controlled trial. Lancet Respir Med 2020;8:863–72. 10.1016/S2213-2600(20)30037-0 32243865

[23] Chan A-W, Tetzlaff JM, Gøtzsche PC, et al SPIRIT 2013 explanation and elaboration: guidance for protocols of clinical trials. BMJ 2013;346:e7586. 10.1136/bmj.e7586 23303884 PMC3541470

[24] Moher D, Hopewell S, Schulz KF, et al CONSORT 2010 explanation and elaboration: updated guidelines for reporting parallel group randomised trials. BMJ 2010;340:c869. 10.1136/bmj.c869 20332511 PMC2844943

[25] Singer M, Deutschman CS, Seymour CW, et al The third International consensus definitions for sepsis and septic shock (Sepsis-3). JAMA 2016;315:801–10. 10.1001/jama.2016.0287 26903338 PMC4968574

[26] Charpentier J, Luyt C-E, Fulla Y, et al Brain natriuretic peptide: a marker of myocardial dysfunction and prognosis during severe sepsis. Crit Care Med 2004;32:660–5. 10.1097/01.CCM.0000114827.93410.D8 15090944

[27] Post F, Weilemann LS, Messow C-M, et al B-type natriuretic peptide as a marker for sepsis-induced myocardial depression in intensive care patients. Crit Care Med 2008;36:3030–7. 10.1097/CCM.0b013e31818b9153 18824903

[28] Gordon AC, Perkins GD, Singer M, et al Levosimendan for the prevention of acute organ dysfunction in sepsis. N Engl J Med 2016;375:1638–48. 10.1056/NEJMoa1609409 27705084

[29] , Finfer S, Chittock D, et al, NICE-SUGAR Study Investigators for the Australian and New Zealand Intensive Care Society Clinical Trials Group and the Canadian Critical Care Trials Group Intensive versus conventional glucose control in critically ill patients with traumatic brain injury: long-term follow-up of a subgroup of patients from the NICE-SUGAR study. Intensive Care Med 2015;41:1037–47. 10.1007/s00134-015-3757-6 26088909

[30] Venkatesh B, Finfer S, Cohen J, et al Adjunctive glucocorticoid therapy in patients with septic shock. N Engl J Med Overseas Ed 2018;378:797–808. 10.1056/NEJMoa1705835 29347874

[31] Ranieri VM, Thompson BT, Barie PS, et al Drotrecogin alfa (activated) in adults with septic shock. N Engl J Med 2012;366:2055–64. 10.1056/NEJMoa1202290 22616830

[32] Calfee CS, Janz DR, Bernard GR Distinct molecular phenotypes of direct versus indirect ARDS in single and multi-center studies. Chest 2014. 10.1378/chest.14-2454 PMC4451708 26033126

