# Supplementary material for: Study into the reversal of septic shock with landiolol (beta blockade): STRESS-L Study protocol for a randomised trial
Source: BMJ Open. 2021 Feb 16;11(2):e043194. doi: 10.1136/bmjopen-2020-043194 (PMC7888319; doi:10.1136/bmjopen-2020-043194)
Supplement: Author's manuscript [file bmjopen-2020-043194.draft_revisions.pdf]

BMJ Open

BMJ Open is committed to open peer review. As part of this commitment we make the peer review history of every article we publish publicly available.

When an article is published we post the peer reviewers' comments and the authors' responses online. We also post the versions of the paper that were used during peer review. These are the versions that the peer review comments apply to.

The versions of the paper that follow are the versions that were submitted during the peer review process. They are not the versions of record or the final published versions. They should not be cited or distributed as the published version of this manuscript.

BMJ Open is an open access journal and the full, final, typeset and author-corrected version of record of the manuscript is available on our site with no access controls, subscription charges or pay-per-view fees (http://bmjopen.bmj.com).

If you have any questions on BMJ Open's open peer review process please email info.bmjopen@bmj.com

BMJ Open

Study into the Reversal of Septic Shock with landiolol (Beta Blockade): Study Protocol for the STRESS-L Randomised Trial

Journal:	*BMJ Open*
Manuscript ID	bmjopen-2020-043194
Article Type:	Protocol
Date Submitted by the Author:	28-Jul-2020

Complete List of Authors:	Lall, Ranjit; University of Warwick Warwick Clinical Trials Unit<br>Mistry, Dipesh; University of Warwick Warwick Clinical Trials Unit<br>Skilton, Emma; University of Warwick Warwick Clinical Trials Unit<br>Perkins, Gavin; University of Warwick Warwick Clinical Trials Unit; University Hospitals Birmingham NHS Foundation Trust<br>Gordon, Anthony; Imperial College London, Division of Anaesthetics, Pain Medicine & Intensive Care<br>Bion, Julian; University of Birmingham, Intensive Care Medicine<br>Young, Duncan; University of Oxford, Kadoorie Centre for Critical Care Research, Nuffield Division of Anaesthesia<br>McAuley, Daniel; Queen's University Belfast, Centre for Experimental Medicine<br>Singer, Mervyn; University College London, Bloomsbury Inst of Intensive Care Medicine<br>Lord, Janet; University of Birmingham, NIHR Surgical Reconstruction & Microbiology Research Centre<br>Gates, Simon; University of Birmingham, Cancer Research UK Clinical Trials Unit; University of Warwick, Clinical Trials Unit<br>Regan, Scott; University of Warwick Warwick Clinical Trials Unit<br>Whitehouse, Tony; University Hospitals Birmingham NHS Foundation Trust, Department of Critical Care and Anaesthesia; University of Birmingham, Institute of Inflammation and Aging

Keywords:	Adult intensive & critical care < INTENSIVE & CRITICAL CARE, MICROBIOLOGY, STATISTICS & RESEARCH METHODS, Clinical trials < THERAPEUTICS

SCHOLARONE™ Manuscripts

I, the Submitting Author has the right to grant and does grant on behalf of all authors of the Work (as defined in the below author licence), an exclusive licence and/or a non-exclusive licence for contributions from authors who are: i) UK Crown employees; ii) where BMJ has agreed a CC-BY licence shall apply, and/or iii) in accordance with the terms applicable for US Federal Government officers or employees acting as part of their official duties; on a worldwide, perpetual, irrevocable, royalty-free basis to BMJ Publishing Group Ltd ("BMJ") its licensees and where the relevant Journal is co-owned by BMJ to the co-owners of the Journal, to publish the Work in this journal and any other BMJ products and to exploit all rights, as set out in our licence.

The Submitting Author accepts and understands that any supply made under these terms is made by BMJ to the Submitting Author unless you are acting as an employee on behalf of your employer or a postgraduate student of an affiliated institution which is paying any applicable article publishing charge ("APC") for Open Access articles. Where the Submitting Author wishes to make the Work available on an Open Access basis (and intends to pay the relevant APC), the terms of reuse of such Open Access shall be governed by a Creative Commons licence – details of these licences and which Creative Commons licence will apply to this Work are set out in our licence referred to above.

Other than as permitted in any relevant BMJ Author's Self Archiving Policies, I confirm this Work has not been accepted for publication elsewhere, is not being considered for publication elsewhere and does not duplicate material already published. I confirm all authors consent to publication of this Work and authorise the granting of this licence.

For peer review only

Study into the Reversal of Septic Shock with landiolol (Beta Blockade): Study Protocol for the STRESS-L Randomised Trial

R Lall[1], E Skilton[1], D Mistry[1], GD Perkins[1,2], AC Gordon[3], J Bion[4], D Young[5], D McAuley[6,7], M Singer[8], J Lord[9], S Gates[1], S Regan[1], T Whitehouse[2,9]

1. Warwick Clinical Trials Unit, University of Warwick, Coventry, UK
2. Department of Critical Care, University Hospitals Birmingham NHS Foundation Trust, Birmingham, UK
3. Division of Anaesthetics, Pain Medicine & Intensive Care, Imperial College London, London, UK
4. Institute of Clinical Sciences, College of Medical and Dental Sciences, University of Birmingham, UK
5. Kadoorie Centre for Critical Care Research, Nuffield Division of Anaesthesia, University of Oxford, Oxford, UK
6. Regional Intensive Care Unit, Royal Victoria Hospital, Belfast Health and Social Care Trust, Belfast, UK
7. The Wellcome Wolfson Institute for Experimental Medicine, Queens University Belfast, Belfast, UK
8. Centre for Intensive Care Medicine, Department of Medicine and Wolfson Institute for Biomedical Research, University College, London, UK
9. Institute of Inflammation and Ageing, Birmingham University Research labs., Queen Elizabeth Hospital, Birmingham, UK
10. Cancer Clinical Trials Unit, University of Birmingham, Birmingham, UK

Correspondence to Dr Tony Whitehouse Tony.Whitehouse@uhb.nhs.uk

Word Count: 4290

Keywords: Septic Shock, tachycardia, landiolol, beta blockade, SOFA score

For peer review only

"STRESS-L" Trial Protocol Summary

Objective: To assess the efficacy, tolerability, safety and mechanisms of potential benefit of landiolol in patients with septic shock requiring >24 hours treatment with high-dose vasopressor agents.

Design: Open label, unblinded, multi-centre, randomised phase IIb controlled clinical trial.

Setting: Study recruitment started in April 2018 in UK NHS hospitals with adult intensive care units (ICUs) that routinely manage patients with septic shock and where medical staff have clinical equipoise for the use of the protocolised landiolol infusion and agree to maintain randomised trial allocation.

Inclusion criteria: Patients aged ≥ 18 years old treated in an ICU, with septic shock, a heart rate ≥ 95 bpm and receiving vasopressor support (with noradrenaline ≥0.1 mcg/kg/min) to maintain a target blood pressure for ≥ 24 hours.

Exclusion criteria: Tachycardia not caused by septic shock (due to anxiety, pain, agitation), other forms of vasodilatory shock, > 72 hours in septic shock following initiation of vasopressor therapy, having pre-existing severe cardiac dysfunction and/or pulmonary hypertension, acute severe bronchospasm, untreated second or third degree heart block, untreated phaeochromocytoma, Prinzmetal's angina, previous ischaemic stroke or transient ischaemic attack or untreated severe carotid stenosis, advanced liver disease, sensitivity to beta-blockers, pregnant, no written informed consent, terminally ill, has had an investigational medicinal product from another clinical trial within the last 30 days, noradrenaline therapy coming to an end/withdrawn, receiving extracorporeal membrane oxygenation (ECMO) treatment.

Interventions: Usual care with an intravenous infusion of landiolol starting at 1.0 mcg/kg/min and progressively increasing every 15 minutes at increments of 1.0 mcg/kg/min, to reach a target heart rate of 80-94 bpm. The control group receive usual care but do not receive any beta blockade and there is no placebo infusion.

Outcome measures: *Primary outcome:* Mean Sequential Organ Failure Assessment (SOFA) score during 14 days after randomisation. *Secondary outcomes:* mortality, ICU and hospital stay, reduction in dose and duration of vasopressor treatment (total daily administered doses) *Mechanistic outcomes:* myocardial injury, metabolic and immune function. *Safety outcomes:* bradycardia, heart block, arrhythmia, other adverse events (AEs).

Sequence generation: Patients are randomised in a ratio of 1:1 to either landiolol or usual care, using a computerised minimisation randomisation system, stratified by recruitment site and dose of noradrenaline (≥0.1 to 0.3 mcg/kg/min; >0.3 mcg/kg/min).

For peer review only

Blinding: Open label with no blinding of treatment allocation. Blinding is not possible because the landiolol dose must be titrated to achieve the target heart rate.

Trial Registration: ISRCTN: 12600919; EudraCT: 2017-001785-14

Funding: National Institute of Health Research Technology Assessment Programme Efficacy and Mechanistic Evaluation (HTA EME reference: 14/150/85).

For peer review only

Abstract

A single centre study in the literature reported the safe use of esmolol in patients with septic shock and tachycardia who required vasopressor therapy for >24 hours. Although not powered to detect a change in mortality, marked improvements were seen in survival (adjusted hazard ratio, 0.39; 95% CI, 0.26-0.59; p <0.001). Beta blockers are one of the most studied groups of drugs, but their effect in septic shock is poorly understood; proposed mechanisms include not only the modulation of cardiac function but also immunomodulation. STRESS-L is a randomised, open-label, clinical trial which aims to enrol 340 patients with septic shock as defined by Sepsis-3 Consensus Definition and a tachycardia (HR ≥ 95 bpm) after vasopressor treatment of at least 24 hours. Standard randomisation (1:1 ratio) allocates patients to receive usual care (according to international standards) versus usual care and a continuous landiolol infusion to reduce the heart rate between 80 and 94 bpm. The primary endpoint is the mean SOFA score over 14 days from entry into the trial and whilst in ICU. Results will inform current clinical practice guidelines.

For peer review only

Background

A randomised single centre study of 154 patients from Rome [1] reported the use of beta-adrenergic blockade using the short-acting agent esmolol in patients with septic shock and tachycardia who required vasopressor therapy for more than 24 hours. The dosing endpoint was a reduction in heart rate between 80 and 94 beats/min (bpm). Though this study was not powered to detect a change in outcome, marked improvements were seen in survival (adjusted hazard ratio, 0.39; 95% CI, 0.26-0.59; p <0.001). Improvements were also seen in the beta-blocker-treated group in the duration of vasopressor treatment, and in measures of renal and cardiac function.

Autonomic dysfunction and tachycardia are associated with a poor outcome in septic shock [2] with mortalities in excess of 70% reported [3]. Bradycardia may be relatively protective [4]. Beta agonism is recommended as part of resuscitation in septic shock for restoration of cardiac output and blood pressure [5] despite an early study demonstrating that dobutamine used to increase cardiac index and systemic oxygen delivery was associated with an increased mortality [6]. Beta blockade represents a paradigm shift in management of patients with septic shock and arises from observations of harm induced by excessive sympathetic activation and benefit from beta-adrenergic blockade. Limited data from animal models [7-11] and patients [1 12] suggest their use is associated with improved cardiovascular and immune function. Excess beta-adrenergic activation in sepsis may be both endogenous (related to the severity and duration of the underlying infection) and exogenous due to the catecholamine therapy which has until now been the mainstay of management of hypotension caused by septic shock [5]. A combination of beta2 agonism with beta1 blockade improves sepsis-induced immune, cardiovascular and coagulation dysfunction in established sepsis [13].

Landiolol (Rapibloc®, AOP Orphan Pharmaceuticals, Vienna, Austria) is an ultra-short acting beta blocker that has a half-life of 2.3 to 4 minutes [14] and is approximately 8 times more selective for the beta 1 receptor than esmolol [15]. Landiolol is metabolised by plasma pseudocholinesterase and liver carboxylesterse to inactive metabolites and its metabolism is unaffected by liver impairment [16]. A few preclinical studies have examined landiolol in sepsis. It decreased circulating levels of the cytokines, TNF-alpha, IL-6, and high mobility group box (HMGB)-1 and reduced histological lung damage in a rat endotoxin model [11]. Landiolol was also cardio-protective in septic rats by normalising the expression of cardiac vasoactive peptide endothelin-1 [17]. Landiolol has been used safely and successfully in a septic patient with atrial fibrillation [18] and in sepsis-induced tachycardia [19].

For peer review only

Landiolol rather than esmolol was selected for this clinical trial because of its superior beta-1 specificity and the expectation that switching the landiolol infusion off would allow a rapid return to the baseline cardiovascular if investigators deemed the intervention harmful. Data on the safety of landiolol infusion is being collected as part of the study.

Study Objectives

Primary Objective

The primary objective of this trial is to assess the efficacy of landiolol (beta-blockade) in patients with septic shock and tachycardia requiring prolonged (>24 hours continuous treatment) support with high-dose vasopressor agents.

Secondary Objectives

The secondary objectives of the trial assess safety and mechanisms and are:

- to determine whether infusion of landiolol improves mortality and length of hospital stay compared with current best clinical practice, in patients who have septic shock.
- to investigate the pathways that are altered by beta blockade in septic shock by examining the effects of landiolol on blood markers of inflammation, metabolism and cardiomyocyte damage.

Methods and design

This manuscript has been written in concordance with the SPIRIT guidelines [20]

Trial design

This is a multi-centre, randomised, controlled open label phase IIb trial comparing usual treatment with usual treatment plus landiolol infusion in a total of 340 patients. The trial is being conducted and managed by Warwick Clinical Trials Unit and sponsored by the University of Hospitals of Birmingham

For peer review only

NHS Foundation Trust. It is coordinated by a Trial Management Group and independent oversight is provided by the Trial Steering Committee and a Data Monitoring Committee.

The funding is provided by the National Institute for Health Research Efficacy and Mechanistic Evaluation (NIHR EME reference number: 14/150/85). The trial has been designed and will be reported in line with the CONSORT (Consolidated Standards of Reporting Trials) statement [21] and the conduct has been planned in full conformance with the principles of the Declaration of Helsinki and Good Clinical Practice.

Study setting

The study recruitment started in April 2018. The main trial is taking place in approximately 41 UK adult intensive care units (ICUs). Participating ICUs which are recruiting to the study have a typical case mix for UK critical care, a track record of recruitment to clinical trials, suitable support for screening and data collection and the means to store blood samples for transfer to the central analysis unit. These units also manage atrial fibrillation with correction of potassium, magnesium and amiodarone in the usual care group as the use of beta-blockers in these patients would risk making the trial results uninterpretable.

Inclusion criteria

Patients are eligible to be included in the trial if they meet the following criteria:

- Aged 18 years or above
- Being treated in an ICU
- Septic shock according to internationally accepted definitions*
- Heart rate ≥95 bpm (at the time of randomisation)
- Receiving vasopressor support to maintain a target blood pressure for ≥24 hours
- Are being treated with noradrenaline at a rate ≥ 0.1mcg/kg/min

**Sepsis -3 definitions [22]:*

  - confirmed or suspected infection requiring antibiotic therapy
  - new organ dysfunction, as evidenced by an increase in SOFA[23] score ≥2
  - a blood lactate >2 mmol/l at any point during shock resuscitation
  - vasopressor therapy to maintain mean arterial pressure (MAP) ≥65 mmHg

For peer review only

In particular, the presence of a blood lactate > 2 mmol/l is only necessary for the diagnosis of septic shock and is NOT necessary for randomisation 24 hours later.

Exclusion criteria

A participant is not eligible for the study if any of the following apply:

- Tachycardia as a result of pain, discomfort from medical devices (including endotracheal tubes), during interventions or other patient distress
- Any form of vasodilatory shock that is not caused by sepsis
- Noradrenaline infusion <0.1mcg/kg/min
- >72 hours after start of vasopressor therapy
- <12 hours since noradrenaline to treat a medical condition other than septic shock stopped
- Having pre-existing severe cardiac dysfunction (NYHA grade 4 or more)
- Having pre-existing severe pulmonary hypertension (mean PA pressures > 55mmHg)
- Acute severe bronchospasm (due to asthma or COPD)
- Untreated second or third degree heart block
- Untreated phaeochromocytoma
- Prinzmetal's angina
- A past history of ischaemic stroke or transient ischaemic attack (TIA) or untreated severe carotid stenosis.
- Advanced liver disease with Child-Pugh Score of ≥B.
- Known sensitivity to beta-blockers
- Patient / legal representative unwilling to provide written informed consent
- Known to be pregnant
- Terminal illness other than septic shock with a life expectancy < 28 days
- Participants who have been administered an investigational medicinal product for another research trial in the past 30 days
- Patients in whom the clinical team feel are about to finish their noradrenaline therapy
- Decision of withdrawal of care is in place or imminently anticipated
- Receiving extracorporeal membrane oxygenation (ECMO) treatment.

Co-enrolment of study participants into other interventional studies has been considered where there is no biological interaction or impact on the STRESS-L trial end-points.

For peer review only

Consent

Eligible patients who are deemed competent are provided with a copy of the patient information sheet before informed consent is sought. However, due to the nature of the underlying condition and its treatment, many patients are unable to give informed consent. The vulnerability of this patient group is fully appreciated and every effort is made to protect their safety and well-being. To ensure this, the consent process is conducted in accordance with the Medicines for Human Use Regulations and the Health Research Authority ethics guide on medical research involving adults who cannot consent (http://www.hra.nhs.uk/resources/before-you-apply/consent-and-participation/adults-unable-to-consent-for-themselves/).

Intervention

Participants are randomised to either usual care (control group) or usual care and landiolol (intervention group).

Usual Care

Participants randomised to this arm receive usual care for septic shock, namely

- Timely treatment of the source of sepsis (e.g. drainage of infected fluid collections);
- Prompt and appropriate empiric antibiotic treatment, and modification, if needed, on the basis of culture results;
- Appropriate fluid resuscitation to correct hypovolaemia.

The patient is not to be randomised into the study until the treating physician is confident that adequate fluid resuscitation has been achieved and the patient has reached the target mean arterial pressure by the treating clinician overseeing care (suggested target 65-70 mmHg) using vasopressors. Adequate fluid resuscitation is achieved using repeated fluid challenges to a guided target; the use of a cardiac output monitor is not mandated. All other general ICU management is based on the latest guidance from the surviving sepsis campaign [5] and UK national critical care guidelines (e.g. ventilator and central line care bundles).

The usual care group does not receive any beta blockade for the duration of their ICU stay - if the treating clinician deems beta blockade necessary, this is captured on the Case Report Form and reported as a protocol deviation.

For peer review only

Usual care and landiolol

An intravenous infusion of landiolol is started at 1.0 mcg/kg/min and is progressively increased every 15 minutes at increments of 1.0 mcg/kg/min, to reach the target heart rate of 80-94 bpm usually within 6 hours. Landiolol is administered peripherally or centrally via a dedicated line. Landiolol has an elimination half-life of 2.3 to 4 minutes [15] and so a loading dose is unnecessary. The landiolol infusion is continued until the pulse rate is persistently below 95 bpm.

While a patient is receiving vasopressor agents (noradrenaline, vasopressin), the landiolol infusion is adjusted accordingly to maintain the target heart rate of 80-94 bpm as per the landiolol infusion protocol (Figure 2: STRESS-L Study Drug Infusion Protocol) and landiolol infusion and noradrenaline protocol (Figure 3: Timing and Weaning of the Study Drug). Once the patient is consistently within the target heart rate of 80-94 bpm, the landiolol Infusion is continued and not adjusted. If all vasopressor agents have been discontinued for less than 12 hours, the landiolol infusion will continue as per Figure 3.

The landiolol should be weaned and, if necessary, stopped whilst the heart rate (HR) is below 80 bpm. This trial allows for up to 14 days of landiolol treatment per participant.

The landiolol should be actively weaned once noradrenaline treatment has ceased for at least 12 hours (End of Noradrenaline Treatment [EONT])

Outcome measures

Primary outcome

The primary outcome is the mean SOFA score over the first 14 days from entry into the trial and whilst in ICU. Measurement of the SOFA score ceases if the patient withdraws from the trial, dies or is discharged from the ICU.

Secondary outcomes

- Mortality at day 28 and day 90 after randomisation
- Length of ICU and hospital stay
- Reduction in average daily administered dose and duration of vasopressor treatment

For peer review only

Mechanistic outcomes

Serial blood samples are collected from patients and assays include markers of myocardial dysfunction and inflammation.

- Measurement of total serum catecholamine concentration. It is unknown whether beta-blockade acts through altering the effects of extraneous catecholamines administered as treatment of septic shock or by modulating those produced by the patient. Serum catecholamines will be analysed in the context of the dose of inotropes being administered.
- Markers of myocardial dysfunction. Serum BNP has been demonstrated to be a reliable biomarker of myocardial injury, ischaemia and dysfunction in septic patients and also as a prognostic marker for a poor outcome [24 25]. Serial measurements of troponin-T will be made.
- Measurement of serum free fatty acids and markers of fatty acid metabolism.
- Biomarkers of systemic inflammation. This will be measured using a multiplex inflammatory biomarker assay depending on the available technology at the time of analysis. A selection of cytokines will be analysed, all or some of which will include IL-1beta, IL-2, IL-4, IL-5, IL-6, IL-8, IL-10, IL-12, TNF-alpha, TNF-beta, and IFN-gamma. This focused array will allow an assessment of the pro / anti-inflammatory balance over time in patients with septic shock and allow more detailed study of the other potential mechanisms of action of landiolol. Cortisol assays will measure the influence of beta blockade on the adrenal cortex.
- In addition, samples will be stored for subsequent analysis (e.g. genetics / proteomics / metabolomics) in order to investigate early cellular responses during the resolution of sepsis.

Safety outcomes

- The episodes of bradycardia (HR <50 bpm), bradycardia with haemodynamic compromise requiring intervention, significant hypotension requiring intervention (not including temporarily stopping the infusion), heart block, arrhythmia, arrhythmia with haemodynamic compromise requiring intervention will be reported.

For peer review only

Sample size

The primary outcome is the mean SOFA score over the first 14 days in ICU. Using preliminary data collected from 324 patients treated in the University Hospital Birmingham ICU satisfying the trial eligibility criteria, the mean SOFA score over the first 14 days in ICU was 6.3, with standard deviation 2.4. Assuming (conservatively) a standard deviation of 2.8, and a clinically relevant difference of 1 between the beta blocker and usual care groups, obtaining a p-value less than 0.05 (two-sided) with 90% power would require outcome data on 330 patients. To allow for 3% withdrawals and losses, the proposed sample size is 340 (Figure 1).

Assignment of Interventions

Randomisation

We are using a computerised minimisation randomisation system, created by Warwick Clinical Trials Unit in accordance with their standard operating procedure and held on a secure server, with the allocation generated per individual (participant). Participants are randomised strictly sequentially as they become eligible for randomisation using a 1:1 ratio. The randomisation is minimised by recruiting site and noradrenaline dose. The dose is dichotomised using a value of 0.3 mcg/kg/min (i.e. ≥0.1 mcg/kg/min to 0.3 mcg/kg/min and >0.3 mcg/kg/min) taken from the LeoPARDS trial as an indicator of low and high severity to ensure shock severity is balanced across both arms [26].

Once written informed consent has been obtained, the local research team use an interactive voice response (IVR) application to randomise. Due to the nature of this population an out of hours randomisation service is required. Eligibility is confirmed by an investigator prior to randomisation. Sites are only given access to the application once they have been given the 'green light' to begin recruitment, all required approvals have been put in place and drug is available on the ICU. The IVR application captures all the essential data required to randomise a participant and then provides the researcher with the unique participant trial number and allocation.

For peer review only

Blinding

This is an open label trial with no blinding of the treatment allocation. Blinding is not possible due to the requirement for the landiolol dose to be titrated to achieve a target heart rate.

Data collection, management and analysis

Data Collection

Screening

All patients with septic shock who start on noradrenaline in ICU are screened for trial eligibility. Randomisation does not occur until vasopressor therapy has been running for ≥24 hours. At randomisation, the patient is being treated with noradrenaline at rate >0.1 mcg/kg/min and is tachycardic (HR≥95). Once eligibility criteria are met there is a 48-hour window for randomisation. Due to this narrow window, informed consent can be sought during the first 24 hours of vasopressor therapy. This allows more time if legal representative consent is required. Patients or their legal representatives are informed if the participant cannot be randomised due to them no longer meeting the eligibility criteria after the 48-hour mark (i.e. 72 hours after the start of vasopressor therapy).

Baseline visit 24 hours prior and up to randomisation

Informed consent is obtained at (or leading up to) this visit and a final check against eligibility criteria is undertaken. The patient's medical history, physical examination, basic demographic data and steroid use are recorded. The participant is randomised, and if allocated to the landiolol arm, the IMP is dispensed and the infusion started. For patients randomised to the usual care arm, data collection is started immediately as there is no drug check or preparation required. In order to maintain parity between arms, the landiolol infusion is usually started within 1 hour of randomisation. Any AEs are recorded, and any Serious Adverse Events (SAEs) are reported following randomisation for all patients in the study. The elements of the SOFA score are recorded along with routine clinical data (cardiovascular, respiratory and renal physiological variables as well as haematological, biochemical and microbiological sample test results). The rates of noradrenaline infusion are recorded hourly. The site and types of pathogens isolated in any clinical sample following admission are recorded on the electronic case record form.

For peer review only

Day 1 (time of randomisation to post 24 hours) up to day 14

The elements of the SOFA score are recorded in the case record form daily from randomisation whilst the patient remains in the ICU. This may be performed retrospectively but participating centres should ensure that blood samples are being sent to the local hospital laboratories for the SOFA domains that depend on laboratory tests (liver, renal and coagulation) and C-reactive protein. In addition, routinely collected clinical data (cardiovascular, respiratory and renal physiological variables as well as haematological, biochemical and microbiological sample test results) are recorded whilst noradrenaline is being infused. The rates of noradrenaline infusion and landiolol infusion (if randomised to this group) are recorded hourly until day 2 to allow comparison of noradrenaline dosing and then 6-hourly thereafter. Heart rate data are collected to allow assessment of landiolol infusion compliance and separation between groups.

Other clinical data collected include haemodynamics, respiratory variables and previous 24 hours' intravenous fluid intake, urine output, and need for renal replacement therapy (as given in Table 1).

Follow up visits (Days 28 and 90)

Participants are also followed up to ascertain survival status at 28 days and at 90 days post-randomisation. The participant's family doctor (General Practitioner) will be contacted in the first instance to ascertain if the patient is alive; the participant may then be contacted by telephone. SAEs will be reported up to day 90 following randomisation.

Blood samples

Research blood samples are collected on Days 0, 1, 2, 4 and 6 and the EONT visit (if this does not fall on a blood sampling day). Day 0 blood samples must be taken as soon as possible after randomisation and prior to the start of landiolol infusion. Days 1, 2, 4, 6 and EONT blood samples are taken when the local site is able to process the sample at any point within the calendar day. The plasma is removed and stored as per detailed instructions provided in the trial Laboratory Manual. These research blood samples are mandatory as the results are required to answer the secondary outcomes of the trial.

For peer review only

Statistical Analysis

The primary analysis will be conducted according to intention to treat, comparing all those allocated to beta blocker (landiolol) with all of those allocated to usual care, regardless of treatment received. Regression models will be used to estimate the treatment effects (with 95% confidence intervals), and the models will be adjusted for clinically important covariates. Subgroup effects for baseline severity measured using the dose of noradrenaline (noradrenaline ≥0.1 mcg/kg/min - 0.3 mcg/kg/min vs. >0.3 mcg/kg/min) and use of beta blockers at baseline (Yes/No) will be assessed using formal statistical tests for interaction for the primary outcome and mortality. If there is substantial non-compliance, we will conduct Complier Average Causal Effect analyses[26] to estimate the treatment effect among those who received the treatment as allocated. The Data Monitoring Committee will meet every 6 months to closely monitor the accumulating data, focusing on safety. A detailed statistical analysis plan will be written by the study statistical team and then finalised and approved by the Data Monitoring Committee before any analysis is undertaken.

Missing data

Every effort is made to minimise missing outcome data in this trial. Further exploratory analyses to assess the impact of any missing outcome data on the SOFA-score will be examined using multiple imputation techniques and other methods specified in the statistical analysis plan.

Data Monitoring

The Warwick Clinical Trials Unit is responsible for trial monitoring and visits are conducted in accordance with the monitoring plan. On-site monitoring visits during the trial have been initiated to check the accuracy of data entered into the clinical trial database against the source documents, adherence to the protocol, procedures and Good Clinical Practice and the progress of patient recruitment and follow-up.

Patient and public involvement

This application has been developed in partnership with survivors and carers of patients who have had septic shock. Patients and members of the public contributed to the trial proposal, protocol

For peer review only - http://bmjopen.bmj.com/site/about/guidelines.xhtml

development and design of patient facing materials. During the conduct of the trial a Patient and Public Involvement (PPI) member has been part of the Trial Management Group and has reviewed patient facing documentation prior to the ethics and regulatory submissions and their comments have been incorporated. Two PPI representatives will sit on the Trial Steering Committee and will provide input from a patient perspective at trial meetings. Both representatives will review and provide feedback on all relevant project documents.

Safety, ethics and dissemination

Adverse outcomes

All suspected and unexpected SAEs occurring from the time of randomisation to the final follow up visit at day 90 are recorded on the Case Record Form and faxed or emailed to the coordinating centre within 24 hours of the research staff becoming aware of the event. In particular, bradycardia (HR <50 bpm) with haemodynamic compromise requiring intervention, heart block, significant hypotension requiring intervention, arrhythmia with haemodynamic compromise requiring intervention are recorded. AEs and reactions are assessed for seriousness and reported in accordance with MHRA guidelines.

Regulatory and ethics approvals

The East of England - Essex Research Ethics Committee (REC: 19/EE/0368 - flagged for trials involving clinical trials in patients without capacity) and Medical Healthcare Regulatory Authority (MHRA) (EudraCT 2017-001785-14) have approved the study protocol. The ICRSTN trial registration number is ISRCTN12600919. The study will comply with the principles for sharing clinical trial data from publicly funded clinical trials.

Confidentiality

In order to maintain confidentiality when amalgamating data between sites, all case report forms, questionnaires, study reports and communication will identify the patients by the assigned unique

For peer review only

trial identifier and initials only. Patient confidentiality will be maintained at every stage and will not be made publicly available to the extent permitted by the applicable laws and regulations.

Dissemination

The study will be reported in accordance with the Consolidated Standards of Reporting Trials (CONSORT) guidelines [21]. The study findings will be presented at national and international meetings with abstracts on-line. Presentation at these meetings will ensure that results and any implications quickly reach all of the UK and international intensive care community. In accordance with the open access policies proposed by the NIHR, we aim to publish the clinical findings of the trial in high quality peer-reviewed open access (via Pubmed) journals. Finally, an on-going update of the trial will also be provided on the Warwick Clinical Trials Unit website, its own website (www.stress-l.org) and social media platforms e.g. Twitter@STRESSL trial).

Discussion

Heterogeneity of case mix and severity of illness complicates research in critical illness despite well-validated methods of adjustment, and many trials in sepsis have either resulted in inconclusive or negative outcomes. In STRESS-L we have chosen to focus on a sub-group of patients with a high risk of death from septic shock who remain tachycardic (HR >= 95 bpm) after 24 hours of noradrenaline treatment, a relatively homogenous group and one in which an earlier mechanistic study of beta-blockade suggested a survival advantage. The trial uses landiolol because its exceptional short half-life and highly specific beta1 receptor blockade offer additional safety.

The primary outcome measure (mean SOFA score), which correlates with survival, allows measurement of the effect of landiolol on organ function compared with usual care. Analysis of each domain in the SOFA score will also begin to allow us to distinguish whether alteration in cardiac efficiency is the sole mechanism for differences between groups. The persistence of tachycardia may phenotypically define a genetically different cohort and we will compare genotypes with existing libraries from other genetic studies of the critically ill. The collection and analysis of blood samples will also characterise further the role of the adrenergic system in septic shock and proposed genetic predisposition to this specific group of patients with septic shock.

For peer review only

Contributions

TW and JFB conceived the initial trial concept and helped develop the trial design and protocol. GDP, DFM, MS, ACG helped develop the trial design and protocol. RL, DM and SG carried out the power calculations and helped develop the trial design and protocol. TW is the chief investigator and takes overall responsibility for all aspects of trial design, the protocol and trial conduct. All authors read and approved the final manuscript.

Competing Interests

TW and MS have received travel expenses from AOP Orphan, manufacturer of landiolol, for delivering lectures. All other authors declare they have no competing interests.

Acknowledgements

The authors would like to thank AOP Orphan, Vienna, Austria, for providing the landiolol for the trial free of charge. ACG is the Deputy Director and GDP & DFM are Co-directors of Research for the Intensive Care Foundation. We are grateful for the infrastructure provided by the National Institute of Health Research (NIHR) Critical Care Specialty Group of the Comprehensive Clinical Research Network. We would like to express our gratitude to the members of the Trial Steering Committee and the Data Monitoring Committee who provide support and guidance for this study.

Funding

This project was funded by the Efficacy and Mechanism Evaluation (EME) Programme and managed by the NIHR. ACG is funded by a NIHR Clinician Scientist Fellowship. MS and GDP are supported by NIHR Senior Investigator Fellowships. The views expressed in this publication are those of the author(s) and not necessarily those of the EME, NHS, NIHR or the Department of Health.

For peer review only

References

1. Morelli A, Ertmer C, Westphal M, et al. Effect of heart rate control with esmolol on hemodynamic and clinical outcomes in patients with septic shock: a randomized clinical trial. *JAMA* 2013;310(16):1683-91. doi: 1752246 [pii];10.1001/jama.2013.278477 [doi]
2. Schwartz A, Brotfain E, Koyfman L, et al. Cardiac Arrhythmias in a Septic ICU Population: A Review. *J Crit Care Med (Targu Mures)* 2015;1(4):140-46. doi: 10.1515/jccm-2015-0027 [published Online First: 2015/11/10]
3. Schmidt H, Muller-Werdan U, Hoffmann T, et al. Autonomic dysfunction predicts mortality in patients with multiple organ dysfunction syndrome of different age groups. *Crit Care Med* 2005;33(9):1994-2002. doi: 10.1097/01.ccm.0000178181.91250.99 [published Online First: 2005/09/09]
4. Beesley SJ, Wilson EL, Lanspa MJ, et al. Relative Bradycardia in Patients With Septic Shock Requiring Vasopressor Therapy. *Crit Care Med* 2017;45(2):225-33. doi: 10.1097/CCM.0000000000002065 [published Online First: 2016/09/13]
5. Dellinger RP, Levy MM, Rhodes A, et al. Surviving Sepsis Campaign: international guidelines for management of severe sepsis and septic shock, 2012. *Intensive Care Med* 2013;39(2):165-228. doi: 10.1007/s00134-012-2769-8 [doi]
6. Hayes MA, Timmins AC, Yau EH, et al. Elevation of systemic oxygen delivery in the treatment of critically ill patients. *N Engl J Med* 1994;330(24):1717-22. doi: 10.1056/NEJM199406163302404 [doi]
7. Berk JL, Hagen JF, Beyer WH, et al. The treatment of endotoxin shock by beta adrenergic blockade. *Ann Surg* 1969;169(1):74-81.
8. Suzuki T, Morisaki H, Serita R, et al. Infusion of the beta-adrenergic blocker esmolol attenuates myocardial dysfunction in septic rats. *Crit Care Med* 2005;33(10):2294-301. doi: 00003246-200510000-00021 [pii]
9. Aboab J, Sebille V, Jourdain M, et al. Effects of esmolol on systemic and pulmonary hemodynamics and on oxygenation in pigs with hypodynamic endotoxin shock. *Intensive Care Med* 2011;37(8):1344-51. doi: 10.1007/s00134-011-2236-y [doi]
10. Ackland GL, Yao ST, Rudiger A, et al. Cardioprotection, attenuated systemic inflammation, and survival benefit of beta1-adrenoceptor blockade in severe sepsis in rats. *Crit Care Med* 2010;38(2):388-94. doi: 10.1097/CCM.0b013e3181c03dfa [doi]
11. Hagiwara S, Iwasaka H, Maeda H, et al. Landiolol, an ultrashort-acting beta1-adrenoceptor antagonist, has protective effects in an LPS-induced systemic inflammation model. *Shock* 2009;31(5):515-20. doi: 10.1097/SHK.0b013e3181863689 [doi]
12. Balik M, Rulisek J, Leden P, et al. Concomitant use of beta-1 adrenoreceptor blocker and norepinephrine in patients with septic shock. *Wien Klin Wochenschr* 2012;124(15-16):552-56. doi: 10.1007/s00508-012-0209-y [doi]
13. de ME, Aboab J, Mansart A, et al. Bench-to-bedside review: Beta-adrenergic modulation in sepsis. *Crit Care* 2009;13(5):230. doi: cc8026 [pii];10.1186/cc8026 [doi]
14. Atarashi H, Kuruma A, Yashima M, et al. Pharmacokinetics of landiolol hydrochloride, a new ultra-short-acting beta-blocker, in patients with cardiac arrhythmias. *Clin Pharmacol Ther* 2000;68(2):143-50. doi: S0009923600082473 [pii];10.1067/mcp.2000.108733 [doi]
15. Iguchi S, Iwamura H, Nishizaki M, et al. Development of a highly cardioselective ultra short-acting beta-blocker, ONO-1101. *Chem Pharm Bull (Tokyo)* 1992;40(6):1462-69.
16. Takahata T, Yasui-Furukori N, Sakamoto J, et al. Influence of hepatic impairment on the pharmacokinetics and pharmacodynamics of landiolol hydrochloride, an ultra-short-acting beta1-blocker. *Drugs R D* 2005;6(6):385-94. doi: 666 [pii]

For peer review only

17. Seki Y, Jesmin S, Shimojo N, et al. Significant reversal of cardiac upregulated endothelin-1 system in a rat model of sepsis by landiolol hydrochloride. *Life Sci* 2014 doi: S0024-3205(14)00405-6 [pii];10.1016/j.lfs.2014.04.005 [doi]
18. Yoshida Y, Hongo T, Sakamoto A, et al. Successful management of tachycardiac atrial fibrillation in a septic patient with landiolol. *Anesth Analg* 2005;100(1):294. doi: 100/1/294-a [pii];10.1213/01.ANE.0000140814.28118.6F [doi]
19. Arita Y, Segawa T, Yamamoto S, et al. Landiolol is effective for the treatment of tachycardia-induced cardiogenic shock in patients during septic shock therapy. *BMJ Case Reports* 2017;2017:bcr-2017-222268. doi: 10.1136/bcr-2017-222268
20. Chan AW, Tetzlaff JM, Gotzsche PC, et al. SPIRIT 2013 explanation and elaboration: guidance for protocols of clinical trials. *BMJ* 2013;346:e7586. doi: 10.1136/bmj.e7586 [published Online First: 2013/01/11]
21. Moher D, Hopewell S, Schulz KF, et al. CONSORT 2010 explanation and elaboration: updated guidelines for reporting parallel group randomised trials. *BMJ* 2010;340:c869. doi: 10.1136/bmj.c869 [published Online First: 2010/03/25]
22. Singer M, Deutschman CS, Seymour CW, et al. The Third International Consensus Definitions for Sepsis and Septic Shock (Sepsis-3). *JAMA* 2016;315(8):801-10. doi: 2492881 [pii];10.1001/jama.2016.0287 [doi]
23. Vincent JL, Moreno R, Takala J, et al. The SOFA (Sepsis-related Organ Failure Assessment) score to describe organ dysfunction/failure. On behalf of the Working Group on Sepsis-Related Problems of the European Society of Intensive Care Medicine. *Intensive Care Med* 1996;22(7):707-10.
24. Charpentier J, Luyt CE, Fulla Y, et al. Brain natriuretic peptide: A marker of myocardial dysfunction and prognosis during severe sepsis. *Crit Care Med* 2004;32(3):660-65. doi: 00003246-200403000-00007 [pii]
25. Post F, Weilemann LS, Messow CM, et al. B-type natriuretic peptide as a marker for sepsis-induced myocardial depression in intensive care patients. *Crit Care Med* 2008;36(11):3030-37. doi: 10.1097/CCM.0b013e31818b9153 [doi]
26. Gordon AC, Perkins GD, Singer M, et al. Levosimendan for the Prevention of Acute Organ Dysfunction in Sepsis. *N Engl J Med* 2016;375(17):1638-48. doi: 10.1056/NEJMoa1609409 [doi]

For peer review only

Trial CONSORT flow diagram

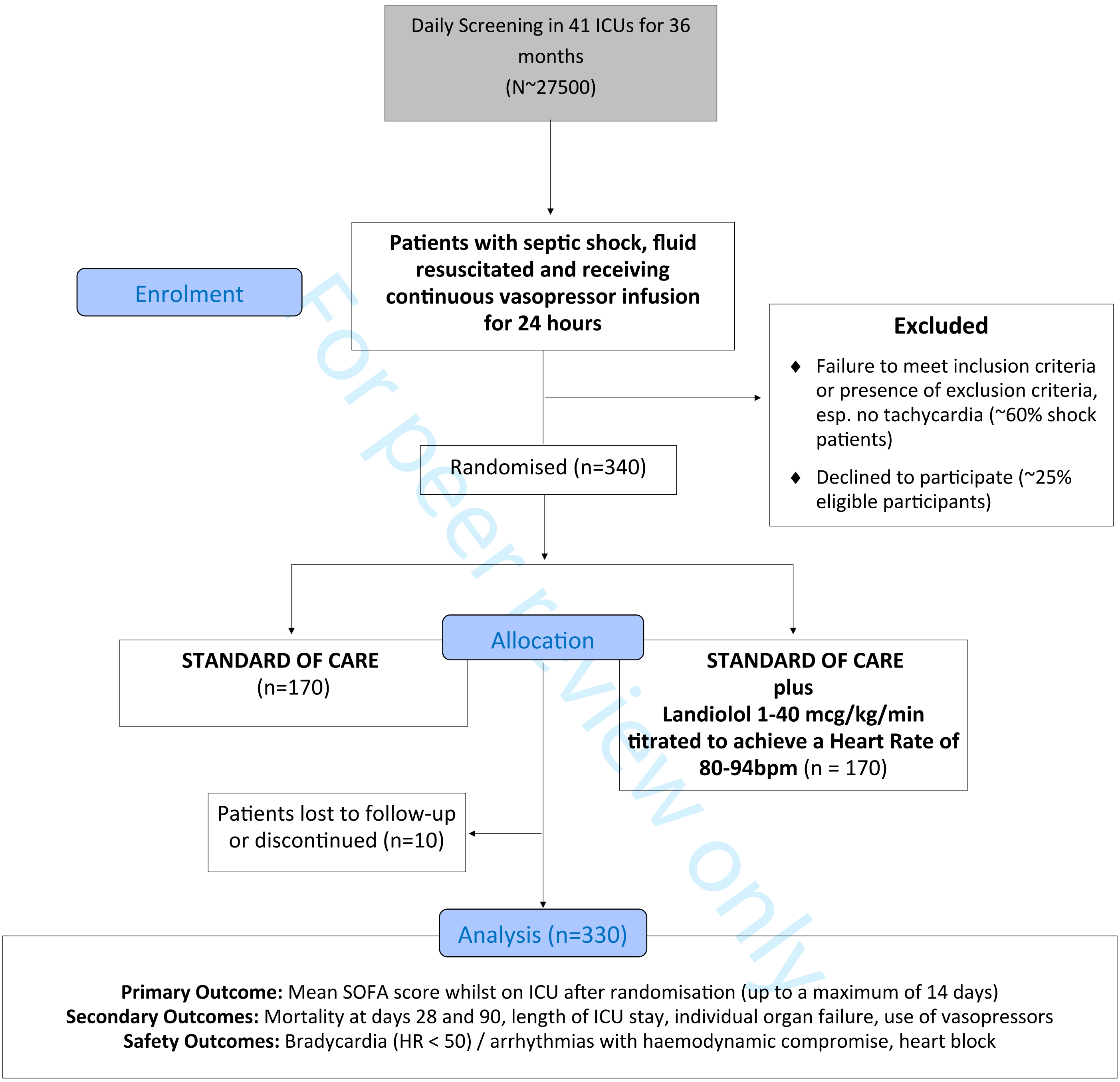

For peer review only

FIGURE 2: STRESS-L Study Drug Infusion Protocol

Landiolol is an ultra-short antagonist of the beta 1 adrenergic receptor

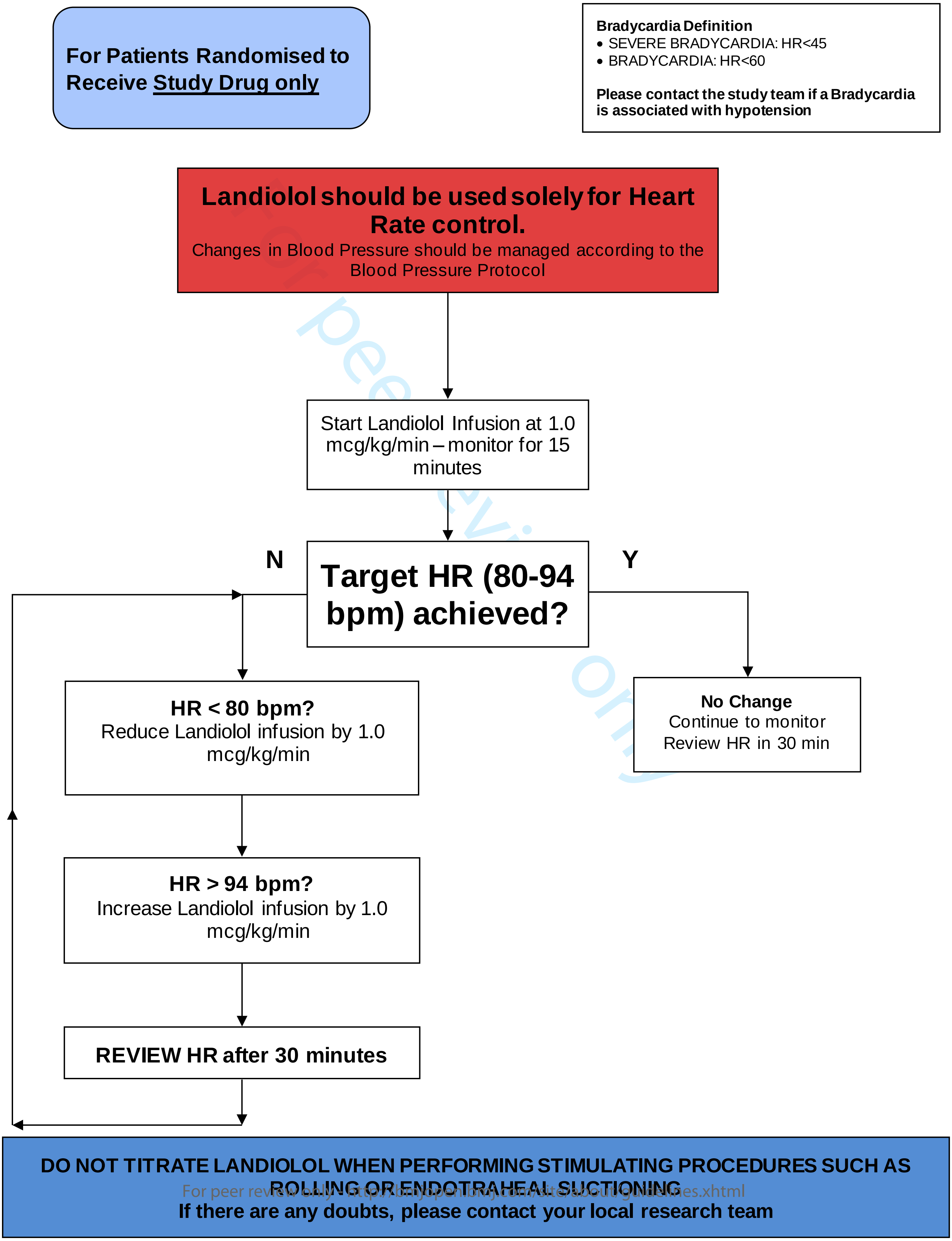

For peer review only - http://bmjopen.bmj.com/site/about/guidelines.xhtml

Figure 3 - STRESS-L Study Drug and Noradrenaline Infusion Adjustment

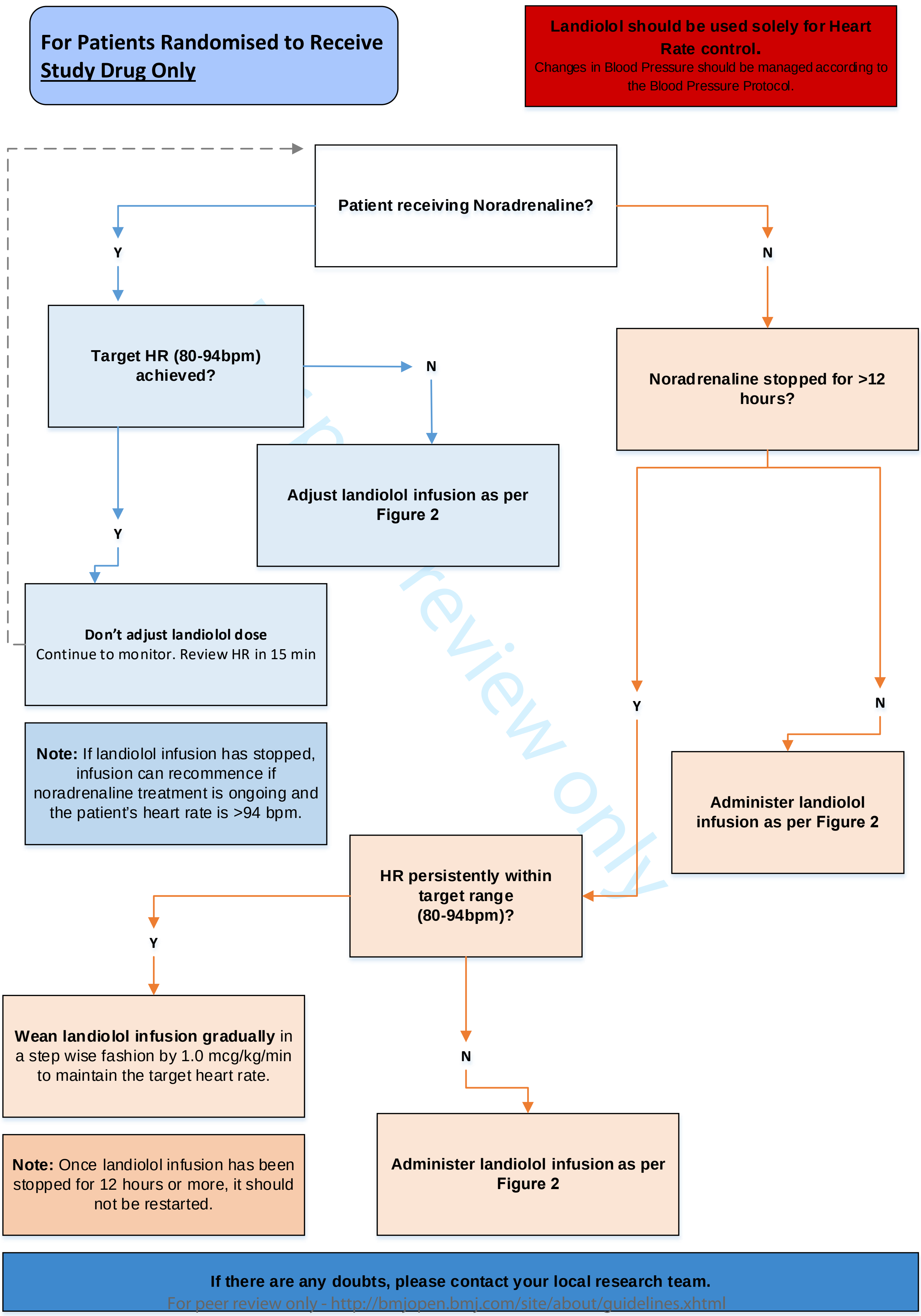

Table 1: Trial Assessments

Procedure (Time (T) in days/hours)	Screening (T0-24hr)	Baseline (T0)	Day 1 (T0+24hr)	Day 2	Day 3	Day 4	Day 5	Day 6	Day 7	Day 8	Day 9	Day 10	Day 11	Day 12	Day 13	Day 14	EONT[1] Visit	FU Visit Day 28	Final visit Day 90
Eligibility assessment	•																		
Informed consent		•																	
Randomisation		•																	
Demographics		•																	
Medical History		•																	
ECG		•	According to clinical need or if AE/SAE																
Pregnancy test[2]		•																	
IMP		Dispense														End			
Blood sample		•	•	•		•		•									•		
Biobank blood sample (optional)		•	•														•		
Transport of stored serum																	Batch		
Local laboratory tests (normal clinical care):		•	•	•	•	•	•	•	•	•	•	•	•	•	•	•	•		
C-Reactive Protein (CRP)		•	•	•	•	•	•	•	•	•	•	•	•	•	•	•	•		
Glucose		•	•	•		•		•									•		
Lactate		•	•	•		•		•									•		
Worst PaO2/FiO2		•	•	•	•	•	•	•	•	•	•	•	•	•	•	•	•		
Platelets		•	•	•	•	•	•	•	•	•	•	•	•	•	•	•	•		
Creatinine		•	•	•	•	•	•	•	•	•	•	•	•	•	•	•	•		
Bilirubin		•	•	•	•	•	•	•	•	•	•	•	•	•	•	•	•		
White Cell Count		•	•	•	•	•	•	•	•	•	•	•	•	•	•	•	•		
Liver Function Tests (ALT or AST)		•	•	•		•		•									•		
Central Venous Blood Gas / Arterial BG		•	•	•		•		•									•		
Microbiology results from local lab		•	•	•	•	•	•	•	•	•	•	•	•	•	•	•	•		
Heart Rate (Hourly: T0+7 days)		•	•	•	•	•	•	•	•	•	•	•	•	•	•	•	•		
Atrial Fibrillation (Hourly: T0+7 days)		•	•	•	•	•	•												
Blood Pressure (Hourly: T0+7 days)		•	•	•	•	•	•												
Rate of Vasopressor / inotropes		•	•	•	•	•	•	•	•	•	•	•	•	•	•	•	•		
In/Out Fluids		•	•	•	•	•	•	•	•	•	•	•	•	•	•	•	•		
SOFA score		•	•	•	•	•	•	•	•	•	•	•	•	•	•	•	•		
Adverse Events (SAEs up to Day 90)		•	•	•	•	•	•	•	•	•	•	•	•	•	•	•	•	•	•
Steroid use		•	•	•		•		•									•		
Compliance		•	•	•	•	•	•	•	•	•	•	•	•	•	•	•	•		
Mortality status																		•	•
End of Trial																			•

1. EONT = End of Noradrenaline Treatment: End of all vasopressor infusions +12 hours (may occur at any point between day 1 and day 14); see protocol section 3.8.5 for full details. The maximum duration of landiolol treatment is 14 days.
2. Pregnancy test on women of childbearing potential at the discretion of local investigator

For peer review only

Study into the Reversal of Septic Shock with Landiolol (Beta Blockade): STRESS-L. Study Protocol for a Randomised Trial

Journal:	*BMJ Open*
Manuscript ID	bmjopen-2020-043194.R1
Article Type:	Protocol
Date Submitted by the Author:	16-Dec-2020
Complete List of Authors:	Lall, Ranjit; University of Warwick Warwick Clinical Trials Unit<br>Mistry, Dipesh; University of Warwick Warwick Clinical Trials Unit<br>Gates, Simon; University of Birmingham, Cancer Research UK Clinical Trials Unit; University of Warwick, Clinical Trials Unit<br>Skilton, Emma; University of Warwick Warwick Clinical Trials Unit<br>Boota, Nafisa; Leicester Clinical Trials Unit<br>Regan, Scott; University of Warwick Warwick Clinical Trials Unit<br>Bion, Julian; University of Birmingham, Intensive Care Medicine<br>Gordon, Anthony; Imperial College London, Division of Anaesthetics, Pain Medicine & Intensive Care<br>Lord, Janet; University of Birmingham, NIHR Surgical Reconstruction & Microbiology Research Centre<br>McAuley, Daniel; Queen's University Belfast, Centre for Experimental Medicine<br>Perkins, Gavin; University of Warwick Warwick Clinical Trials Unit; University Hospitals Birmingham NHS Foundation Trust<br>Singer, Mervyn; University College London, Bloomsbury Inst of Intensive Care Medicine<br>Young, Duncan; University of Oxford, Kadoorie Centre for Critical Care Research, Nuffield Division of Anaesthesia<br>Whitehouse, Tony; University Hospitals Birmingham NHS Foundation Trust, Department of Critical Care and Anaesthesia; University of Birmingham, Institute of Inflammation and Aging
**Primary Subject Heading**:	Intensive care
Secondary Subject Heading:	Intensive care
Keywords:	Adult intensive & critical care < INTENSIVE & CRITICAL CARE, MICROBIOLOGY, STATISTICS & RESEARCH METHODS, Clinical trials < THERAPEUTICS

SCHOLARONE™ Manuscripts

I, the Submitting Author has the right to grant and does grant on behalf of all authors of the Work (as defined in the below author licence), an exclusive licence and/or a non-exclusive licence for contributions from authors who are: i) UK Crown employees; ii) where BMJ has agreed a CC-BY licence shall apply, and/or iii) in accordance with the terms applicable for US Federal Government officers or employees acting as part of their official duties; on a worldwide, perpetual, irrevocable, royalty-free basis to BMJ Publishing Group Ltd ("BMJ") its licensees and where the relevant Journal is co-owned by BMJ to the co-owners of the Journal, to publish the Work in this journal and any other BMJ products and to exploit all rights, as set out in our licence.

The Submitting Author accepts and understands that any supply made under these terms is made by BMJ to the Submitting Author unless you are acting as an employee on behalf of your employer or a postgraduate student of an affiliated institution which is paying any applicable article publishing charge ("APC") for Open Access articles. Where the Submitting Author wishes to make the Work available on an Open Access basis (and intends to pay the relevant APC), the terms of reuse of such Open Access shall be governed by a Creative Commons licence – details of these licences and which Creative Commons licence will apply to this Work are set out in our licence referred to above.

Other than as permitted in any relevant BMJ Author's Self Archiving Policies, I confirm this Work has not been accepted for publication elsewhere, is not being considered for publication elsewhere and does not duplicate material already published. I confirm all authors consent to publication of this Work and authorise the granting of this licence.

For peer review only

Study into the Reversal of Septic Shock with Landiolol (Beta Blockade): STRESS-L. Study Protocol for a Randomised Trial

R Lall[1], E Skilton[1], D Mistry[1], N Boota[2], GD Perkins[1,3], AC Gordon[4], J Bion[5], D Young[6] DF McAuley[7,8], M Singer[9], J Lord[10], S Gates[11], S Regan[1], T Whitehouse[5]

1. Warwick Clinical Trials Unit, University of Warwick, Coventry, UK
2. Leicester Clinical Trials Unit, University of Leicester, Leicester, UK
3. Critical Care Directorate, University Hospitals Birmingham NHS Foundation Trust, Birmingham, UK
4. Faculty of Medicine, Department of Surgery and Cancer, Imperial College, London, UK
5. University Hospitals of Birmingham NHS Foundation Trust, Queen Elizabeth Hospital, Birmingham, UK
6. Kadoorie Centre for Critical Care Research, Nuffield Division of Anaesthesia, University of Oxford, Oxford, UK
7. Regional Intensive Care Unit, Royal Victoria Hospital, Belfast Health and Social Care Trust, Belfast, UK
8. The Wellcome Wolfson Institute for Experimental Medicine, Queens University Belfast, Belfast, UK
9. Centre for Intensive Care Medicine, Department of Medicine and Wolfson Institute for Biomedical Research, University College, London, UK
10. Institute of Inflammation and Ageing, Birmingham University Research labs., Queen Elizabeth Hospital, Birmingham, UK
11. Cancer Clinical Trials Unit, University of Birmingham, Birmingham, UK

Correspondence to Dr Tony Whitehouse Tony.Whitehouse@uhb.nhs.uk

Word Count: 4606 words

Keywords:

Septic Shock, tachycardia, Landiolol, beta blockade, SOFA score

For peer review only - http://bmjopen.bmj.com/site/about/guidelines.xhtml

Abstract

Introduction

In 2013, a single centre study reported the safe use of esmolol in patients with septic shock and tachycardia who required vasopressor therapy for more than 24 hours. Although not powered to detect a change in mortality, marked improvements were seen in survival (adjusted hazard ratio, 0.39; 95% CI, 0.26-0.59; p <0.001). Beta blockers are one of the most studied groups of drugs but their effect in septic shock is poorly understood; proposed mechanisms include not only the modulation of cardiac function but also immunomodulation.

Methods and analysis

STRESS-L is a randomised, open-label, non-blinded clinical trial which is enrolling a total of 340 patients with septic shock as defined by Sepsis-3 Consensus Definition and a tachycardia (HR ≥ 95 bpm) after vasopressor treatment of at least 24 hours. Standard randomisation (1:1 ratio) allocates patients to receive usual care (according to international standards) versus usual care and a continuous landiolol infusion to reduce the heart rate between 80 and 94 bpm. The primary endpoint is the mean SOFA score over 14 days from entry into the trial and whilst in ICU. Results will inform current clinical practice guidelines.

Ethics and dissemination

This trial has Clinical Trial Authorisation from the UK Competent Authority the Medicines and Healthcare products Regulatory Agency (MHRA) and has been approved by the East of England - Essex Research Ethics Committee reference: 17/EE/0368.

The results of the trial will be reported first to trial collaborators. The main report will be drafted by the trial co-ordinating team, and the final version will be agreed by the Trial Steering Committee before submission for publication, on behalf of the collaboration.

For peer review only

For peer review only - http://bmjopen.bmj.com/site/about/guidelines.xhtml

Registration

The trial is funded by the National Institute for Health Research (NIHR) Efficacy and Mechanism Evaluation (EME) (Project Number: EME - 14/150/85) and registered ISRCTN 12600919 and EudraCT: 2017-001785-14.

For peer review only

For peer review only - http://bmjopen.bmj.com/site/about/guidelines.xhtml

Strengths and limitations of this study

STRESS-L is a randomised study comparing the use of the ultra-short acting beta blocking agent, landiolol, with standard care in patients with septic shock, persistent tachycardia treated with high dose noradrenaline. It will study 340 patients, which we believe will be the largest study of its type

There is little know about the effects of beta blockade and the mechanisms induced by beta 1 receptor antagonism in this population. STRESS-L is collecting blood samples at intervals following randomisation to begin to understand mechanisms underlying beta blocker use.

Its limitation is that the study is not double-blind. Since the dosing of the Interventional Medicinal Product (IMP, landiolol) is variable according to a physiological endpoint (Target Heart Rate of 80 – 94 beats per minute), a placebo would be ineffective and the intervention impossible to blind.

For peer review only - http://bmjopen.bmj.com/site/about/guidelines.xhtml
For peer review only

Introduction

A randomised single centre study of 154 patients from Rome [1] reported the use of beta-adrenergic blockade using the short-acting agent esmolol in patients with septic shock and tachycardia who required vasopressor therapy for more than 24 hours. The dosing endpoint was a reduction in heart rate between 80 and 94 beats/min (bpm). Though this study was not powered to detect a change in outcome, marked improvements were seen in survival (adjusted hazard ratio, 0.39; 95% CI, 0.26-0.59; p <0.001). Improvements were also seen in the beta-blocker-treated group in the duration of vasopressor treatment, and in measures of renal and cardiac function.

Autonomic dysfunction and tachycardia are associated with a poor outcome in septic shock [2] with mortalities in excess of 70% reported [3]. Bradycardia is relatively protective [4]. Beta agonism is recommended as part of resuscitation and restoration of cardiac output and blood pressure in septic shock [5] despite an early study examining the use of dobutamine to increase cardiac index and systemic oxygen delivery was stopped prematurely because of an increase in the intervention cohort [6]. Beta blockade represents a paradigm shift in management of patients with septic shock and arises from observations of harm induced by excessive sympathetic activation and benefit from beta-adrenergic blockade. Limited data from animal models [7-12] and patients [1 13 14] suggest improved cardiovascular and immune function by their use. Excess beta-adrenergic activation may be both endogenous (related to the severity and duration of the underlying infection) and exogenous due to the catecholamine therapy which has until now been the mainstay of management of hypotension caused by septic shock [5]. A combination of beta2 agonism with beta1 blockade improves sepsis-induced immune, cardiovascular and coagulation dysfunction in established sepsis (Reviewed in [15]).

Landiolol (Rapibloc®, AOP Orphan Pharmaceuticals, Vienna, Austria) is an ultra-short acting beta blocker that has a half-life of 2.3 to 4 minutes [16] and is approximately 8 times more selective for the beta 1 receptor than esmolol [17]. Landiolol is metabolised by plasma pseudocholinesterase and liver carboxylesterse to inactive metabolites and its metabolism is unaffected by liver impairment [18]. A few preclinical studies have examined landiolol in sepsis. It decreased circulating levels of the cytokines, TNF-alpha, IL-6, and high mobility group box (HMGB)-1 and reduced histological lung damage in a rat endotoxin model [11]. landiolol was also cardio-protective in septic rats by normalising the expression of cardiac vasoactive peptide endothelin-1 [19]. Landiolol has been used safely and successfully in a septic patient with atrial fibrillation [20] and in sepsis-induced tachycardia [21]. A recent trial of 151

For peer review only - http://bmjopen.bmj.com/site/about/guidelines.xhtml

patients from 54 centres in Japan [22] demonstrated that landiolol could be safely administered to patients with septic shock.

Landiolol rather than esmolol was selected for this clinical trial because of its superior beta-1 specificity and the expectation that switching the landiolol infusion off would allow a rapid return to the baseline haemodynamic state if investigators deemed the intervention harmful. We will also report data of safety of landiolol infusion as part of the study.

For peer review only

Study Objectives

Primary Objective

The primary objective of this trial is to assess the efficacy, safety and mechanisms of landiolol (beta-blockade) in patients with septic shock and tachycardia requiring prolonged (>24 hours continuous treatment) support with high-dose vasopressor agents.

Secondary Objectives

The secondary objectives of the trial are:

- to determine whether infusion of the rapid-acting, ultra-short-lived and highly specific beta-adrenergic antagonist landiolol improves mortality and length of hospital stay compared with current best clinical practice, in patients who have septic shock.
- to investigate the pathways that are altered by beta blockade in septic shock by examining the effects of landiolol on blood markers of inflammation, metabolism and cardiomyocyte damage.

Methods and analysis

This manuscript has been written in concordance with the SPIRIT guidelines [23]

For peer review only - http://bmjopen.bmj.com/site/about/guidelines.xhtml

Trial design

This is a multi-centre, randomised, controlled open label phase IIb trial comparing usual treatment with usual treatment plus landiolol infusion in a total of 340 patients. The trial will be conducted and managed by Warwick Clinical Trials Unit and sponsored by the University of Hospitals of Birmingham NHS Foundation Trust. It is coordinated by a Trial Management Group and independent oversight is provided by the Trial Steering Committee and a Data Monitoring Committee.

The trial has been designed and will be reported in line with the CONSORT (Consolidated Standards of Reporting Trials) statement [24] and the conduct has been planned in full conformance with the principles of the Declaration of Helsinki and Good Clinical Practice.

Study setting

The study recruitment commenced in May 2018. The main trial is taking place in over 35 UK adult intensive care units (ICUs). Participating ICUs will have a typical case mix for UK critical care, a track record of recruitment to clinical trials, suitable support for screening and data collection and the means to store blood samples for transfer to the central analysis unit. They should also be willing to manage atrial fibrillation with correction of potassium, magnesium and amiodarone in the usual care group as the use of beta-blockers in these patients would risk making the trial results uninterpretable.

Inclusion criteria

Patients are eligible to be included in the trial if they meet the following criteria:

- Aged 18 years or above
- Being treated in a Critical Care Unit
- Septic shock according to internationally accepted definitions*
- Heart rate ≥95 bpm (at the time of randomisation)
- Receiving vasopressor support to maintain a target blood pressure for ≥24 hours
- Are being treated with noradrenaline at a rate ≥ 0.1mcg/kg/min

**Sepsis -3 definitions [25]:*

- ○ confirmed or suspected infection requiring antibiotic therapy
- ○ new organ dysfunction, as evidenced by an increase in SOFA score ≥2
- ○ a blood lactate >2 mmol/l at any point during shock resuscitation

For peer review only - http://bmjopen.bmj.com/site/about/guidelines.xhtml

- vasopressor therapy to maintain mean arterial pressure (MAP) ≥65 mmHg

In particular, the presence of a blood lactate > 2 mmol/l is only necessary for the diagnosis of septic shock and is NOT necessary for randomisation 24 hours later.

Exclusion criteria

The participant may not enter the trial if any of the following apply:

- Tachycardia as a result of pain, discomfort from medical devices (including endotracheal tubes), during interventions or other patient distress
- Any form of vasodilatory shock that is not caused by sepsis
- Noradrenaline infusion <0.1mcg/kg/min
- >72 hours after start of vasopressor therapy
- <12 hours since noradrenaline to treat a medical condition other than septic shock stopped
- Having pre-existing severe cardiac dysfunction (NYHA grade 4 or more)
- Having pre-existing severe pulmonary hypertension (mean PA pressures > 55mmHg)
- Acute severe bronchospasm (due to asthma or COPD)
- Untreated second or third degree heart block
- Untreated phaeochromocytoma
- Prinzmetal's angina
- A past history of ischaemic stroke or transient ischaemic attack (TIA) or untreated severe carotid stenosis.
- Advanced liver disease with Child-Pugh Score of ≥B.
- Known sensitivity to beta-blockers
- Patient / legal representative unwilling to provide written informed consent
- Known to be pregnant
- Terminal illness other than septic shock with a life expectancy < 28 days
- Participants who have been administered an investigational medicinal product for another research trial in the past 30 days
- Patients in whom the clinical team feel are about to finish their noradrenaline therapy
- Decision of withdrawal of care is in place or imminently anticipated
- Receiving extracorporeal membrane oxygenation (ECMO) treatment.

Co-enrolment of study participants onto other interventional studies will be considered where there is no possible conflict with the STRESS-L trial objectives.

For peer review only - http://bmjopen.bmj.com/site/about/guidelines.xhtml
For peer review only

Consent

Eligible patients who are deemed competent will be provided with a copy of the patient information sheet before informed consent is sought. However, due to the nature of the underlying condition and its treatment, many patients will be unable to give informed consent. The vulnerability of this patient group is fully appreciated and every effort must be undertaken to protect their safety and well-being. To ensure this, consenting will be obtained in accordance with the Medicines for Human Use Regulations and the Health Research Authority ethics guide on medical research involving adults who cannot consent (http://www.hra.nhs.uk/resources/before-you-apply/consent-and-participation/adults-unable-to-consent-for-themselves/).

For peer review only

Intervention

Participants will be randomised to either usual care (control group) or usual care and landiolol (intervention group).

Usual Care

Participants randomised to this arm will receive usual care for septic shock, namely

- Timely treatment of the source of sepsis (e.g. drainage of infected fluid collections);
- Prompt and appropriate empiric antibiotic treatment, and modification, if needed, on the basis of culture results;
- Appropriate fluid resuscitation to correct hypovolaemia;
- The use of vasopressors to achieve a target mean arterial pressure (suggested target 65-70 mmHg)

Adequate fluid resuscitation will be achieved using repeated fluid challenges to a guided target; the use of a cardiac output monitor is not mandated. The usual care group will not receive any beta blockade for the duration of their ICU stay - if the treating clinician deems beta blockade necessary, this will be captured on the Case Report Form and reported as a protocol deviation. All other general ICU management will be based on the latest guidance from the surviving sepsis campaign [5] and UK national critical care guidelines (e.g. ventilator and central line care bundles).

Usual care and landiolol

For peer review only - http://bmjopen.bmj.com/site/about/guidelines.xhtml

An intravenous infusion of landiolol will be started at 1.0 mcg/kg/min and will progressively increase every 15 minutes at increments of 1.0 mcg/kg/min, to reach the target heart rate of 80-94 bpm usually within 6 hours. Landiolol will be administered peripherally or centrally but must be on a dedicated line. Landiolol has an elimination half-life of 2.3 to 4 minutes [17] and so a loading dose is unnecessary. The landiolol infusion will be continued until the pulse rate is persistently below 95 bpm.

While a patient is receiving vasopressor agents (noradrenaline, vasopressin), the landiolol infusion will be adjusted accordingly to maintain the target heart rate of 80-94 bpm as per the landiolol infusion protocol (Figure 1: STRESS-L Study Drug Infusion Protocol). Once the patient is consistently within the target heart rate of 80-94 bpm, the landiolol Infusion will be continued and not be adjusted. The landiolol should be weaned and, if necessary, stopped whilst the HR is below 80 bpm. Once all vasopressor agents have been discontinued:

- if vasopressors have been discontinued for less than 12 hours, the landiolol infusion will continue (Figure 2: Timing and Weaning of the Study Drug).
- if vasopressors have been discontinued for more than 12 hours, the landiolol should be actively weaned (End of Noradrenaline Treatment EONT timepoint).

This trial allows for up to 14 days of landiolol treatment per participant.

Outcome measures

Primary outcome

The primary outcome will be the mean SOFA score over the first 14 days from entry into the trial and whilst in ICU. Measurement of the SOFA score will cease if the patient withdraws from the trial, dies or is discharged from the ICU. It is assumed that delayed discharges will be evenly distributed between each group.

Secondary outcomes

- Mortality at day 28 and day 90
- Length of ICU and hospital stay

For peer review only - http://bmjopen.bmj.com/site/about/guidelines.xhtml

- Reduction in dose and duration of vasopressor treatment (total daily administered doses)

Mechanistic outcomes

Serial blood samples will be collected from patients and assays will include markers of myocardial dysfunction and inflammation.

- Measurement of Total Catecholamine. It is unknown whether beta-blockade acts through altering the effects of extraneous catecholamines administered as treatment of septic shock or by modulating those produced by the patient. Serum catecholamines will be analysed in the context of the dose of inotropes being administered.
- Markers of myocardial dysfunction. Serum BNP has been demonstrated to be a reliable biomarker of myocardial injury, ischaemia and dysfunction in septic patients and also as a prognostic marker for a poor outcome [26 27]. Serial measurements of Troponin-T will be made.
- Measurement of serum Free Fatty acids and markers of Fatty Acid Metabolism.
- Biomarkers of systemic inflammation. This will be measured using a multiplex inflammatory biomarker assay depending on the available technology at the time of analysis. A selection of cytokines will be analysed, all or some of which will include IL-1beta, IL-2, IL-4, IL-5, IL-6, IL-8, IL-10, IL-12, TNF-alpha, TNF-beta, and IFN-gamma. This focused array will allow an assessment of the pro / anti-inflammatory balance over time in patients with septic shock and allow more detailed study of the other potential mechanisms of action of Landiolol. Cortisol assays will measure the influence of beta blockade on the adrenal cortex
- In addition, samples will be stored for subsequent analysis (e.g. genetics / proteomics / metabolomics) in order to investigate early cellular responses during the resolution of sepsis.

Safety outcomes

- The episodes of Bradycardia (HR <50 bpm), Bradycardia with haemodynamic compromise requiring intervention, significant hypotension requiring intervention (not including temporarily stopping the infusion), heart block, arrhythmia, arrhythmia with haemodynamic compromise requiring intervention will be reported.

For peer review only - http://bmjopen.bmj.com/site/about/guidelines.xhtml

Sample size

The primary outcome is the mean SOFA score over the first 14 days in ICU. Using preliminary collected data from 324 patients from UHB satisfying the trial eligibility criteria, the mean SOFA score over the first 14 days in ICU was 6.3, with standard deviation 2.4. Assuming (conservatively) a standard deviation of 2.8, and a difference of 1 point between the beta blocker and usual care groups, obtaining a p-value less than 0.05 (two-sided) with 90% power would require outcome data on 330 patients. To allow for 3% withdrawals and losses, the proposed sample size is 340 (Figure 3: STRESS-L CONSORT Flow diagram).

For peer review only

Assignment of Interventions

Randomisation

We will use a computerised minimisation randomisation system, created by Warwick Clinical Trials Unit in accordance with their standard operating procedure and held on a secure server, with the allocation generated per individual (participant). Participants will be randomised strictly sequentially as they become eligible for randomisation using a 1:1 ratio. The randomisation will be stratified by recruiting site and noradrenaline dose where the dose reflects the participant's severity. The dose will be dichotomised using a value of 0.3 mcg/kg/min (i.e. ≥0.1 mcg/kg/min to 0.3 mcg/kg/min and >0.3 mcg/kg/min) taken from the LeoPARDS trial as an indicator of low and high severity to ensure participant severity is balanced across both arms [28].

Once written informed consent has been obtained, a member of the local research team will use an interactive voice response (IVR) application to randomise. Eligibility will be confirmed by an investigator prior to randomisation. Sites will only be given access to the application once they have been given the 'green light' to begin recruitment and all required approvals are in place.

Blinding

This an open label trial with no blinding of the treatment allocation. Blinding is not possible due to the requirement for the landiolol dose to be titrated to achieve a target HR.

For peer review only - http://bmjopen.bmj.com/site/about/guidelines.xhtml

Data collection, management and analysis

Data Collection

Screening

All septic shock patients started on noradrenaline in ICU will be screened for trial eligibility. Once eligibility criteria are met there is a 48-hour window for randomisation. Due to this short window, informed consent may be sought during the first 24 hours of noradrenaline therapy. This will allow more time if legal representative consent is required. Randomisation will not occur until vasopressor therapy has been running for ≥24 hours, the patient has been treated with noradrenaline at rate >0.1 mcg/kg/min and remains tachycardic. It will be made clear to the patient or their legal representative that if they no longer meet the eligibility criteria after the 48-hour mark (i.e. 72 hours after the start of vasopressor therapy), the participant will not be randomised.

Baseline visit 24 hours prior and up to randomisation

Table 1 summarises the schedule of events for the trial. The patient's medical history, physical examination, basic demographic data and steroid use will be recorded. The participant will be randomised, and if allocated to the landiolol arm, IMP will be dispensed and the infusion started. As there is no checking and preparation of drug in the usual care arm, the landiolol infusion should be started within 1 hour of randomisation. The elements of the SOFA score will be recorded along with routine clinical data (cardiovascular, respiratory and renal physiological variables as well as haematological, biochemical and microbiological sample test results). The rates of noradrenaline infusion will be recorded hourly. The site and types of pathogens isolated following admission should be recorded on the eCRF.

Day 1 (time of randomisation to post 24 hours) up to day 14

The elements of the SOFA score will be recorded in the eCRF daily whilst the patient remains in the ICU from randomisation. This may be performed retrospectively but participating centres should ensure that blood has been sent to the Local Hospital Laboratories for the domains that depend on Laboratory Tests (liver, renal and coagulation and C-reactive protein). The rates of noradrenaline infusion and landiolol infusion (if randomised to this group) will be recorded hourly until day 2 to allow

For peer review only - http://bmjopen.bmj.com/site/about/guidelines.xhtml

comparison of noradrenaline dosing and then 6-hourly thereafter. Heart rate data will be collected to allow assessment of landiolol infusion compliance and separation between groups.

Other clinical data collected will be haemodynamics, respiratory variables (blood gases with the worst P/F ratio, type of ventilator support and previous 24 hours' intravenous fluid intake, urine output, and need for renal replacement therapy (as given in Table 1).

Follow up visits (Days 28 and 90)

Participants will also be followed up to ascertain survival status at 28 days and at 90 days post-randomisation. The participant's Community Physician will be contacted in the first instance to ascertain if the patient is alive; the participant may then be contacted by telephone. SAEs will be reported up to day 90 following randomisation.

Blood samples

Research blood samples will be collected on Days 0, 1, 2, 4 and 6 and the EONT visit (if this does not fall on a blood sampling day). Day 0 blood samples must be taken prior to the start of landiolol infusion. Days 1, 2, 4, 6 and EONT blood samples can be taken when is convenient within the 24-hour time period. The plasma will be removed and stored as per detailed instructions provided in the trial Laboratory manual. These research blood samples are mandatory as the results are required to answer the secondary outcomes of the trial.

Statistical Analysis

The primary analysis will be conducted according to intention to treat, comparing all those allocated to beta blocker (Landiolol) with all of those allocated to usual care, regardless of treatment received. Regression models will be used to estimate the treatment effects (with 95% confidence intervals), and the models will be adjusted for clinically important covariates. Subgroup effects for baseline severity measured using the dose of noradrenaline (noradrenaline ≥0.1 mcg/kg/min - 0.3 mcg/kg/min vs. >0.3 mcg/kg/min) and use of beta blockers at baseline (Yes/No) will be assessed using formal statistical tests for interaction for the primary outcome and mortality. If there is substantial non-compliance, we will conduct CACE (complier average causal effect) analyses to estimate the treatment effect among those who received the treatment as allocated. The DMC will meet every 6 months to closely monitor the accumulating data, focusing on safety. A detailed

For peer review only - http://bmjopen.bmj.com/site/about/guidelines.xhtml

statistical analysis plan (SAP) will be written by the study statistical team and then finalised and approved by the DMC before any analysis is undertaken.

Missing data

Every effort will be made to minimise missing outcome data in this trial. Further exploratory analyses to assess the impact of any missing outcome data on the SOFA-score will be examined using multiple imputation techniques and other methods specified in the SAP.

Data Monitoring

The Warwick Clinical Trials Unit will be responsible for trial monitoring and visits will be conducted in accordance with the monitoring plan. On-site monitoring visits during the trial will be check the accuracy of data entered into the clinical trial database against the source documents, adherence to the protocol, procedures and Good Clinical Practice and the progress of patient recruitment and follow-up.

Safety, ethics and dissemination

Adverse outcomes

All serious adverse events and suspected unexpected serious adverse events occurring from the time of randomisation to the final follow up visit at day 90 will be recorded on the Case Record Form and faxed or emailed to the coordinating centre within 24 hours of the research staff becoming aware of the event. In particular, bradycardia (HR <50 bpm) with haemodynamic compromise requiring intervention, heart block, significant hypotension requiring intervention, arrhythmia with haemodynamic compromise requiring intervention should be recorded. Adverse event/reactions will be assessed for seriousness and reported in accordance with MHRA guidelines.

For peer review only

For peer review only - http://bmjopen.bmj.com/site/about/guidelines.xhtml

Regulatory and ethics approvals

The East of England - Essex Research Ethics Committee (REC: 19/EE/0368 - flagged for trials involving clinical trials in patients without capacity) and Medical Healthcare Regulatory Authority (MHRA) (EudraCT 2017-001785-14) have approved the study protocol. The ICRSTN trial registration number is ISRCTN12600919. The study will comply with the principles for sharing clinical trial data from publicly funded clinical trials.

Dissemination

The study will be reported in accordance with the Consolidated Standards of Reporting Trials (CONSORT) guidelines [24]. The study findings will be presented at national and international meetings with abstracts on-line. Presentation at these meetings will ensure that results and any implications quickly reach all of the UK and international intensive care community. In accordance with the open access policies proposed by the NIHR, we aim to publish the clinical findings of the trial in high quality peer-reviewed open access (via Pubmed) journals. Finally, an on-going update of the trial will also be provided on the Warwick Clinical Trials Unit website and social media platforms e.g. Twitter (@STRESSL_trial).

Confidentiality

In order to maintain confidentiality, all CRFs, questionnaires, study reports and communication regarding the study will identify the patients by the assigned unique trial identifier and initials only. Patient confidentiality will be maintained at every stage and will not be made publicly available to the extent permitted by the applicable laws and regulations.

Patient and public involvement

This application is informed through a series of meetings with survivors and carers of patients who have had septic shock. Patient and public contributed to the trial proposal, protocol development and design of patient facing materials. During the conduct of the trial a Patient and Public Involvement (PPI) member has been part of the Trial Management Group and has reviewed patient

For peer review only - http://bmjopen.bmj.com/site/about/guidelines.xhtml

facing documentation prior to the ethics and regulatory submissions and their comments have been incorporated. Two PPI representatives will sit on the Trial Steering Committee and will provide input from a patient perspective at trial meetings. Both representatives will review and provide feedback on all relevant project documents.

Discussion

Many large multi-centre trials of sepsis and septic shock have not derived statistical differences between study groups following single centre pilot studies suggesting large mortality improvements [29-31]. This is possibly because of the wide variation in severity of illness included in those study. STRESS-L includes patients who are predicted to be at risk of a high mortality because they remain tachycardic (HR >= 95 bpm) after 24 hours of noradrenaline treatment. The persistence of tachycardia may phenotypically define a genetically different cohort as suggested in the Acute Respiratory Distress Syndrome (ARDS) population [32]. The trial uses landiolol because of its exceptional short half-life and highly specific beta1 receptor blockade.

The primary outcome measure (mean SOFA score), which correlates with survival, allows measurement of the effect of landiolol on organ function compared with usual care. Analysis of each domain in the SOFA score will also begin to allow us to distinguish whether alteration in cardiac efficiency is the sole mechanism for differences between groups. The collection and analysis of blood samples will also characterise further the role of the adrenergic system in septic shock and proposed genetic predisposition to this specific group of patients with septic shock.

STRESS-L recruited its first patient in April 2018 but a series of protocol amendments (owing to a complex intervention) and the COVID-19 pandemic has delayed the submission of this protocol patient. As of submission, there have been 109 patients recruited, approximately 32% of its target.

For peer review only - http://bmjopen.bmj.com/site/about/guidelines.xhtml

Contributions

TW and JFB conceived the initial trial concept and helped develop the trial design and protocol. RL, DM and SG carried out the power calculations and with ACG, JL, GDP, DFM, MS, DY helped develop the trial design and protocol. NB, ES, SR oversaw the governance of the protocol. TW is the chief investigator and takes overall responsibility for all aspects of trial design, the protocol and trial conduct. All authors read and approved the final manuscript.

Competing Interests

TW reports grants from NIHR Efficacy and Mechanism Evaluation for the funding of STRESS-L (Project Number: EME - 14/150/85) and during the conduct of the study; personal fees and non-financial support from AOP Orphan, manufacturer of Landiolol, outside the submitted work; MS has received travel expenses from AOP Orphan for delivering lectures.

MS reports grants and other from NewB, grants from DSTL, other from Amormed, other from Biotest, other from GE, other from Baxter, grants from Critical Pressure, grants from Apollo Therapeutics, other from Roche, other from Bayer, other from Shionogi, outside the submitted work; .

ACG reports receiving grants from the NIHR and the NIHR Research Professorship; nonfinancial support from the NIHR Clinical Research Network and the NIHR Imperial Biomedical Research Centre during the conduct of the study; and personal fees from GlaxoSmithKline and Bristol-Myers Squibb outside the submitted work.

DFMc reports a grant from the NIHR EME programme for this study. Outside the submitted work, DFM reports personal fees from consultancy for GlaxoSmithKline, Boehringer Ingelheim, Bayer, Novartis and Eli Lilly. In addition, his institution has received funds from grants from the NIHR, Wellcome Trust, Innovate-UK and others. In addition, DFM has a patent issued to his institution for a treatment for ARDS. DFM is a Director of Research for the Intensive Care Society and NIHR EME Programme Director.

GP reports grants from National Institute for Health Research, during the conduct of the study; and Director of Research, Intensive Care Society.

All other authors declare they have no competing interests.

For peer review only - http://bmjopen.bmj.com/site/about/guidelines.xhtml
For peer review only

Acknowledgements

The authors would like to thank our Patient and Public Involvement (PPI) group and AOP Orphan, Vienna, Austria, for providing the landiolol for the trial free of charge. ACG is the Deputy Director and GDP & DFM are Co-directors of Research for the Intensive Care Foundation. We are grateful for the infrastructure provided by the National Institute of Health Research (NIHR) Critical Care Specialty Group of the Comprehensive Clinical Research Network. We would like to express our gratitude to the members of the Trial Steering Committee and the Data Monitoring Committee who provide support and guidance for this study.

Funding

This project was funded by the Efficacy and Mechanism Evaluation (EME) Programme and managed by the NIHR (Project Number - 14/150/85). ACG is funded by a NIHR Clinician Scientist Fellowship. MS and GDP are supported by NIHR Senior Investigators. The views expressed in this publication are those of the author(s) and not necessarily those of the EME, NHS, NIHR or the Department of Health.

For peer review only

For peer review only - http://bmjopen.bmj.com/site/about/guidelines.xhtml

References

1. Morelli A, Ertmer C, Westphal M, et al. Effect of heart rate control with esmolol on hemodynamic and clinical outcomes in patients with septic shock: a randomized clinical trial. *JAMA* 2013;310(16):1683-91. doi: 1752246 [pii];10.1001/jama.2013.278477 [doi]
2. Schwartz A, Brotfain E, Koyfman L, et al. Cardiac Arrhythmias in a Septic ICU Population: A Review. *J Crit Care Med (Targu Mures)* 2015;1(4):140-46. doi: 10.1515/jccm-2015-0027 [published Online First: 2015/11/10]
3. Schmidt H, Muller-Werdan U, Hoffmann T, et al. Autonomic dysfunction predicts mortality in patients with multiple organ dysfunction syndrome of different age groups. *Crit Care Med* 2005;33(9):1994-2002. doi: 10.1097/01.ccm.0000178181.91250.99 [published Online First: 2005/09/09]
4. Beesley SJ, Wilson EL, Lanspa MJ, et al. Relative Bradycardia in Patients With Septic Shock Requiring Vasopressor Therapy. *Crit Care Med* 2017;45(2):225-33. doi: 10.1097/CCM.0000000000002065 [published Online First: 2016/09/13]
5. Dellinger RP, Levy MM, Rhodes A, et al. Surviving Sepsis Campaign: international guidelines for management of severe sepsis and septic shock, 2012. *Intensive Care Med* 2013;39(2):165-228. doi: 10.1007/s00134-012-2769-8 [doi]
6. Hayes MA, Timmins AC, Yau EH, et al. Elevation of systemic oxygen delivery in the treatment of critically ill patients. *N Engl J Med* 1994;330(24):1717-22. doi: 10.1056/NEJM199406163302404 [doi]
7. Berk JL, Hagen JF, Beyer WH, et al. The treatment of endotoxin shock by beta adrenergic blockade. *Ann Surg* 1969;169(1):74-81.
8. Suzuki T, Morisaki H, Serita R, et al. Infusion of the beta-adrenergic blocker esmolol attenuates myocardial dysfunction in septic rats. *Crit Care Med* 2005;33(10):2294-301. doi: 00003246-200510000-00021 [pii]
9. Aboab J, Sebille V, Jourdain M, et al. Effects of esmolol on systemic and pulmonary hemodynamics and on oxygenation in pigs with hypodynamic endotoxin shock. *Intensive Care Med* 2011;37(8):1344-51. doi: 10.1007/s00134-011-2236-y [doi]
10. Ackland GL, Yao ST, Rudiger A, et al. Cardioprotection, attenuated systemic inflammation, and survival benefit of beta1-adrenoceptor blockade in severe sepsis in rats. *Crit Care Med* 2010;38(2):388-94. doi: 10.1097/CCM.0b013e3181c03dfa [doi]
11. Hagiwara S, Iwasaka H, Maeda H, et al. Landiolol, an ultrashort-acting beta1-adrenoceptor antagonist, has protective effects in an LPS-induced systemic inflammation model. *Shock* 2009;31(5):515-20. doi: 10.1097/SHK.0b013e3181863689 [doi]
12. Stolk RF, van der Pasch E, Naumann F, et al. Norepinephrine Dysregulates the Immune Response and Compromises Host Defense during Sepsis. *Am J Respir Crit Care Med* 2020;202(6):830-42. doi: 10.1164/rccm.202002-0339OC [published Online First: 2020/06/11]
13. Balik M, Rulisek J, Leden P, et al. Concomitant use of beta-1 adrenoreceptor blocker and norepinephrine in patients with septic shock. *Wien Klin Wochenschr* 2012;124(15-16):552-56. doi: 10.1007/s00508-012-0209-y [doi]
14. Sanfilippo F, Santonocito C, Morelli A, et al. Beta-blocker use in severe sepsis and septic shock: a systematic review. *Curr Med Res Opin* 2015;31(10):1817-25. doi: 10.1185/03007995.2015.1062357 [doi]
15. de ME, Aboab J, Mansart A, et al. Bench-to-bedside review: Beta-adrenergic modulation in sepsis. *Crit Care* 2009;13(5):230. doi: cc8026 [pii];10.1186/cc8026 [doi]
16. Atarashi H, Kuruma A, Yashima M, et al. Pharmacokinetics of landiolol hydrochloride, a new ultra-short-acting beta-blocker, in patients with cardiac arrhythmias. *Clin Pharmacol Ther* 2000;68(2):143-50. doi: S0009923600082473 [pii];10.1067/mcp.2000.108733 [doi]

For peer review only - http://bmjopen.bmj.com/site/about/guidelines.xhtml

17. Iguchi S, Iwamura H, Nishizaki M, et al. Development of a highly cardioselective ultra short-acting beta-blocker, ONO-1101. *Chem Pharm Bull (Tokyo)* 1992;40(6):1462-69.
18. Takahata T, Yasui-Furukori N, Sakamoto J, et al. Influence of hepatic impairment on the pharmacokinetics and pharmacodynamics of landiolol hydrochloride, an ultra-short-acting beta1-blocker. *Drugs R D* 2005;6(6):385-94. doi: 666 [pii]
19. Seki Y, Jesmin S, Shimojo N, et al. Significant reversal of cardiac upregulated endothelin-1 system in a rat model of sepsis by landiolol hydrochloride. *Life Sci* 2014 doi: S0024-3205(14)00405-6 [pii];10.1016/j.lfs.2014.04.005 [doi]
20. Yoshida Y, Hongo T, Sakamoto A, et al. Successful management of tachycardiac atrial fibrillation in a septic patient with landiolol. *Anesth Analg* 2005;100(1):294. doi: 100/1/294-a [pii];10.1213/01.ANE.0000140814.28118.6F [doi]
21. Arita Y, Segawa T, Yamamoto S, et al. Landiolol is effective for the treatment of tachycardia-induced cardiogenic shock in patients during septic shock therapy. *BMJ Case Reports* 2017;2017:bcr-2017-222268. doi: 10.1136/bcr-2017-222268
22. Kakihana Y, Nishida O, Taniguchi T, et al. Efficacy and safety of landiolol, an ultra-short-acting β1-selective antagonist, for treatment of sepsis-related tachyarrhythmia (J-Land 3S): a multicentre, open-label, randomised controlled trial. *Lancet Respir Med* 2020;8(9):863-72. doi: 10.1016/s2213-2600(20)30037-0 [published Online First: 2020/04/04]
23. Chan AW, Tetzlaff JM, Gotzsche PC, et al. SPIRIT 2013 explanation and elaboration: guidance for protocols of clinical trials. *BMJ* 2013;346:e7586. doi: 10.1136/bmj.e7586 [published Online First: 2013/01/11]
24. Moher D, Hopewell S, Schulz KF, et al. CONSORT 2010 explanation and elaboration: updated guidelines for reporting parallel group randomised trials. *BMJ* 2010;340:c869. doi: 10.1136/bmj.c869 [published Online First: 2010/03/25]
25. Singer M, Deutschman CS, Seymour CW, et al. The Third International Consensus Definitions for Sepsis and Septic Shock (Sepsis-3). *JAMA* 2016;315(8):801-10. doi: 2492881 [pii];10.1001/jama.2016.0287 [doi]
26. Charpentier J, Luyt CE, Fulla Y, et al. Brain natriuretic peptide: A marker of myocardial dysfunction and prognosis during severe sepsis. *Crit Care Med* 2004;32(3):660-65. doi: 00003246-200403000-00007 [pii]
27. Post F, Weilemann LS, Messow CM, et al. B-type natriuretic peptide as a marker for sepsis-induced myocardial depression in intensive care patients. *Crit Care Med* 2008;36(11):3030-37. doi: 10.1097/CCM.0b013e31818b9153 [doi]
28. Gordon AC, Perkins GD, Singer M, et al. Levosimendan for the Prevention of Acute Organ Dysfunction in Sepsis. *N Engl J Med* 2016;375(17):1638-48. doi: 10.1056/NEJMoa1609409 [doi]
29. Australian N-SSIft, New Zealand Intensive Care Society Clinical Trials G, the Canadian Critical Care Trials G, et al. Intensive versus conventional glucose control in critically ill patients with traumatic brain injury: long-term follow-up of a subgroup of patients from the NICE-SUGAR study. *Intensive Care Med* 2015;41(6):1037-47. doi: 10.1007/s00134-015-3757-6 [published Online First: 2015/06/20]
30. Venkatesh B, Finfer S, Cohen J, et al. Adjunctive Glucocorticoid Therapy in Patients with Septic Shock. *New England Journal of Medicine* 2018;378(9):797-808. doi: 10.1056/NEJMoa1705835
31. Ranieri VM, Thompson BT, Barie PS, et al. Drotrecogin alfa (activated) in adults with septic shock. *N Engl J Med* 2012;366(22):2055-64. doi: 10.1056/NEJMoa1202290 [doi]
32. Calfee CS, Janz DR, Bernard GR, et al. Distinct Molecular Phenotypes of Direct Versus Indirect ARDS in Single and Multi-Center Studies. *Chest* 2014 doi: 2022041 [pii];10.1378/chest.14-2454 [doi]

For peer review only - http://bmjopen.bmj.com/site/about/guidelines.xhtml

For peer review only

For peer review only - http://bmjopen.bmj.com/site/about/guidelines.xhtml

Figure Legends

Figure 1: STRESS-L Study Drug Infusion Protocol

Figure 2: STRESS-L Protocol for Weaning and Stopping of Study Drug

Figure 3: STRESS-L CONSORT Flow diagram

Table 1: STRESS-L schedule of interventions

For peer review only

For peer review only - http://bmjopen.bmj.com/site/about/guidelines.xhtml

Procedure (Time (T) in days/hours)	Screening (T0-12)	Baseline- (Day 0 - 24hr – T0)	Day 1 (T0+24)	Day 2	Day 3	Day 4	Day 5	Day 6	Day 7	Day 8	Day 9	Day 10	Day 11	Day 12	Day 13	Day 14	EONT Visit	FU Visit Day 28	Final visit Day 90
Eligibility assessment	•																		
Informed consent		•																	
Randomisation		•																	
Demographics		•																	
Medical History		•																	
ECG		•	According to clinical need or if AE/SAE																
Pregnancy test		•																	
IMP		Dispense														End			
Blood sample		•	•	•		•		•									•		
Biobank blood sample (optional)		•	•														•		
Transport of stored serum																	Batch		
Local laboratory tests (normal		•	•	•	•	•	•	•	•	•	•	•	•	•	•	•	•		
C-Reactive Protein (CRP)		•	•	•	•	•	•	•	•	•	•	•	•	•	•	•	•		
Glucose		•	•	•		•		•									•		
Lactate		•	•	•		•		•									•		
Worst PaO2/FiO2		•	•	•	•	•	•	•	•	•	•	•	•	•	•	•	•		
Platelets		•	•	•	•	•	•	•	•	•	•	•	•	•	•	•	•		
Creatinine		•	•	•	•	•	•	•	•	•	•	•	•	•	•	•	•		
Bilirubin		•	•	•	•	•	•	•	•	•	•	•	•	•	•	•	•		
White Cell Count		•	•	•	•	•	•	•	•	•	•	•	•	•	•	•	•		
Liver Function Tests (ALT or AST)		•	•	•		•		•									•		
Central Venous Blood Gas / Arterial		•	•	•		•		•									•		
Microbiology results from local lab		•	•	•	•	•	•	•	•	•	•	•	•	•	•	•	•		
Heart Rate (Hourly: T0+7 days)		•	•	•	•	•	•	•	•	•	•	•	•	•	•	•	•		
Atrial Fibrillation (Hourly: T0+7 days)		•	•	•	•	•	•												
Blood Pressure (Hourly: T0+7 days)		•	•	•	•	•	•												
Rate of Vasopressor / inotropes		•	•	•	•	•	•	•	•	•	•	•	•	•	•	•	•		
In/Out Fluids		•	•	•	•	•	•	•	•	•	•	•	•	•	•	•	•		
SOFA score		•	•	•	•	•	•	•	•	•	•	•	•	•	•	•	•		
Adverse Events (SAEs up to Day 90)		•	•	•	•	•	•	•	•	•	•	•	•	•	•	•	•	•	•
Steroid use		•	•	•		•		•									•		
Compliance		•	•	•	•	•	•	•	•	•	•	•	•	•	•	•	•		
Mortality status																		•	•
End of Trial																			•

Table 1: STRESS-L schedule of interventions

297x209mm (300 x 300 DPI)

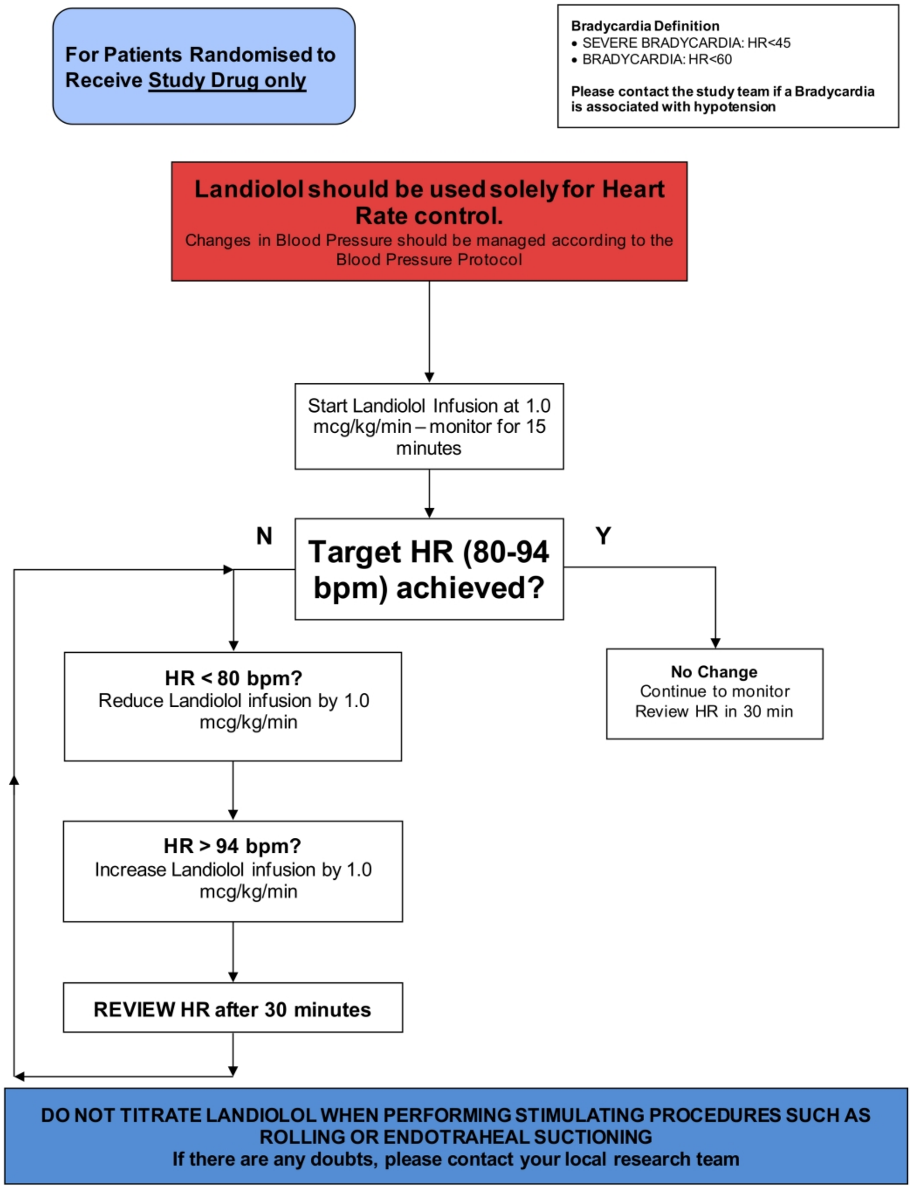

Figure 1: STRESS-L Study Drug Infusion Protocol

197x248mm (299 x 299 DPI)

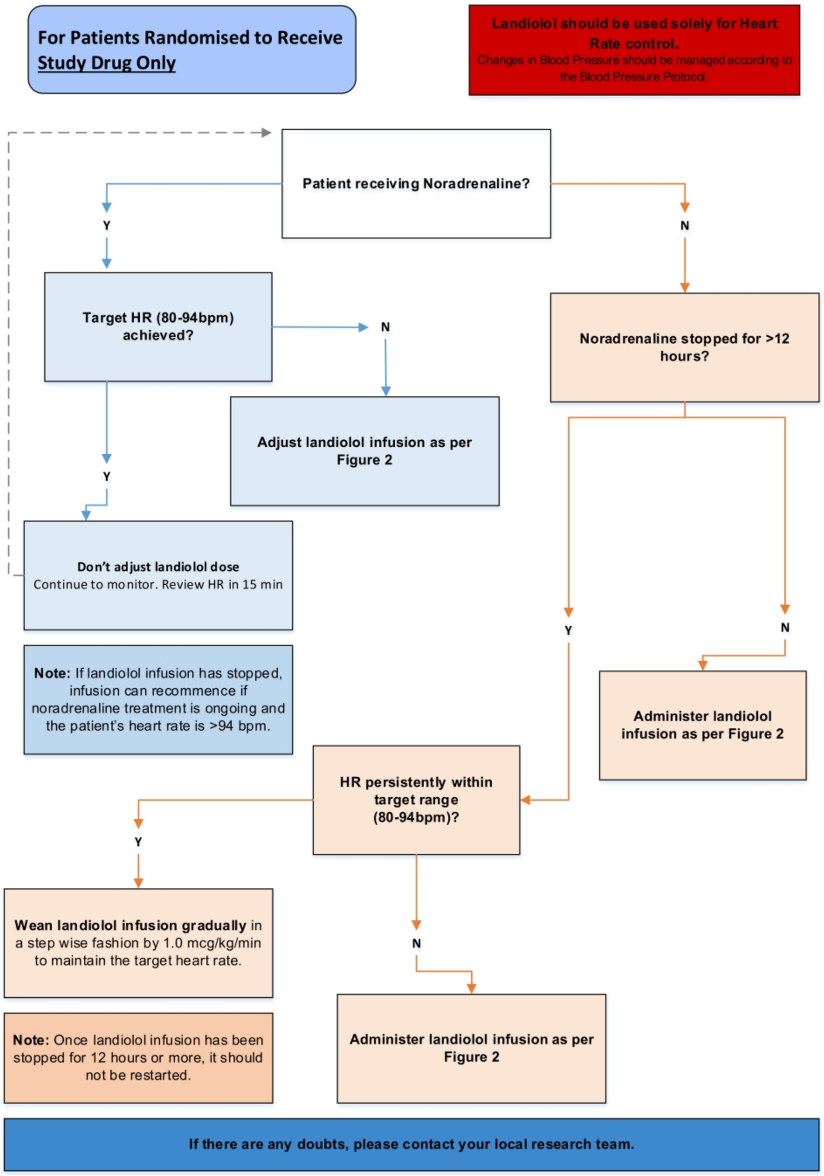

Figure 2: STRESS-L Protocol for Weaning and Stopping of Study Drug

198x267mm (299 x 299 DPI)

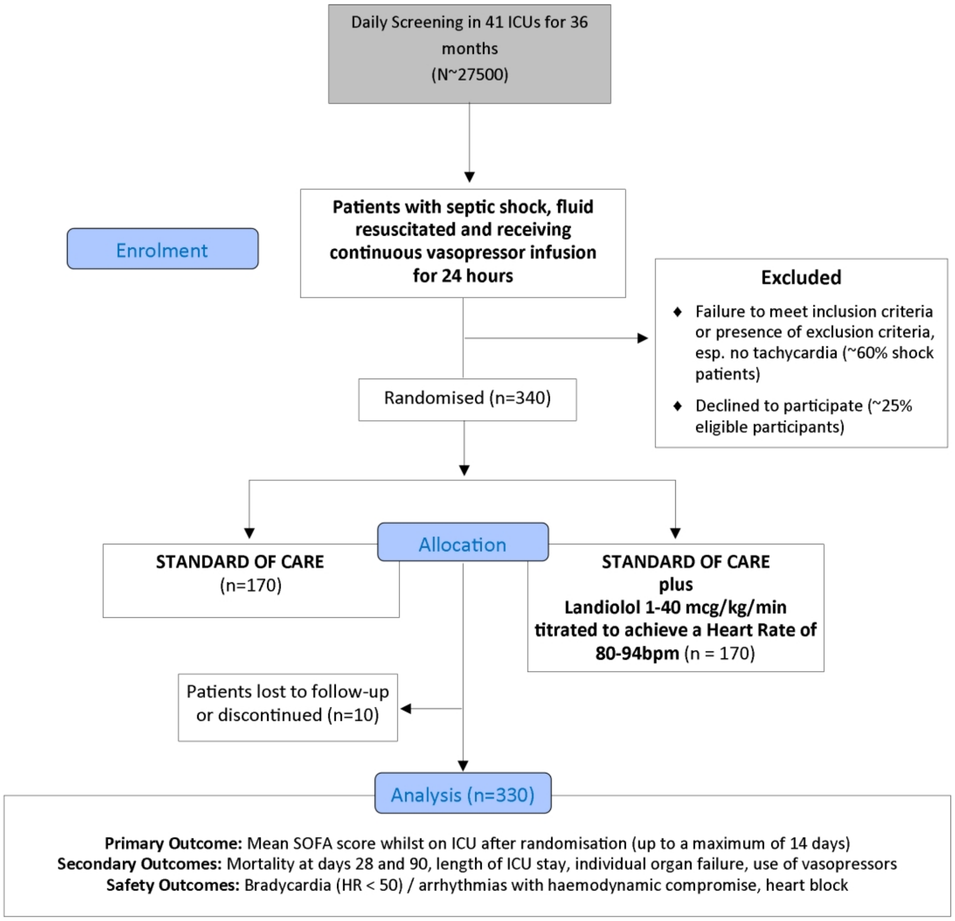

Figure 3: STRESS-L CONSORT Flow diagram

195x196mm (300 x 300 DPI)

BMJ Open

Study into the Reversal of Septic Shock with Landiolol (Beta Blockade): STRESS-L. Study Protocol for a Randomised Trial

Journal:	*BMJ Open*
Manuscript ID	bmjopen-2020-043194.R2
Article Type:	Protocol
Date Submitted by the Author:	20-Jan-2021
Complete List of Authors:	Lall, Ranjit; University of Warwick Warwick Clinical Trials Unit<br>Mistry, Dipesh; University of Warwick Warwick Clinical Trials Unit<br>Skilton, Emma; University of Warwick Warwick Clinical Trials Unit<br>Boota, Nafisa; Leicester Clinical Trials Unit<br>Regan, Scott; University of Warwick Warwick Clinical Trials Unit<br>Bion, Julian; University of Birmingham, Intensive Care Medicine<br>Gates, Simon; University of Birmingham, Cancer Research UK Clinical Trials Unit; University of Warwick, Clinical Trials Unit<br>Gordon, Anthony; Imperial College London, Division of Anaesthetics, Pain Medicine & Intensive Care<br>Lord, Janet; University of Birmingham, NIHR Surgical Reconstruction & Microbiology Research Centre<br>McAuley, Daniel; Queen's University Belfast, Centre for Experimental Medicine<br>Perkins, Gavin; University of Warwick Warwick Clinical Trials Unit; University Hospitals Birmingham NHS Foundation Trust<br>Singer, Mervyn; University College London, Bloomsbury Inst of Intensive Care Medicine<br>Young, Duncan; University of Oxford, Kadoorie Centre for Critical Care Research, Nuffield Division of Anaesthesia<br>Whitehouse, Tony; University Hospitals Birmingham NHS Foundation Trust, Department of Critical Care and Anaesthesia; University of Birmingham, Institute of Inflammation and Aging
**Primary Subject Heading**:	Intensive care
Secondary Subject Heading:	Intensive care
Keywords:	Adult intensive & critical care < INTENSIVE & CRITICAL CARE, MICROBIOLOGY, STATISTICS & RESEARCH METHODS, Clinical trials < THERAPEUTICS

SCHOLARONE™
Manuscripts

I, the Submitting Author has the right to grant and does grant on behalf of all authors of the Work (as defined in the below author licence), an exclusive licence and/or a non-exclusive licence for contributions from authors who are: i) UK Crown employees; ii) where BMJ has agreed a CC-BY licence shall apply, and/or iii) in accordance with the terms applicable for US Federal Government officers or employees acting as part of their official duties; on a worldwide, perpetual, irrevocable, royalty-free basis to BMJ Publishing Group Ltd ("BMJ") its licensees and where the relevant Journal is co-owned by BMJ to the co-owners of the Journal, to publish the Work in this journal and any other BMJ products and to exploit all rights, as set out in our licence.

The Submitting Author accepts and understands that any supply made under these terms is made by BMJ to the Submitting Author unless you are acting as an employee on behalf of your employer or a postgraduate student of an affiliated institution which is paying any applicable article publishing charge ("APC") for Open Access articles. Where the Submitting Author wishes to make the Work available on an Open Access basis (and intends to pay the relevant APC), the terms of reuse of such Open Access shall be governed by a Creative Commons licence – details of these licences and which Creative Commons licence will apply to this Work are set out in our licence referred to above.

Other than as permitted in any relevant BMJ Author's Self Archiving Policies, I confirm this Work has not been accepted for publication elsewhere, is not being considered for publication elsewhere and does not duplicate material already published. I confirm all authors consent to publication of this Work and authorise the granting of this licence.

For peer review only

Study into the Reversal of Septic Shock with Landiolol (Beta Blockade): STRESS-L. Study Protocol for a Randomised Trial

R Lall[1], D Mistry[1], E Skilton[1], N Boota[2], S Regan[1], J Bion[3], S Gates[4], AC Gordon[5], J Lord[6], DF McAuley[7,8], GD Perkins[1,9], M Singer[10], D Young[11], T Whitehouse[3]

1. Warwick Clinical Trials Unit, University of Warwick, Coventry, UK
2. Leicester Clinical Trials Unit, University of Leicester, Leicester, UK
3. University Hospitals of Birmingham NHS Foundation Trust, Queen Elizabeth Hospital, Birmingham, UK
4. Cancer Clinical Trials Unit, University of Birmingham, Birmingham, UK
5. Faculty of Medicine, Department of Surgery and Cancer, Imperial College, London, UK
6. Institute of Inflammation and Ageing, Birmingham University Research labs., Queen Elizabeth Hospital, Birmingham, UK
7. Regional Intensive Care Unit, Royal Victoria Hospital, Belfast Health and Social Care Trust, Belfast, UK
8. The Wellcome Wolfson Institute for Experimental Medicine, Queens University Belfast, Belfast, UK
9. Critical Care Directorate, University Hospitals Birmingham NHS Foundation Trust, Birmingham, UK
10. Centre for Intensive Care Medicine, Department of Medicine and Wolfson Institute for Biomedical Research, University College, London, UK
11. Kadoorie Centre for Critical Care Research, Nuffield Division of Anaesthesia, University of Oxford, Oxford, UK

Correspondence to Dr Tony Whitehouse Tony.Whitehouse@uhb.nhs.uk

Word Count: 4606 words

Keywords:

Septic Shock, tachycardia, Landiolol, beta blockade, SOFA score

For peer review only - http://bmjopen.bmj.com/site/about/guidelines.xhtml

Abstract

Introduction

In 2013, a single centre study reported the safe use of esmolol in patients with septic shock and tachycardia who required vasopressor therapy for more than 24 hours. Although not powered to detect a change in mortality, marked improvements were seen in survival (adjusted hazard ratio, 0.39; 95% CI, 0.26-0.59; p <0.001). Beta blockers are one of the most studied groups of drugs but their effect in septic shock is poorly understood; proposed mechanisms include not only the modulation of cardiac function but also immunomodulation.

Methods and analysis

STRESS-L is a randomised, open-label, non-blinded clinical trial which is enrolling a total of 340 patients with septic shock as defined by Sepsis-3 Consensus Definition and a tachycardia (HR ≥ 95 bpm) after vasopressor treatment of at least 24 hours. Standard randomisation (1:1 ratio) allocates patients to receive usual care (according to international standards) versus usual care and a continuous landiolol infusion to reduce the heart rate between 80 and 94 bpm. The primary endpoint is the mean SOFA score over 14 days from entry into the trial and whilst in ICU. Results will inform current clinical practice guidelines.

Ethics and dissemination

This trial has Clinical Trial Authorisation from the UK Competent Authority the Medicines and Healthcare products Regulatory Agency (MHRA) and has been approved by the East of England - Essex Research Ethics Committee reference: 17/EE/0368.

The results of the trial will be reported first to trial collaborators. The main report will be drafted by the trial co-ordinating team, and the final version will be agreed by the Trial Steering Committee before submission for publication, on behalf of the collaboration.

For peer review only

For peer review only - http://bmjopen.bmj.com/site/about/guidelines.xhtml

Registration

The trial is funded by the National Institute for Health Research (NIHR) Efficacy and Mechanism Evaluation (EME) (Project Number: EME - 14/150/85) and registered ISRCTN 12600919 and EudraCT: 2017-001785-14.

For peer review only

For peer review only - http://bmjopen.bmj.com/site/about/guidelines.xhtml

Strengths and limitations of this study

STRESS-L is a randomised study comparing the use of the ultra-short acting beta blocking agent, landiolol, with standard care in patients with septic shock, persistent tachycardia treated with high dose noradrenaline.

STRESS-L will study 340 patients, which we believe will be the largest study of its type.

There is little known about the effects of beta blockade and the mechanisms induced by beta 1 receptor antagonism in this population.

Its limitation is that the study is not double-blind since the dosing of the Interventional Medicinal Product (IMP, landiolol) is variable according to a physiological endpoint (Target Heart Rate of 80 – 94 beats per minute), a placebo would be ineffective and the intervention impossible to blind.

For peer review only

For peer review only - http://bmjopen.bmj.com/site/about/guidelines.xhtml

Introduction

A randomised single centre study of 154 patients from Rome [1] reported the use of beta-adrenergic blockade using the short-acting agent esmolol in patients with septic shock and tachycardia who required vasopressor therapy for more than 24 hours. The dosing endpoint was a reduction in heart rate between 80 and 94 beats/min (bpm). Though this study was not powered to detect a change in outcome, marked improvements were seen in survival (adjusted hazard ratio, 0.39; 95% CI, 0.26-0.59; p <0.001). Improvements were also seen in the beta-blocker-treated group in the duration of vasopressor treatment, and in measures of renal and cardiac function.

Autonomic dysfunction and tachycardia are associated with a poor outcome in septic shock [2] with mortalities in excess of 70% reported [3]. Bradycardia is relatively protective [4]. Beta agonism is recommended as part of resuscitation and restoration of cardiac output and blood pressure in septic shock [5] despite an early study examining the use of dobutamine to increase cardiac index and systemic oxygen delivery was stopped prematurely because of an increase in the intervention cohort [6]. Beta blockade represents a paradigm shift in management of patients with septic shock and arises from observations of harm induced by excessive sympathetic activation and benefit from beta-adrenergic blockade. Limited data from animal models [7-12] and patients [1 13 14] suggest improved cardiovascular and immune function by their use. Excess beta-adrenergic activation may be both endogenous (related to the severity and duration of the underlying infection) and exogenous due to the catecholamine therapy which has until now been the mainstay of management of hypotension caused by septic shock [5]. A combination of beta2 agonism with beta1 blockade improves sepsis-induced immune, cardiovascular and coagulation dysfunction in established sepsis (Reviewed in [15]).

Landiolol (Rapibloc®, AOP Orphan Pharmaceuticals, Vienna, Austria) is an ultra-short acting beta blocker that has a half-life of 2.3 to 4 minutes [16] and is approximately 8 times more selective for the beta 1 receptor than esmolol [17]. Landiolol is metabolised by plasma pseudocholinesterase and liver carboxylesterse to inactive metabolites and its metabolism is unaffected by liver impairment [18]. A few preclinical studies have examined landiolol in sepsis. It decreased circulating levels of the cytokines, TNF-alpha, IL-6, and high mobility group box (HMGB)-1 and reduced histological lung damage in a rat endotoxin model [11]. landiolol was also cardio-protective in septic rats by normalising the expression of cardiac vasoactive peptide endothelin-1 [19]. Landiolol has been used safely and successfully in a septic patient with atrial fibrillation [20] and in sepsis-induced tachycardia [21]. A recent trial of 151

For peer review only - http://bmjopen.bmj.com/site/about/guidelines.xhtml

patients from 54 centres in Japan [22] demonstrated that landiolol could be safely administered to patients with septic shock.

Landiolol rather than esmolol was selected for this clinical trial because of its superior beta-1 specificity and the expectation that switching the landiolol infusion off would allow a rapid return to the baseline haemodynamic state if investigators deemed the intervention harmful. We will also report data of safety of landiolol infusion as part of the study.

Study Objectives

Primary Objective

The primary objective of this trial is to assess the efficacy, safety and mechanisms of landiolol (beta-blockade) in patients with septic shock and tachycardia requiring prolonged (>24 hours continuous treatment) support with high-dose vasopressor agents.

Secondary Objectives

The secondary objectives of the trial are:

- to determine whether infusion of the rapid-acting, ultra-short-lived and highly specific beta-adrenergic antagonist landiolol improves mortality and length of hospital stay compared with current best clinical practice, in patients who have septic shock.
- to investigate the pathways that are altered by beta blockade in septic shock by examining the effects of landiolol on blood markers of inflammation, metabolism and cardiomyocyte damage.

Methods and analysis

This manuscript has been written in concordance with the SPIRIT guidelines [23]

For peer review only

For peer review only - http://bmjopen.bmj.com/site/about/guidelines.xhtml

Trial design

This is a multi-centre, randomised, controlled open label phase IIb trial comparing usual treatment with usual treatment plus landiolol infusion in a total of 340 patients. The trial will be conducted and managed by Warwick Clinical Trials Unit and sponsored by the University of Hospitals of Birmingham NHS Foundation Trust. It is coordinated by a Trial Management Group and independent oversight is provided by the Trial Steering Committee and a Data Monitoring Committee.

The trial has been designed and will be reported in line with the CONSORT (Consolidated Standards of Reporting Trials) statement [24] and the conduct has been planned in full conformance with the principles of the Declaration of Helsinki and Good Clinical Practice.

Study setting

The study recruitment commenced in May 2018. The main trial is taking place in over 35 UK adult intensive care units (ICUs). Participating ICUs will have a typical case mix for UK critical care, a track record of recruitment to clinical trials, suitable support for screening and data collection and the means to store blood samples for transfer to the central analysis unit. They should also be willing to manage atrial fibrillation with correction of potassium, magnesium and amiodarone in the usual care group as the use of beta-blockers in these patients would risk making the trial results uninterpretable.

Inclusion criteria

Patients are eligible to be included in the trial if they meet the following criteria:

- Aged 18 years or above
- Being treated in a Critical Care Unit
- Septic shock according to internationally accepted definitions*
- Heart rate ≥95 bpm (at the time of randomisation)
- Receiving vasopressor support to maintain a target blood pressure for ≥24 hours
- Are being treated with noradrenaline at a rate ≥ 0.1mcg/kg/min

**Sepsis -3 definitions [25]:*

  - confirmed or suspected infection requiring antibiotic therapy
  - new organ dysfunction, as evidenced by an increase in SOFA score ≥2
  - a blood lactate >2 mmol/l at any point during shock resuscitation

For peer review only - http://bmjopen.bmj.com/site/about/guidelines.xhtml

- vasopressor therapy to maintain mean arterial pressure (MAP) ≥65 mmHg

In particular, the presence of a blood lactate > 2 mmol/l is only necessary for the diagnosis of septic shock and is NOT necessary for randomisation 24 hours later.

Exclusion criteria

The participant may not enter the trial if any of the following apply:

- Tachycardia as a result of pain, discomfort from medical devices (including endotracheal tubes), during interventions or other patient distress
- Any form of vasodilatory shock that is not caused by sepsis
- Noradrenaline infusion <0.1mcg/kg/min
- >72 hours after start of vasopressor therapy
- <12 hours since noradrenaline to treat a medical condition other than septic shock stopped
- Having pre-existing severe cardiac dysfunction (NYHA grade 4 or more)
- Having pre-existing severe pulmonary hypertension (mean PA pressures > 55mmHg)
- Acute severe bronchospasm (due to asthma or COPD)
- Untreated second or third degree heart block
- Untreated phaeochromocytoma
- Prinzmetal's angina
- A past history of ischaemic stroke or transient ischaemic attack (TIA) or untreated severe carotid stenosis.
- Advanced liver disease with Child-Pugh Score of ≥B.
- Known sensitivity to beta-blockers
- Patient / legal representative unwilling to provide written informed consent
- Known to be pregnant
- Terminal illness other than septic shock with a life expectancy < 28 days
- Participants who have been administered an investigational medicinal product for another research trial in the past 30 days
- Patients in whom the clinical team feel are about to finish their noradrenaline therapy
- Decision of withdrawal of care is in place or imminently anticipated
- Receiving extracorporeal membrane oxygenation (ECMO) treatment.

Co-enrolment of study participants onto other interventional studies will be considered where there is no possible conflict with the STRESS-L trial objectives.

For peer review only - http://bmjopen.bmj.com/site/about/guidelines.xhtml
For peer review only

Consent

Eligible patients who are deemed competent will be provided with a copy of the patient information sheet before informed consent is sought. However, due to the nature of the underlying condition and its treatment, many patients will be unable to give informed consent. The vulnerability of this patient group is fully appreciated and every effort must be undertaken to protect their safety and well-being. To ensure this, consenting will be obtained in accordance with the Medicines for Human Use Regulations and the Health Research Authority ethics guide on medical research involving adults who cannot consent (http://www.hra.nhs.uk/resources/before-you-apply/consent-and-participation/adults-unable-to-consent-for-themselves/).

Intervention

Participants will be randomised to either usual care (control group) or usual care and landiolol (intervention group).

Usual Care

Participants randomised to this arm will receive usual care for septic shock, namely

- Timely treatment of the source of sepsis (e.g. drainage of infected fluid collections);
- Prompt and appropriate empiric antibiotic treatment, and modification, if needed, on the basis of culture results;
- Appropriate fluid resuscitation to correct hypovolaemia;
- The use of vasopressors to achieve a target mean arterial pressure (suggested target 65-70 mmHg)

Adequate fluid resuscitation will be achieved using repeated fluid challenges to a guided target; the use of a cardiac output monitor is not mandated. The usual care group will not receive any beta blockade for the duration of their ICU stay - if the treating clinician deems beta blockade necessary, this will be captured on the Case Report Form and reported as a protocol deviation. All other general ICU management will be based on the latest guidance from the surviving sepsis campaign [5] and UK national critical care guidelines (e.g. ventilator and central line care bundles).

Usual care and landiolol

For peer review only - http://bmjopen.bmj.com/site/about/guidelines.xhtml

An intravenous infusion of landiolol will be started at 1.0 mcg/kg/min and will progressively increase every 15 minutes at increments of 1.0 mcg/kg/min, to reach the target heart rate of 80-94 bpm usually within 6 hours. Landiolol will be administered peripherally or centrally but must be on a dedicated line. Landiolol has an elimination half-life of 2.3 to 4 minutes [17] and so a loading dose is unnecessary. The landiolol infusion will be continued until the pulse rate is persistently below 95 bpm.

While a patient is receiving vasopressor agents (noradrenaline, vasopressin), the landiolol infusion will be adjusted accordingly to maintain the target heart rate of 80-94 bpm as per the landiolol infusion protocol (Figure 1: STRESS-L Study Drug Infusion Protocol). Once the patient is consistently within the target heart rate of 80-94 bpm, the landiolol Infusion will be continued and not be adjusted. The landiolol should be weaned and, if necessary, stopped whilst the HR is below 80 bpm. Once all vasopressor agents have been discontinued:

- if vasopressors have been discontinued for less than 12 hours, the landiolol infusion will continue (Figure 2: Timing and Weaning of the Study Drug).
- if vasopressors have been discontinued for more than 12 hours, the landiolol should be actively weaned (End of Noradrenaline Treatment EONT timepoint).

This trial allows for up to 14 days of landiolol treatment per participant.

Outcome measures

Primary outcome

The primary outcome will be the mean SOFA score over the first 14 days from entry into the trial and whilst in ICU. Measurement of the SOFA score will cease if the patient withdraws from the trial, dies or is discharged from the ICU. It is assumed that delayed discharges will be evenly distributed between each group.

Secondary outcomes

- Mortality at day 28 and day 90
- Length of ICU and hospital stay

For peer review only - http://bmjopen.bmj.com/site/about/guidelines.xhtml

- Reduction in dose and duration of vasopressor treatment (total daily administered doses)

Mechanistic outcomes

Serial blood samples will be collected from patients and assays will include markers of myocardial dysfunction and inflammation.

- Measurement of Total Catecholamine. It is unknown whether beta-blockade acts through altering the effects of extraneous catecholamines administered as treatment of septic shock or by modulating those produced by the patient. Serum catecholamines will be analysed in the context of the dose of inotropes being administered.
- Markers of myocardial dysfunction. Serum BNP has been demonstrated to be a reliable biomarker of myocardial injury, ischaemia and dysfunction in septic patients and also as a prognostic marker for a poor outcome [26 27]. Serial measurements of Troponin-T will be made.
- Measurement of serum Free Fatty acids and markers of Fatty Acid Metabolism.
- Biomarkers of systemic inflammation. This will be measured using a multiplex inflammatory biomarker assay depending on the available technology at the time of analysis. A selection of cytokines will be analysed, all or some of which will include IL-1beta, IL-2, IL-4, IL-5, IL-6, IL-8, IL-10, IL-12, TNF-alpha, TNF-beta, and IFN-gamma. This focused array will allow an assessment of the pro / anti-inflammatory balance over time in patients with septic shock and allow more detailed study of the other potential mechanisms of action of Landiolol. Cortisol assays will measure the influence of beta blockade on the adrenal cortex
- In addition, samples will be stored for subsequent analysis (e.g. genetics / proteomics / metabolomics) in order to investigate early cellular responses during the resolution of sepsis.

Safety outcomes

- The episodes of Bradycardia (HR <50 bpm), Bradycardia with haemodynamic compromise requiring intervention, significant hypotension requiring intervention (not including temporarily stopping the infusion), heart block, arrhythmia, arrhythmia with haemodynamic compromise requiring intervention will be reported.

For peer review only - http://bmjopen.bmj.com/site/about/guidelines.xhtml

Sample size

The primary outcome is the mean SOFA score over the first 14 days in ICU. Using preliminary collected data from 324 patients from UHB satisfying the trial eligibility criteria, the mean SOFA score over the first 14 days in ICU was 6.3, with standard deviation 2.4. Assuming (conservatively) a standard deviation of 2.8, and a difference of 1 point between the beta blocker and usual care groups, obtaining a p-value less than 0.05 (two-sided) with 90% power would require outcome data on 330 patients. To allow for 3% withdrawals and losses, the proposed sample size is 340 (Figure 3: STRESS-L CONSORT Flow diagram).

For peer review only

Assignment of Interventions

Randomisation

We will use a computerised minimisation randomisation system, created by Warwick Clinical Trials Unit in accordance with their standard operating procedure and held on a secure server, with the allocation generated per individual (participant). Participants will be randomised strictly sequentially as they become eligible for randomisation using a 1:1 ratio. The randomisation will be stratified by recruiting site and noradrenaline dose where the dose reflects the participant's severity. The dose will be dichotomised using a value of 0.3 mcg/kg/min (i.e. ≥0.1 mcg/kg/min to 0.3 mcg/kg/min and >0.3 mcg/kg/min) taken from the LeoPARDS trial as an indicator of low and high severity to ensure participant severity is balanced across both arms [28].

Once written informed consent has been obtained, a member of the local research team will use an interactive voice response (IVR) application to randomise. Eligibility will be confirmed by an investigator prior to randomisation. Sites will only be given access to the application once they have been given the 'green light' to begin recruitment and all required approvals are in place.

Blinding

This an open label trial with no blinding of the treatment allocation. Blinding is not possible due to the requirement for the landiolol dose to be titrated to achieve a target HR.

For peer review only - http://bmjopen.bmj.com/site/about/guidelines.xhtml

Data collection, management and analysis

Data Collection

Screening

All septic shock patients started on noradrenaline in ICU will be screened for trial eligibility. Once eligibility criteria are met there is a 48-hour window for randomisation. Due to this short window, informed consent may be sought during the first 24 hours of noradrenaline therapy. This will allow more time if legal representative consent is required. Randomisation will not occur until vasopressor therapy has been running for ≥24 hours, the patient has been treated with noradrenaline at rate >0.1 mcg/kg/min and remains tachycardic. It will be made clear to the patient or their legal representative that if they no longer meet the eligibility criteria after the 48-hour mark (i.e. 72 hours after the start of vasopressor therapy), the participant will not be randomised.

**Procedure (Time (T) in days/hours)**	**Screening (T0-12)**	**Baseline- (Day 0 - 24hr – T0)**	**Day 1 (T0+24)**	**Day 2**	**Day 3**	**Day 4**	**Day 5**	**Day 6**	**Day 7**	**Day 8**	**Day 9**	**Day 10**	**Day 11**	**Day 12**
Eligibility assessment	●													
Informed consent		●												
Randomisation		●												
Demographics		●												
Medical History		●												
ECG		●	According to clinical need or if AE/SAE											
Pregnancy test		●												
IMP		Dispense												
Blood sample		●	●	●		●		●						
Biobank blood sample (optional)		●	●											
Transport of stored serum														
Local laboratory tests (normal		●	●	●	●	●	●	●	●	●	●	●	●	●
C-Reactive Protein (CRP)		●	●	●	●	●	●	●	●	●	●	●	●	●
Glucose		●	●	●		●		●						
Lactate		●	●	●		●		●						
Worst PaO2/FiO2		●	●	●	●	●	●	●	●	●	●	●	●	●
Platelets		●	●	●	●	●	●	●	●	●	●	●	●	●
Creatinine		●	●	●	●	●	●	●	●	●	●	●	●	●
Bilirubin		●	●	●	●	●	●	●	●	●	●	●	●	●
White Cell Count		●	●	●	●	●	●	●	●	●	●	●	●	●
Liver Function Tests (ALT or AST)		●	●	●		●		●						
Central Venous Blood Gas / Arterial		●	●	●		●		●						
Microbiology results from local lab		●	●	●	●	●	●	●	●	●	●	●	●	●
Heart Rate (Hourly: T0+7 days)		●	●	●	●	●	●	●	●	●	●	●	●	●
Atrial Fibrillation (Hourly: T0+7 days)		●	●	●	●	●	●							
Blood Pressure (Hourly: T0+7 days)		●	●	●	●	●	●							
Rate of Vasopressor / inotropes		●	●	●	●	●	●	●	●	●	●	●	●	●
In/Out Fluids		●	●	●	●	●	●	●	●	●	●	●	●	●
SOFA score		●	●	●	●	●	●	●	●	●	●	●	●	●
Adverse Events (SAEs up to Day 90)		●	●	●	●	●	●	●	●	●	●	●	●	●
Steroid use		●	●	●		●		●						
Compliance		●	●	●	●	●	●	●	●	●	●	●	●	●
Mortality status														
End of Trial														

For peer review only - http://bmjopen.bmj.com/site/about/guidelines.xhtml

Baseline visit 24 hours prior and up to randomisation

Table 1 summarises the schedule of events for the trial. The patient's medical history, physical examination, basic demographic data and steroid use will be recorded. The participant will be randomised, and if allocated to the landiolol arm, IMP will be dispensed and the infusion started. As there is no checking and preparation of drug in the usual care arm, the landiolol infusion should be started within 1 hour of randomisation. The elements of the SOFA score will be recorded along with routine clinical data (cardiovascular, respiratory and renal physiological variables as well as haematological, biochemical and microbiological sample test results). The rates of noradrenaline infusion will be recorded hourly. The site and types of pathogens isolated following admission should be recorded on the eCRF.

Day 1 (time of randomisation to post 24 hours) up to day 14

The elements of the SOFA score will be recorded in the eCRF daily whilst the patient remains in the ICU from randomisation. This may be performed retrospectively but participating centres should ensure that blood has been sent to the Local Hospital Laboratories for the domains that depend on Laboratory Tests (liver, renal and coagulation and C-reactive protein). The rates of noradrenaline infusion and landiolol infusion (if randomised to this group) will be recorded hourly until day 2 to allow comparison of noradrenaline dosing and then 6-hourly thereafter. Heart rate data will be collected to allow assessment of landiolol infusion compliance and separation between groups.

Other clinical data collected will be haemodynamics, respiratory variables (blood gases with the worst P/F ratio, type of ventilator support and previous 24 hours' intravenous fluid intake, urine output, and need for renal replacement therapy (as given in Table 1).

Follow up visits (Days 28 and 90)

Participants will also be followed up to ascertain survival status at 28 days and at 90 days post-randomisation. The participant's Community Physician will be contacted in the first instance to ascertain if the patient is alive; the participant may then be contacted by telephone. SAEs will be reported up to day 90 following randomisation.

For peer review only - http://bmjopen.bmj.com/site/about/guidelines.xhtml

Blood samples

Research blood samples will be collected on Days 0, 1, 2, 4 and 6 and the EONT visit (if this does not fall on a blood sampling day). Day 0 blood samples must be taken prior to the start of landiolol infusion. Days 1, 2, 4, 6 and EONT blood samples can be taken when is convenient within the 24-hour time period. The plasma will be removed and stored as per detailed instructions provided in the trial Laboratory manual. These research blood samples are mandatory as the results are required to answer the secondary outcomes of the trial.

Statistical Analysis

The primary analysis will be conducted according to intention to treat, comparing all those allocated to beta blocker (Landiolol) with all of those allocated to usual care, regardless of treatment received. Regression models will be used to estimate the treatment effects (with 95% confidence intervals), and the models will be adjusted for clinically important covariates. Subgroup effects for baseline severity measured using the dose of noradrenaline (noradrenaline ≥0.1 mcg/kg/min - 0.3 mcg/kg/min vs. >0.3 mcg/kg/min) and use of beta blockers at baseline (Yes/No) will be assessed using formal statistical tests for interaction for the primary outcome and mortality. If there is substantial non-compliance, we will conduct CACE (complier average causal effect) analyses to estimate the treatment effect among those who received the treatment as allocated. The DMC will meet every 6 months to closely monitor the accumulating data, focusing on safety. A detailed statistical analysis plan (SAP) will be written by the study statistical team and then finalised and approved by the DMC before any analysis is undertaken.

Missing data

Every effort will be made to minimise missing outcome data in this trial. Further exploratory analyses to assess the impact of any missing outcome data on the SOFA-score will be examined using multiple imputation techniques and other methods specified in the SAP.

Data Monitoring

The Warwick Clinical Trials Unit will be responsible for trial monitoring and visits will be conducted in accordance with the monitoring plan. On-site monitoring visits during the trial will be check the

For peer review only - http://bmjopen.bmj.com/site/about/guidelines.xhtml

accuracy of data entered into the clinical trial database against the source documents, adherence to the protocol, procedures and Good Clinical Practice and the progress of patient recruitment and follow-up.

Safety, ethics and dissemination

Adverse outcomes

All serious adverse events and suspected unexpected serious adverse events occurring from the time of randomisation to the final follow up visit at day 90 will be recorded on the Case Record Form and faxed or emailed to the coordinating centre within 24 hours of the research staff becoming aware of the event. In particular, bradycardia (HR <50 bpm) with haemodynamic compromise requiring intervention, heart block, significant hypotension requiring intervention, arrhythmia with haemodynamic compromise requiring intervention should be recorded. Adverse event/reactions will be assessed for seriousness and reported in accordance with MHRA guidelines.

Regulatory and ethics approvals

The East of England - Essex Research Ethics Committee (REC: 19/EE/0368 - flagged for trials involving clinical trials in patients without capacity) and Medical Healthcare Regulatory Authority (MHRA) (EudraCT 2017-001785-14) have approved the study protocol. The ICRSTN trial registration number is ISRCTN12600919. The study will comply with the principles for sharing clinical trial data from publicly funded clinical trials.

Dissemination

The study will be reported in accordance with the Consolidated Standards of Reporting Trials (CONSORT) guidelines [24]. The study findings will be presented at national and international meetings with abstracts on-line. Presentation at these meetings will ensure that results and any implications quickly reach all of the UK and international intensive care community. In accordance with the open access policies proposed by the NIHR, we aim to publish the clinical findings of the trial in high quality peer-reviewed open access (via Pubmed) journals. Finally, an on-going update of the trial will

For peer review only - http://bmjopen.bmj.com/site/about/guidelines.xhtml
For peer review only

also be provided on the Warwick Clinical Trials Unit website and social media platforms e.g. Twitter (@STRESSL_trial).

Confidentiality

In order to maintain confidentiality, all CRFs, questionnaires, study reports and communication regarding the study will identify the patients by the assigned unique trial identifier and initials only. Patient confidentiality will be maintained at every stage and will not be made publicly available to the extent permitted by the applicable laws and regulations.

For peer review only

Patient and public involvement

This application is informed through a series of meetings with survivors and carers of patients who have had septic shock. Patient and public contributed to the trial proposal, protocol development and design of patient facing materials. During the conduct of the trial a Patient and Public Involvement (PPI) member has been part of the Trial Management Group and has reviewed patient facing documentation prior to the ethics and regulatory submissions and their comments have been incorporated. Two PPI representatives will sit on the Trial Steering Committee and will provide input from a patient perspective at trial meetings. Both representatives will review and provide feedback on all relevant project documents.

Discussion

Many large multi-centre trials of sepsis and septic shock have not derived statistical differences between study groups following single centre pilot studies suggesting large mortality improvements [29-31]. This is possibly because of the wide variation in severity of illness included in those study. STRESS-L includes patients who are predicted to be at risk of a high mortality because they remain tachycardic (HR >= 95 bpm) after 24 hours of noradrenaline treatment. The persistence of tachycardia may phenotypically define a genetically different cohort as suggested in the Acute

For peer review only - http://bmjopen.bmj.com/site/about/guidelines.xhtml

Respiratory Distress Syndrome (ARDS) population [32]. The trial uses landiolol because of its exceptional short half-life and highly specific beta1 receptor blockade.

The primary outcome measure (mean SOFA score), which correlates with survival, allows measurement of the effect of landiolol on organ function compared with usual care. Analysis of each domain in the SOFA score will also begin to allow us to distinguish whether alteration in cardiac efficiency is the sole mechanism for differences between groups. The collection and analysis of blood samples will also characterise further the role of the adrenergic system in septic shock and proposed genetic predisposition to this specific group of patients with septic shock.

STRESS-L recruited its first patient in April 2018 but a series of protocol amendments (owing to a complex intervention) and the COVID-19 pandemic has delayed the submission of this protocol patient. As of submission, there have been 109 patients recruited, approximately 32% of its target; no COVID-19 patients have been recruited. Our SAP will address how COVID-19 patients (if any) will be analysed. Unpublished data suggest that the inclusion criteria for STRESS-L are rarely achieved in primary COVID-19 illness as the presence of shock (as defined by consensus [25]) coincidental with high dose noradrenaline and a tachycardia within the time constraints are seen in fewer than 1% of patients. Furthermore, our sites have had most of their research staff withdrawn to clinical duties and screening for the study has been suspended during period of exceptionally high admissions.

Contributions

TW and JFB conceived the initial trial concept and helped develop the trial design and protocol. RL, DM and SG carried out the power calculations and with ACG, JL, GDP, DFM, MS, DY helped develop the trial design and protocol. NB, ES, SR oversaw the governance of the protocol. TW is the chief investigator and takes overall responsibility for all aspects of trial design, the protocol and trial conduct. All authors read and approved the final manuscript.

For peer review only - http://bmjopen.bmj.com/site/about/guidelines.xhtml

Competing Interests

TW reports grants from NIHR Efficacy and Mechanism Evaluation for the funding of STRESS-L (Project Number: EME - 14/150/85) and during the conduct of the study; personal fees and non-financial support from AOP Orphan, manufacturer of Landiolol, outside the submitted work; MS has received travel expenses from AOP Orphan for delivering lectures.

MS reports grants and other from NewB, grants from DSTL, other from Amormed, other from Biotest, other from GE, other from Baxter, grants from Critical Pressure, grants from Apollo Therapeutics, other from Roche, other from Bayer, other from Shionogi, outside the submitted work; .

ACG reports receiving grants from the NIHR and the NIHR Research Professorship; nonfinancial support from the NIHR Clinical Research Network and the NIHR Imperial Biomedical Research Centre during the conduct of the study; and personal fees from GlaxoSmithKline and Bristol-Myers Squibb outside the submitted work.

DFMc reports a grant from the NIHR EME programme for this study. Outside the submitted work, DFM reports personal fees from consultancy for GlaxoSmithKline, Boehringer Ingelheim, Bayer, Novartis and Eli Lilly. In addition, his institution has received funds from grants from the NIHR, Wellcome Trust, Innovate-UK and others. In addition, DFM has a patent issued to his institution for a treatment for ARDS. DFM is a Director of Research for the Intensive Care Society and NIHR EME Programme Director.

GP reports grants from National Institute for Health Research, during the conduct of the study; and Director of Research, Intensive Care Society.

All other authors declare they have no competing interests.

Acknowledgements

The authors would like to thank our Patient and Public Involvement (PPI) group and AOP Orphan, Vienna, Austria, for providing the landiolol for the trial free of charge. ACG is the Deputy Director and GDP & DFM are Co-directors of Research for the Intensive Care Foundation. We are grateful for the infrastructure provided by the National Institute of Health Research (NIHR) Critical Care Specialty Group of the Comprehensive Clinical Research Network. We would like to express our gratitude to the members of the Trial Steering Committee and the Data Monitoring Committee who provide support and guidance for this study.

For peer review only - http://bmjopen.bmj.com/site/about/guidelines.xhtml

For peer review only

Funding

This project was funded by the Efficacy and Mechanism Evaluation (EME) Programme and managed by the NIHR (Project Number - 14/150/85). ACG is funded by a NIHR Clinician Scientist Fellowship. MS and GDP are supported by NIHR Senior Investigators. The views expressed in this publication are those of the author(s) and not necessarily those of the EME, NHS, NIHR or the Department of Health.

For peer review only

For peer review only - http://bmjopen.bmj.com/site/about/guidelines.xhtml

References

1. Morelli A, Ertmer C, Westphal M, et al. Effect of heart rate control with esmolol on hemodynamic and clinical outcomes in patients with septic shock: a randomized clinical trial. *JAMA* 2013;310(16):1683-91. doi: 1752246 [pii];10.1001/jama.2013.278477 [doi]
2. Schwartz A, Brotfain E, Koyfman L, et al. Cardiac Arrhythmias in a Septic ICU Population: A Review. *J Crit Care Med (Targu Mures)* 2015;1(4):140-46. doi: 10.1515/jccm-2015-0027 [published Online First: 2015/11/10]
3. Schmidt H, Muller-Werdan U, Hoffmann T, et al. Autonomic dysfunction predicts mortality in patients with multiple organ dysfunction syndrome of different age groups. *Crit Care Med* 2005;33(9):1994-2002. doi: 10.1097/01.ccm.0000178181.91250.99 [published Online First: 2005/09/09]
4. Beesley SJ, Wilson EL, Lanspa MJ, et al. Relative Bradycardia in Patients With Septic Shock Requiring Vasopressor Therapy. *Crit Care Med* 2017;45(2):225-33. doi: 10.1097/CCM.0000000000002065 [published Online First: 2016/09/13]
5. Dellinger RP, Levy MM, Rhodes A, et al. Surviving Sepsis Campaign: international guidelines for management of severe sepsis and septic shock, 2012. *Intensive Care Med* 2013;39(2):165-228. doi: 10.1007/s00134-012-2769-8 [doi]
6. Hayes MA, Timmins AC, Yau EH, et al. Elevation of systemic oxygen delivery in the treatment of critically ill patients. *N Engl J Med* 1994;330(24):1717-22. doi: 10.1056/NEJM199406163302404 [doi]
7. Berk JL, Hagen JF, Beyer WH, et al. The treatment of endotoxin shock by beta adrenergic blockade. *Ann Surg* 1969;169(1):74-81.
8. Suzuki T, Morisaki H, Serita R, et al. Infusion of the beta-adrenergic blocker esmolol attenuates myocardial dysfunction in septic rats. *Crit Care Med* 2005;33(10):2294-301. doi: 00003246-200510000-00021 [pii]
9. Aboab J, Sebille V, Jourdain M, et al. Effects of esmolol on systemic and pulmonary hemodynamics and on oxygenation in pigs with hypodynamic endotoxin shock. *Intensive Care Med* 2011;37(8):1344-51. doi: 10.1007/s00134-011-2236-y [doi]
10. Ackland GL, Yao ST, Rudiger A, et al. Cardioprotection, attenuated systemic inflammation, and survival benefit of beta1-adrenoceptor blockade in severe sepsis in rats. *Crit Care Med* 2010;38(2):388-94. doi: 10.1097/CCM.0b013e3181c03dfa [doi]
11. Hagiwara S, Iwasaka H, Maeda H, et al. Landiolol, an ultrashort-acting beta1-adrenoceptor antagonist, has protective effects in an LPS-induced systemic inflammation model. *Shock* 2009;31(5):515-20. doi: 10.1097/SHK.0b013e3181863689 [doi]
12. Stolk RF, van der Pasch E, Naumann F, et al. Norepinephrine Dysregulates the Immune Response and Compromises Host Defense during Sepsis. *Am J Respir Crit Care Med* 2020;202(6):830-42. doi: 10.1164/rccm.202002-0339OC [published Online First: 2020/06/11]
13. Balik M, Rulisek J, Leden P, et al. Concomitant use of beta-1 adrenoreceptor blocker and norepinephrine in patients with septic shock. *Wien Klin Wochenschr* 2012;124(15-16):552-56. doi: 10.1007/s00508-012-0209-y [doi]
14. Sanfilippo F, Santonocito C, Morelli A, et al. Beta-blocker use in severe sepsis and septic shock: a systematic review. *Curr Med Res Opin* 2015;31(10):1817-25. doi: 10.1185/03007995.2015.1062357 [doi]
15. de ME, Aboab J, Mansart A, et al. Bench-to-bedside review: Beta-adrenergic modulation in sepsis. *Crit Care* 2009;13(5):230. doi: cc8026 [pii];10.1186/cc8026 [doi]
16. Atarashi H, Kuruma A, Yashima M, et al. Pharmacokinetics of landiolol hydrochloride, a new ultra-short-acting beta-blocker, in patients with cardiac arrhythmias. *Clin Pharmacol Ther* 2000;68(2):143-50. doi: S0009923600082473 [pii];10.1067/mcp.2000.108733 [doi]

For peer review only - http://bmjopen.bmj.com/site/about/guidelines.xhtml

17. Iguchi S, Iwamura H, Nishizaki M, et al. Development of a highly cardioselective ultra short-acting beta-blocker, ONO-1101. *Chem Pharm Bull (Tokyo)* 1992;40(6):1462-69.
18. Takahata T, Yasui-Furukori N, Sakamoto J, et al. Influence of hepatic impairment on the pharmacokinetics and pharmacodynamics of landiolol hydrochloride, an ultra-short-acting beta1-blocker. *Drugs R D* 2005;6(6):385-94. doi: 666 [pii]
19. Seki Y, Jesmin S, Shimojo N, et al. Significant reversal of cardiac upregulated endothelin-1 system in a rat model of sepsis by landiolol hydrochloride. *Life Sci* 2014 doi: S0024-3205(14)00405-6 [pii];10.1016/j.lfs.2014.04.005 [doi]
20. Yoshida Y, Hongo T, Sakamoto A, et al. Successful management of tachycardiac atrial fibrillation in a septic patient with landiolol. *Anesth Analg* 2005;100(1):294. doi: 100/1/294-a [pii];10.1213/01.ANE.0000140814.28118.6F [doi]
21. Arita Y, Segawa T, Yamamoto S, et al. Landiolol is effective for the treatment of tachycardia-induced cardiogenic shock in patients during septic shock therapy. *BMJ Case Reports* 2017;2017:bcr-2017-222268. doi: 10.1136/bcr-2017-222268
22. Kakihana Y, Nishida O, Taniguchi T, et al. Efficacy and safety of landiolol, an ultra-short-acting β1-selective antagonist, for treatment of sepsis-related tachyarrhythmia (J-Land 3S): a multicentre, open-label, randomised controlled trial. *Lancet Respir Med* 2020;8(9):863-72. doi: 10.1016/s2213-2600(20)30037-0 [published Online First: 2020/04/04]
23. Chan AW, Tetzlaff JM, Gotzsche PC, et al. SPIRIT 2013 explanation and elaboration: guidance for protocols of clinical trials. *BMJ* 2013;346:e7586. doi: 10.1136/bmj.e7586 [published Online First: 2013/01/11]
24. Moher D, Hopewell S, Schulz KF, et al. CONSORT 2010 explanation and elaboration: updated guidelines for reporting parallel group randomised trials. *BMJ* 2010;340:c869. doi: 10.1136/bmj.c869 [published Online First: 2010/03/25]
25. Singer M, Deutschman CS, Seymour CW, et al. The Third International Consensus Definitions for Sepsis and Septic Shock (Sepsis-3). *JAMA* 2016;315(8):801-10. doi: 2492881 [pii];10.1001/jama.2016.0287 [doi]
26. Charpentier J, Luyt CE, Fulla Y, et al. Brain natriuretic peptide: A marker of myocardial dysfunction and prognosis during severe sepsis. *Crit Care Med* 2004;32(3):660-65. doi: 00003246-200403000-00007 [pii]
27. Post F, Weilemann LS, Messow CM, et al. B-type natriuretic peptide as a marker for sepsis-induced myocardial depression in intensive care patients. *Crit Care Med* 2008;36(11):3030-37. doi: 10.1097/CCM.0b013e31818b9153 [doi]
28. Gordon AC, Perkins GD, Singer M, et al. Levosimendan for the Prevention of Acute Organ Dysfunction in Sepsis. *N Engl J Med* 2016;375(17):1638-48. doi: 10.1056/NEJMoa1609409 [doi]
29. Australian N-SSIft, New Zealand Intensive Care Society Clinical Trials G, the Canadian Critical Care Trials G, et al. Intensive versus conventional glucose control in critically ill patients with traumatic brain injury: long-term follow-up of a subgroup of patients from the NICE-SUGAR study. *Intensive Care Med* 2015;41(6):1037-47. doi: 10.1007/s00134-015-3757-6 [published Online First: 2015/06/20]
30. Venkatesh B, Finfer S, Cohen J, et al. Adjunctive Glucocorticoid Therapy in Patients with Septic Shock. *New England Journal of Medicine* 2018;378(9):797-808. doi: 10.1056/NEJMoa1705835
31. Ranieri VM, Thompson BT, Barie PS, et al. Drotrecogin alfa (activated) in adults with septic shock. *N Engl J Med* 2012;366(22):2055-64. doi: 10.1056/NEJMoa1202290 [doi]
32. Calfee CS, Janz DR, Bernard GR, et al. Distinct Molecular Phenotypes of Direct Versus Indirect ARDS in Single and Multi-Center Studies. *Chest* 2014 doi: 2022041 [pii];10.1378/chest.14-2454 [doi]

For peer review only - http://bmjopen.bmj.com/site/about/guidelines.xhtml

For peer review only

For peer review only - http://bmjopen.bmj.com/site/about/guidelines.xhtml

Figure Legends

Figure 1: STRESS-L Study Drug Infusion Protocol

Figure 2: STRESS-L Protocol for Weaning and Stopping of Study Drug

Figure 3: STRESS-L CONSORT Flow diagram

Table 1: STRESS-L schedule of interventions

For peer review only

For peer review only - http://bmjopen.bmj.com/site/about/guidelines.xhtml

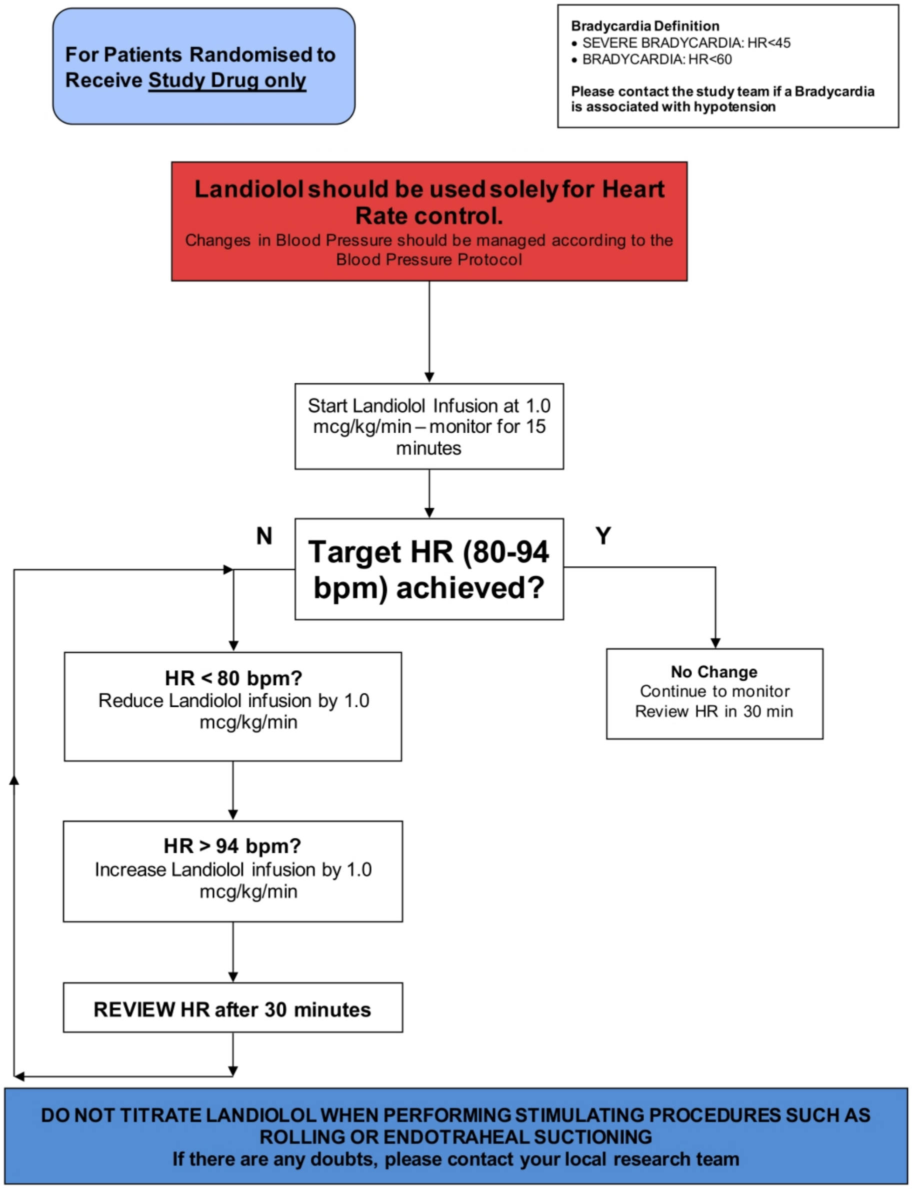

Figure 1: STRESS-L Study Drug Infusion Protocol

197x248mm (299 x 299 DPI)

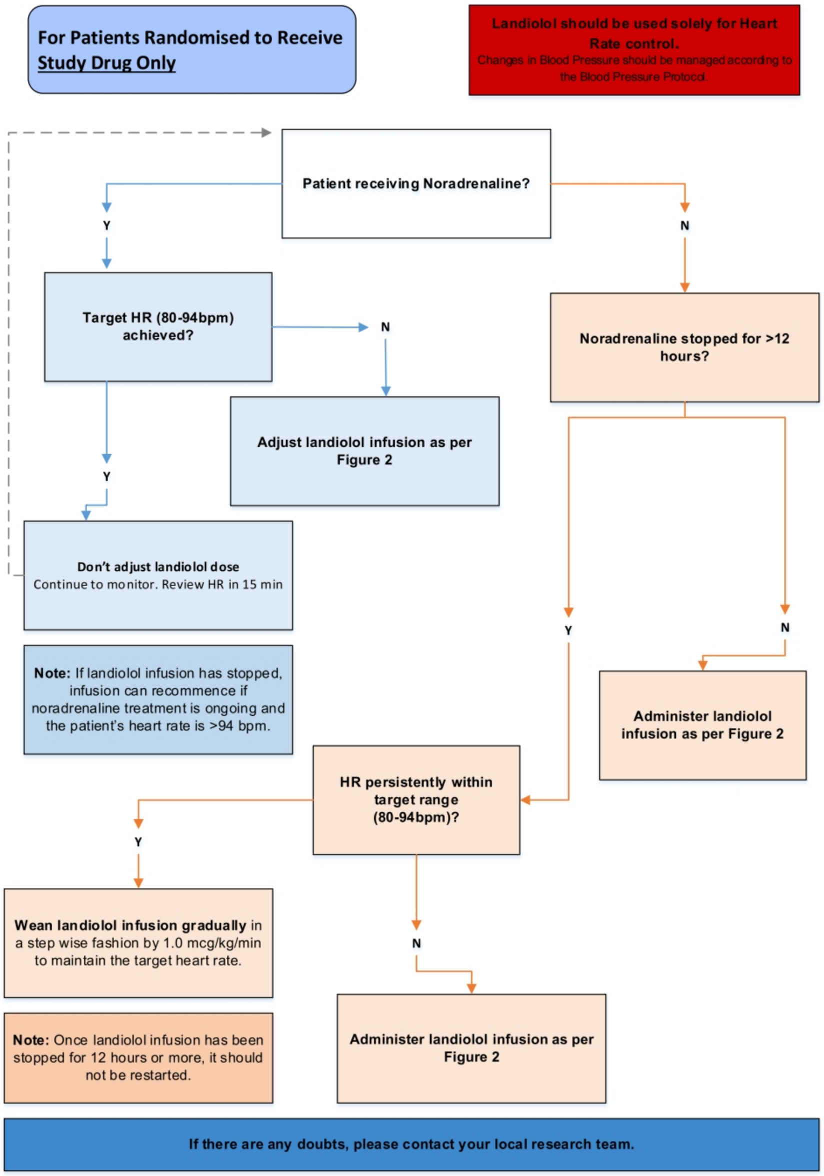

Figure 2: STRESS-L Protocol for Weaning and Stopping of Study Drug

198x267mm (299 x 299 DPI)

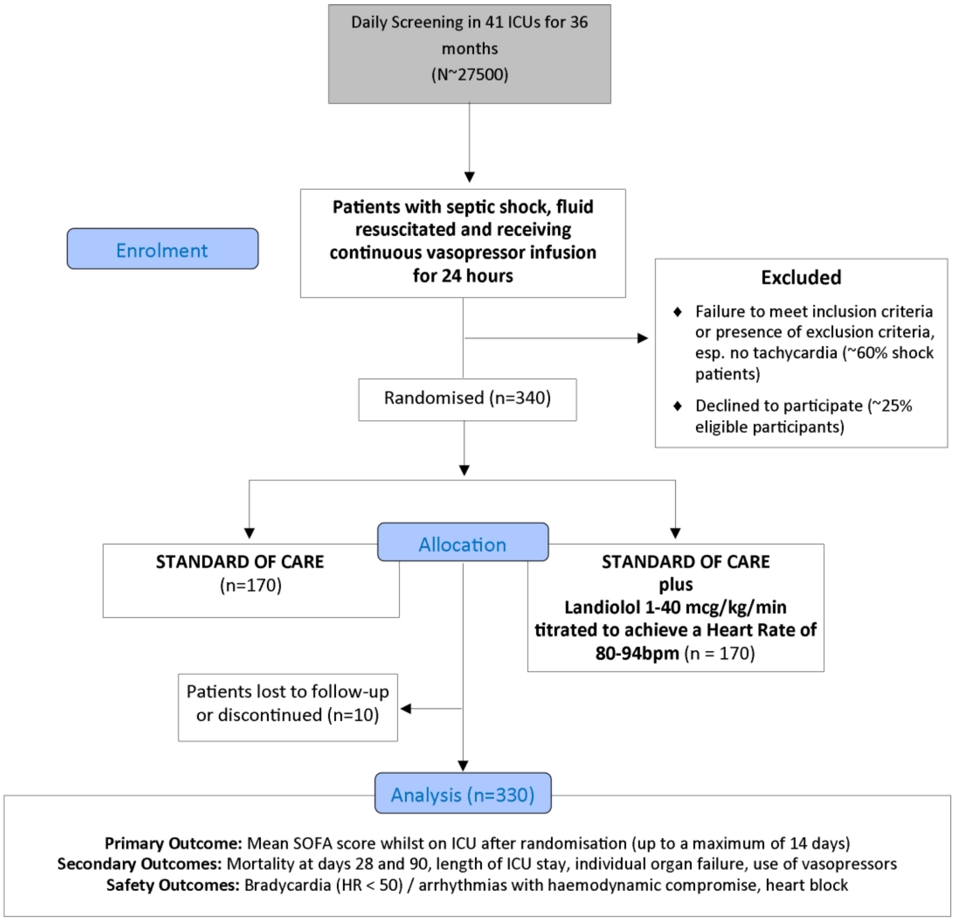

Figure 3: STRESS-L CONSORT Flow diagram

195x196mm (300 x 300 DPI)

STANDARD PROTOCOL ITEMS: RECOMMENDATIONS FOR INTERVENTIONAL TRIALS

SPIRIT 2013 Checklist: Recommended items to address in a clinical trial protocol and related documents*

Section/item	Item No	Description	Addressed on page number
**Administrative information**			
Title	1	Descriptive title identifying the study design, population, interventions, and, if applicable, trial acronym	____1 & 9____
Trial registration	2a	Trial identifier and registry name. If not yet registered, name of intended registry	____1________
	2b	All items from the World Health Organization Trial Registration Data Set	____1 & 52____
Protocol version	3	Date and version identifier	____1________
Funding	4	Sources and types of financial, material, and other support	___1_________
Roles and responsibilities	5a	Names, affiliations, and roles of protocol contributors	___3-4_______
	5b	Name and contact information for the trial sponsor	___3_________
	5c	Role of study sponsor and funders, if any, in study design; collection, management, analysis, and interpretation of data; writing of the report; and the decision to submit the report for publication, including whether they will have ultimate authority over any of these activities	___N/A?______
	5d	Composition, roles, and responsibilities of the coordinating centre, steering committee, endpoint adjudication committee, data management team, and other individuals or groups overseeing the trial, if applicable (see Item 21a for data monitoring committee)	___3-4_______

For peer review only

Introduction

Background and rationale	6a	Description of research question and justification for undertaking the trial, including summary of relevant studies (published and unpublished) examining benefits and harms for each intervention	__14-18______
	6b	Explanation for choice of comparators	__15-21______
Objectives	7	Specific objectives or hypotheses	___24________
Trial design	8	Description of trial design including type of trial (eg, parallel group, crossover, factorial, single group), allocation ratio, and framework (eg, superiority, equivalence, noninferiority, exploratory)	_21, 29, 50, 51_

Methods: Participants, interventions, and outcomes

Study setting	9	Description of study settings (eg, community clinic, academic hospital) and list of countries where data will be collected. Reference to where list of study sites can be obtained	___21 & https://warwick.ac.uk/fac/sci/med/research/ctu/trials/stressl/sites/_
Eligibility criteria	10	Inclusion and exclusion criteria for participants. If applicable, eligibility criteria for study centres and individuals who will perform the interventions (eg, surgeons, psychotherapists)	_9-11, 25-27_
Interventions	11a	Interventions for each group with sufficient detail to allow replication, including how and when they will be administered	_30-36___
	11b	Criteria for discontinuing or modifying allocated interventions for a given trial participant (eg, drug dose change in response to harms, participant request, or improving/worsening disease)	__35-36___
	11c	Strategies to improve adherence to intervention protocols, and any procedures for monitoring adherence (eg, drug tablet return, laboratory tests)	_____________
	11d	Relevant concomitant care and interventions that are permitted or prohibited during the trial	__35-36____

For peer review only

Outcomes	12	Primary, secondary, and other outcomes, including the specific measurement variable (eg, systolic blood pressure), analysis metric (eg, change from baseline, final value, time to event), method of aggregation (eg, median, proportion), and time point for each outcome. Explanation of the clinical relevance of chosen efficacy and harm outcomes is strongly recommended	___24-25_____
Participant timeline	13	Time schedule of enrolment, interventions (including any run-ins and washouts), assessments, and visits for participants. A schematic diagram is highly recommended (see Figure)	__27-30______
Sample size	14	Estimated number of participants needed to achieve study objectives and how it was determined, including clinical and statistical assumptions supporting any sample size calculations	__50_______
Recruitment	15	Strategies for achieving adequate participant enrolment to reach target sample size	___50_______

Methods: Assignment of interventions (for controlled trials)

Allocation:

Sequence generation	16a	Method of generating the allocation sequence (eg, computer-generated random numbers), and list of any factors for stratification. To reduce predictability of a random sequence, details of any planned restriction (eg, blocking) should be provided in a separate document that is unavailable to those who enrol participants or assign interventions	___29______
Allocation concealment mechanism	16b	Mechanism of implementing the allocation sequence (eg, central telephone; sequentially numbered, opaque, sealed envelopes), describing any steps to conceal the sequence until interventions are assigned	___29_______
Implementation	16c	Who will generate the allocation sequence, who will enrol participants, and who will assign participants to interventions	___29______
Blinding (masking)	17a	Who will be blinded after assignment to interventions (eg, trial participants, care providers, outcome assessors, data analysts), and how	__9, 11, 29_____
	17b	If blinded, circumstances under which unblinding is permissible, and procedure for revealing a participant's allocated intervention during the trial	___N/A______

Methods: Data collection, management, and analysis

Data collection methods	18a	Plans for assessment and collection of outcome, baseline, and other trial data, including any related processes to promote data quality (eg, duplicate measurements, training of assessors) and a description of study instruments (eg, questionnaires, laboratory tests) along with their reliability and validity, if known. Reference to where data collection forms can be found, if not in the protocol	48-49 & Electronic CRF
	18b	Plans to promote participant retention and complete follow-up, including list of any outcome data to be collected for participants who discontinue or deviate from intervention protocols	____48-49 ____
Data management	19	Plans for data entry, coding, security, and storage, including any related processes to promote data quality (eg, double data entry; range checks for data values). Reference to where details of data management procedures can be found, if not in the protocol	_48-49, Warwick CTU SOP for Data Management
Statistical methods	20a	Statistical methods for analysing primary and secondary outcomes. Reference to where other details of the statistical analysis plan can be found, if not in the protocol	_50-51, A separate SAP will be published
	20b	Methods for any additional analyses (eg, subgroup and adjusted analyses)	_50-51_______
	20c	Definition of analysis population relating to protocol non-adherence (eg, as randomised analysis), and any statistical methods to handle missing data (eg, multiple imputation)	_50-51_____
**Methods: Monitoring**			
Data monitoring	21a	Composition of data monitoring committee (DMC); summary of its role and reporting structure; statement of whether it is independent from the sponsor and competing interests; and reference to where further details about its charter can be found, if not in the protocol. Alternatively, an explanation of why a DMC is not needed	__4, 45, 46____
	21b	Description of any interim analyses and stopping guidelines, including who will have access to these interim results and make the final decision to terminate the trial	__51______
Harms	22	Plans for collecting, assessing, reporting, and managing solicited and spontaneously reported adverse events and other unintended effects of trial interventions or trial conduct	___25, 38, 42-47___
Auditing	23	Frequency and procedures for auditing trial conduct, if any, and whether the process will be independent from investigators and the sponsor	__49, 54-55___

For peer review only - http://bmjopen.bmj.com/site/about/guidelines.xhtml

Ethics and dissemination

Research ethics approval	24	Plans for seeking research ethics committee/institutional review board (REC/IRB) approval	__52_____
Protocol amendments	25	Plans for communicating important protocol modifications (eg, changes to eligibility criteria, outcomes, analyses) to relevant parties (eg, investigators, REC/IRBs, trial participants, trial registries, journals, regulators)	_52_______
Consent or assent	26a	Who will obtain informed consent or assent from potential trial participants or authorised surrogates, and how (see Item 32)	_27-29____
	26b	Additional consent provisions for collection and use of participant data and biological specimens in ancillary studies, if applicable	__27-29_____
Confidentiality	27	How personal information about potential and enrolled participants will be collected, shared, and maintained in order to protect confidentiality before, during, and after the trial	___49, 54____
Declaration of interests	28	Financial and other competing interests for principal investigators for the overall trial and each study site	_A CoI statement is attached in the paper but is not currently in the protocol__
Access to data	29	Statement of who will have access to the final trial dataset, and disclosure of contractual agreements that limit such access for investigators	__50_____
Ancillary and post-trial care	30	Provisions, if any, for ancillary and post-trial care, and for compensation to those who suffer harm from trial participation	_NHS Standard Care____
Dissemination policy	31a	Plans for investigators and sponsor to communicate trial results to participants, healthcare professionals, the public, and other relevant groups (eg, via publication, reporting in results databases, or other data sharing arrangements), including any publication restrictions	__55_______
	31b	Authorship eligibility guidelines and any intended use of professional writers	_55_____
	31c	Plans, if any, for granting public access to the full protocol, participant-level dataset, and statistical code	_55____

For peer review only - http://bmjopen.bmj.com/site/about/guidelines.xhtml

Appendices

Informed consent materials	32	Model consent form and other related documentation given to participants and authorised surrogates	_https://warwick.ac.uk/fac/sci/med/research/ctu/trials/stressl/health/isf/___
Biological specimens	33	Plans for collection, laboratory evaluation, and storage of biological specimens for genetic or molecular analysis in the current trial and for future use in ancillary studies, if applicable	__39, 41____

*It is strongly recommended that this checklist be read in conjunction with the SPIRIT 2013 Explanation & Elaboration for important clarification on the items. Amendments to the protocol should be tracked and dated. The SPIRIT checklist is copyrighted by the SPIRIT Group under the Creative Commons "Attribution-NonCommercial-NoDerivs 3.0 Unported" license.

For peer review only
